# Catalyst-Free Photoredox Addition–Cyclisations: Exploitation of Natural Synergy between Aryl Acetic Acids and Maleimide

**DOI:** 10.1002/chem.201304929

**Published:** 2014-03-20

**Authors:** David W Manley, Andrew Mills, Christopher O'Rourke, Alexandra M Z Slawin, John C Walton

**Affiliations:** [a] EaStCHEM School of Chemistry, University of St. AndrewsPurdie Building, North Haugh, St Andrews, Fife, KY16 9ST (UK) E-mail: dm53@st-andrews.ac.ukjcw@st-andrews.ac.uk; [b] School of Chemistry and Chemical Engineering, Queen's University BelfastStranmillis Road, Belfast BT9 5AG (UK)

**Keywords:** EPR spectroscopy, heterocyclic compound, NMR spectroscopy, photochemistry, radicals

## Abstract

Suitably functionalised carboxylic acids undergo a previously unknown photoredox reaction when irradiated with UVA in the presence of maleimide. Maleimide was found to synergistically act as a radical generating photoxidant and as a radical acceptor, negating the need for an extrinsic photoredox catalyst. Modest to excellent yields of the product chromenopyrroledione, thiochromenopyrroledione and pyrroloquinolinedione derivatives were obtained in thirteen preparative photolyses. In situ NMR spectroscopy was used to study each reaction. Reactant decay and product build-up were monitored, enabling reaction profiles to be plotted. A plausible mechanism, whereby photo-excited maleimide acts as an oxidant to generate a radical ion pair, has been postulated and is supported by UV/Vis. spectroscopy and DFT computations. The radical-cation reactive intermediates were also characterised in solution by EPR spectroscopy.

## Introduction

Radical methodology has developed into an important tool for synthetic organic chemists by virtue of the fact that highly reactive species can be generated under mild and neutral conditions, often without the need to employ protecting groups.[1–10] The generation of carbon-centred radicals has traditionally been dominated by reductive chain processes mediated by group 14 metal hydrides, particularly tin.[11] Unfortunately, organotin-hydride reagents are notoriously toxic[12, 13] and their residues difficult to remove,[14] thus precluding their use in the synthesis of pharmaceuticals, foodstuffs or other formulations intended for human consumption. Alternatives are now available, but to date the ‘tyranny of tin’ remains strong.[14–19]

Carboxylic acids are well known to homolytically decarboxylate upon photolysis, generating carbon centred radicals in many cases.[20–22] The carboxylate moiety, however, generally needs to be irradiated with short wavelength (typically≤250 nm) UV light,[23] which is undesirable due to its adverse effects on human health[24] and its propensity to degrade organic molecules. A plethora of photosensitising agents and photoredox catalysts have been developed, which facilitate photodecarboxylation at longer wavelengths. Organic molecules such as aromatic ketones,[25] quinones,[26] nitro aromatics[27] and dye molecules[28] as well as catalytic iodine,[24] metal complexes,[29] heavy metals[30] and semiconductors[31] have all been successfully used for this purpose. However, disadvantages include the need to remove these agents following reaction, commercial availability and health concerns in several instances.

Maleimides possess a highly varied and interesting photochemistry. Their [2+2] photochemical dimerisations,[32] along with their [2+2] photocycloadditions with olefins,[33] acetylenes[34] and aromatics[35] have been well documented. More recently, the transformation of a series of N-substituted maleimides to perhydroazaazulenes[36] and the photochemical synthesis of fused, polycyclic 1,3-diazepines from maleimides[37] have been reported to take place through [5+2] photocycloadditions. Photochemical additions of various alcohols to the maleimide C=C bond, furnishing the corresponding succinimides,[38] and preparations of a variety of functionalised nitrogen heterocycles from *N*-silylalkyl maleimides[39] have also been described. Photo-electron-transfers (PET) involving the related phthalimide chromophore, leading to photodecarboxylation are well documented, but the subsequent chemistry is quite different to that described here and usually involves addition to the imide carbonyls.[40–42]

We recently investigated the photoredox reactions of phenoxyacetic acids with various acceptor alkenes mediated by photo-excited TiO_2_.[43] We have now found that suitably functionalised carboxylic acids, in combination with maleimide, will take part in a novel reaction sequence when irradiated with mild UVA and in the complete absence of any extrinsic photoredox catalyst. Herein we report on photochemical tandem addition-cyclisations between maleimide and a series of electron-rich aryloxy- arylthio- and anilino-acetic acids. The particular nature of these reactions enabled us to apply special in situ NMR monitoring of reactant decay and product build-up. We propose an unusual mechanism in which UV energy uptake by maleimide is seamlessly coupled to electron transfer and molecular reorganisation.

## Results and Discussion

### Addition cyclisations of aryloxy-, arylthio- and arylamino-acetic acids with maleimide

Degassed solutions of 4-methoxyphenoxyacetic acid **4 d** and maleimide **1** were irradiated with UVA through Pyrex for a sub-optimum reaction time of five hours in order to determine the ideal stoichiometry and reaction medium. A minimum five-fold excess of **1** was found to be necessary for the reaction to run to completion. When less than five equivalents were used, they were entirely consumed before **4 d** could be fully converted to **5 d**. Succinimide **7** was the only by-product identified, but this accounted for <20 % of **1** used. Upon photolysis it is thought that **1** polymerises in competition with the addition-cyclisation process and this accounts for the rest of the **1** consumed.[44] Maleimides photo-polymerise very readily as they exhibit the capability to act as both photoinitiator and as a polymerisable monomer.[45] At the end of each photolysis, NMR analyses of the product mixtures before purification showed broad signals indicative of maleimide oligomers/polymers, which were consistent with this pathway.

Solvent screening revealed that the reaction proceeded well in polar media, for instance alcohols and acetone, but not at all in less polar solvents such as dichloromethane or benzene. Poor yields were also recorded in water. The optimum reaction medium was found to be 35 % water in acetonitrile. Mariano and co-workers have demonstrated the beneficial effects of this solvent system during the aforementioned study on the intramolecular photochemistry of N-substituted maleimides.[39] When *N*-methylmaleimide was used in place of **1**, its [2+2] dimerisation was the dominant process observed. *N*-Phenylmaleimide, and the related maleic anhydride were found to be unreactive upon photolysis with our setup. These results limit the scope, but are in harmony with previous observations that the maleimide chromophore is highly sensitive to substitution. Thus, maleimide **1** was found to be an essential component. Full details of the optimisation work, can be found in the Supporting Information.

**Scheme 1:**
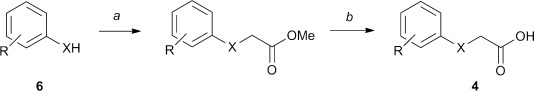
Preparations of aryloxy- and arylthio-acetic acids. X=O/S. a) BrCH_2_CO_2_Me, K_2_CO_3_, THF, reflux; b) LiOH, 3:1 MeOH/H_2_O.

The photochemistry of **1** with a range of aryloxy-, arylthio- and anilino-acetic acids **4** was next examined. Non-commercially available carboxylic acids were prepared by coupling the corresponding phenol or thiol **6** to methyl bromoacetate followed by basic hydrolyses. Compounds **4 g**, **h**, **j** and **k** were prepared in this manner, in yields ranging from 56–85 % (Scheme 1).

In a typical photolysis a solution of 0.75 mmol of acid **4** and five to ten equivalents of **1** in 20 mL 35 % H_2_O/MeCN was degassed by bubbling with argon before being irradiated with UVA using twelve Philips Cleo 15 W tubes. Following the removal of solvent by rotary evaporation, reaction mixtures were analysed using NMR spectroscopy and GC-MS. A tangible advantage was that no catalyst had to be removed. Isolated yields for successful photolyses were obtained after column chromatography and are summarised in Table 1.

**Table 1:** Preparative results for the addition-cyclisation of 4 with 1

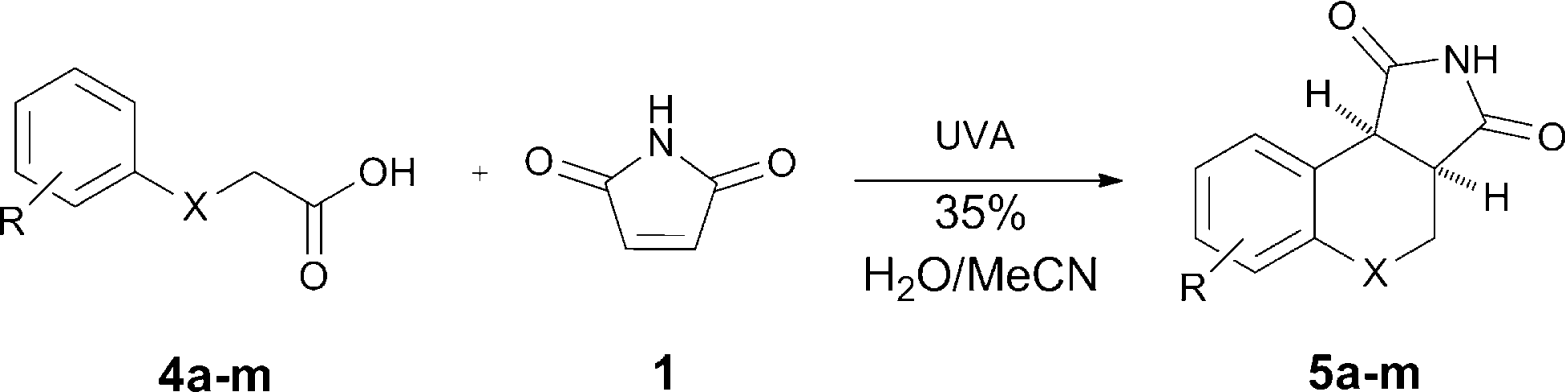					
Entry (acid4)	Ar	Conversion [%]	*t* [h]	Product	Yield [%]^[a]^
1 (**a**)	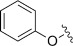	n.d.	18	**5 a**	<5
2 (**b**)	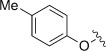	n.d.	25	**5 b**	29^[b]^
3 (**c**)	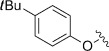	100	18	**5 c**	54^[b]^
4 (**d**)	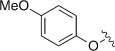	100	18	**5 d**	54
5 (**e**)	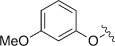	100	18	**5 e**	80^[c]^
6 (**f**)	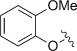	100	18	**5 a**	54
7 (**g**)	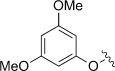	100	18	**5 g**	82
8 (**h**)	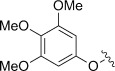	29	64	**5 h**	24
9 (**i**)	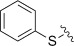	100	18	**5 i**	39
10 (**j**)	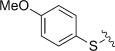	100	72	**5 j**	36
11 (**k**)	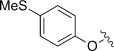	53	72	**5 k**	14
12 (**l**)	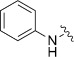	100	18	**5 l**	89
13 (**m**)	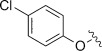	n.d.	18	**5 m**	<5

[a] Isolated yields. [b] 10 equivalents of 1 used. [c] Combined yield of 7-methoxy and 9-methoxy regioisomers; 64 and 16 %, respectively.

Unsubstituted phenoxyacetic acid **4 a** proved unreactive under our preparative conditions although the product **5 a** was detected during in situ NMR experiments (vide infra). Acids **4 b** and **c**, in which the phenyl rings both bear alkyl substituents, yielded the corresponding chromenopyrrolediones **5 b** and **c** with the *tert*-butyl derivative performing better. Both reactions, however, required ten molar equivalents of **1** to achieve full conversion. Methoxy analogues **4 d–f** showed improved reactivity, requiring only five equivalents of **1** and furnished products **5 d–f** in moderate to excellent yields. The 2-methoxy-substituted acid **4 f** was unique in that the methoxy substituent was lost during formation of chromenopyrroledione **5 a**. Possibly cyclisation onto the *ortho-*position bearing the methoxy substituent was followed by dissociative release of methoxyl radicals (alternatively, this might occur by oxidative loss of formaldehyde and a proton from cationic intermediate **14**: see below). 3-Methoxyphenoxyacetic acid **4 e** gave two isomeric products in a combined yield of 80 %. The less sterically crowded 7-methoxy isomer was the major product (64 %) with only 16 % of the 9-methoxy isomer. Inclusion of methoxy substituents at both of the *meta*-positions of **4 g** removed the possibility for the formation of regioisomers and gave the corresponding di-substituted product **5 g** in an excellent yield of 82 %. Disappointingly the 3,4,5-trimethoxy analogue of **4 h**, proved a deal less reactive. Following 64 h irradiation only 24 % of **5 h** was isolated and only 29 % of **4 h** had been consumed.

The heteroatoms on the ring substituents, and *beta* to the carboxylate moiety, were varied next. Unsubstituted phenylthioacetic acid **4 i** was more reactive than its oxy-analogue **4 a**, giving the corresponding thiochromenopyrroledione **5 i** in a moderate yield of 39 % (entry 9). It was anticipated that, as with the phenoxyacetic acids, introduction of a methoxy substituent to the phenyl ring in **4 j** would improve the yield and conversion. However, it turned out that less **5 j** than **i** was actually isolated (entry 10). Acid **4 k**, the methylthio-analogue of **4 d**, also performed comparatively poorly (entry 11). Pleasingly, *N*-phenylglycine **4 l** was converted to the pyrroloquinolinedione derivative **5 l** in an excellent yield of 89 %, greatly outperforming its oxy- and thio- analogues. Lastly, it was decided to probe the effects of the inclusion of electron-releasing substituents in the aryl ring of **4**. This approach proved to be unsuccessful; incorporation of a 4-chloro group in acid **4 m** resulted in <5 % of the desired product **5 m** being observed by NMR spectroscopy (entry 13).

**Scheme 2:**
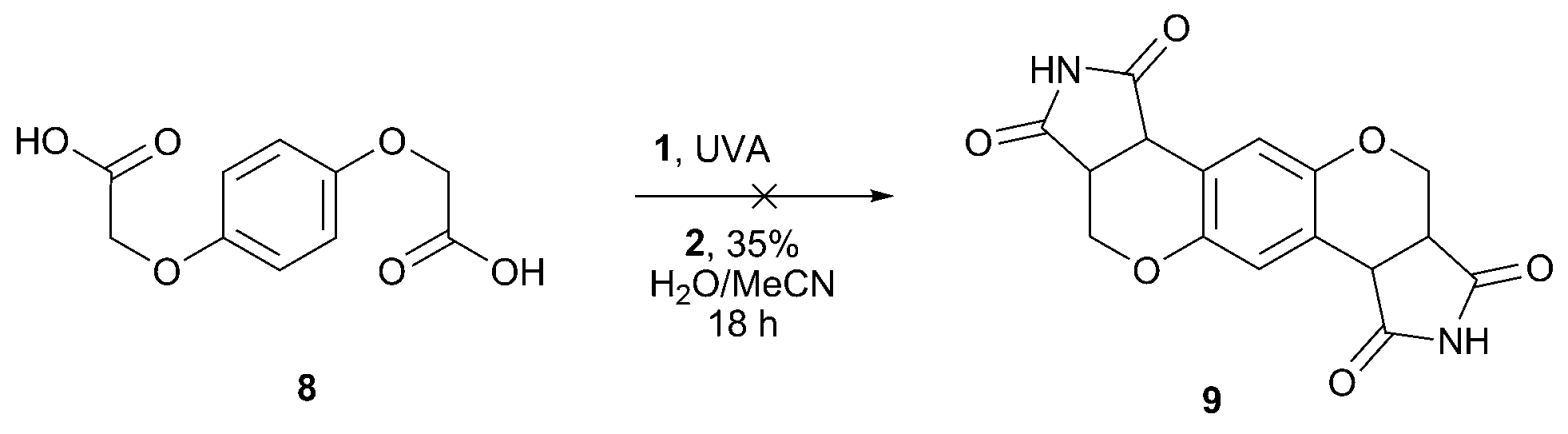
Attempted dual addition-cyclisation between 8 and 1.

It was envisaged that pentacyclic product **9** might be conveniently prepared from commercially available hydroquinone-*O*,*O′*-diacetic acid **8** and **1** in two sequential addition-cyclisations (Scheme 2). **1** and **8** were irradiated for 18 h in 40 mL of 35 % water/acetonitrile. ^1^H NMR analysis revealed only a complex mixture that appeared to be dominated by polymeric material. No signals corresponding to either **9**, or the mono-cyclised intermediate, could be discerned. No signals corresponding to **8** were observed either, suggesting it had been entirely consumed. GC-MS analysis of the reaction mixture was unsuccessful as it was impossible to dissolve a sufficient quantity in a suitable solvent due to its polarity.

**Figure 1:**
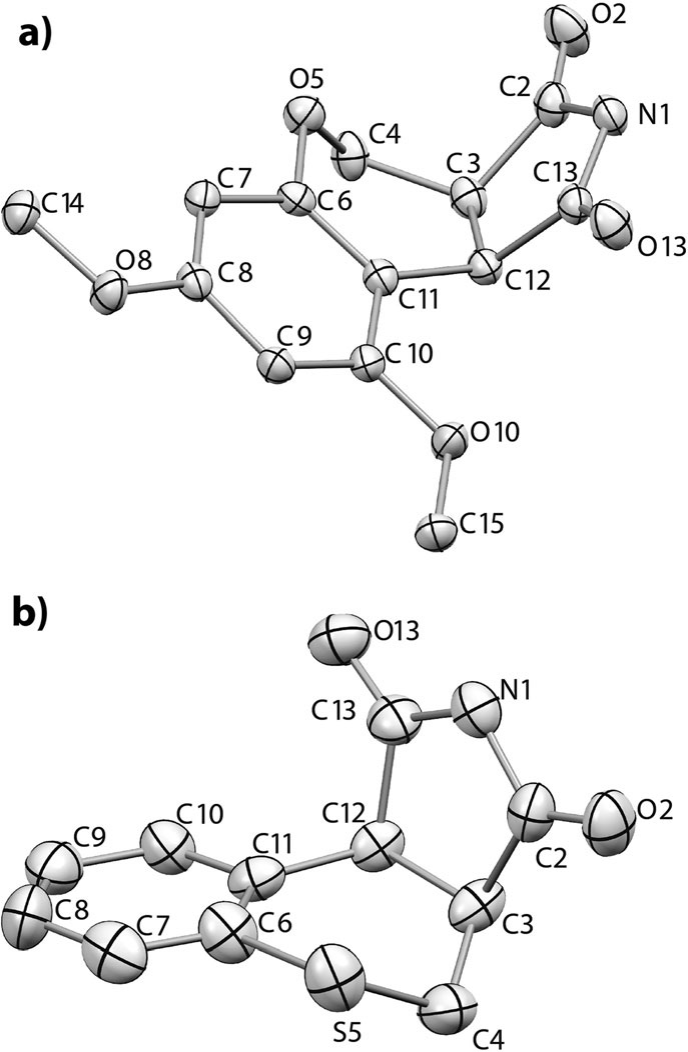
The X-ray crystal structures of a) chromenopyrroledione 5 g and b) thiochromenopyrroledione 5 i. Selected bond lengths, bond angles and torsion angles: 5 g: C3–C4 1.517(2) Å, C11–C12 1.508(2) Å, C2-C3-C4 112.5(1)°, C11-C12-C13, 110.2(1)°, H3-C3-C12-H12 1.3(2)°; 5 i: C3–C4 1.52(2) Å, C11–C12 1.53(1) Å, C2-C3-C4 111.2(9)°, C11-C12-C13, 108.2(9)°, H3-C3-C12-H12 10.0(1)°.

Crystals of suitable quality for X-ray crystallography were obtained for chromenopyrroledione (**5 g**) and thiochromenopyrroledione (**5 i**). The crystallographic structures (Figure 1) confirmed the tricyclic arrangement and showed both **5 g** and **i** adopted a *cis*-geometry at the juncture between the six-membered heterocycle and the pyrroledione ring system. The lengths of the newly formed bonds were close to expectation, falling in the range of 1.50 Å to 1.53 Å. The bond angles between the five and six-membered rings were also as expected, all being between 108° and 113°, indicating that the two carbon atoms at the bridge between the two ring systems were close to tetrahedral. Finally, the torsion angles between the two hydrogen atoms on the junction were observed to be 1.3° and 10.0° for **5 g** and **I**, respectively. From the ^1^H NMR spectra of the isolated products it was observed that the ^3^*J*_H−H_ coupling constants between these two protons all fell within the range of 9.2–9.7 Hz, in agreement with selective formation of the *cis*-isomer in each case.[46]

The fact that this process resulted in the formation of a complex, tricyclic product from relatively simple starting materials under benign conditions, allied to the synergistic cooperation between the acid and maleimide components, marks it as one with good synthetic potential. For example the various modes of ring opening of the pyrole–dione ring system, aminolysis,[47] alcoholysis[48] or reaction with Grignard reagents,[49] to name a few, can be carried out to give diamides, amide/esters and γ-keto amides, respectively. The imide N–H bond is a useful synthetic handle, which could, for instance, be utilised as a nucleophile in the Mitsunobu reaction in order to obtain the N-alkylated derivatives.[49] Treatment with Red-Al could reduce off the carbonyl groups to furnish the corresponding pyrolidines,[50] opening up a variety of new reaction pathways in doing so. The cyclisation fixes the geometry at the ring junction as *cis*, but as there is no further stereo control involved, the products are formed as mixtures of the (*R*,*R*) and (*S*,*S*) diastereomers.

### In situ NMR monitoring of the addition cyclisations

In addition to the preparative irradiations, a series of experiments to monitor reactant consumption and product formation were carried out. In situ NMR spectroscopy was the method of choice as it provided a simple, efficient and effective means of performing the desired measurements; analyses were relatively fast and sampling was not necessary. The reactions were carried out in NMR tubes with irradiations being in a similar manner to preparative work. In place of magnetic stirring, each NMR tube was clamped in a horizontally mounted stirrer and rotated at 250 rpm. The sample was irradiated and the tube removed periodically as needed to perform NMR analyses. The high level of control associated with photochemical processes (i.e., the possibility to stop and start the reaction with the push of a button) made the addition-cyclisation process being studied highly suitable for such an approach. The success of this methodology has recently been demonstrated with the TiO_2_-mediated oxidation of toluene to benzaldehyde and benzoic acid.[51]

The solutions used were identical in composition to those employed in the corresponding preparative reaction, that is, 37.5 mm of the acid **4** and the corresponding amount of **1**. Reactions were carried out on one twentieth the scale using 1 mL of 35 % deuterium oxide in *d_3_*-acetonitrile as the reaction medium. Typical reaction times were reduced from the region of 18 h to 4 h using this photolysis set up. Percentage conversions of maleimide **1** and acids **4** as well as maximum yields and time taken to reach maximum yields for cyclic products **5** and succinimide **7** were successfully measured.

**Figure 2:**
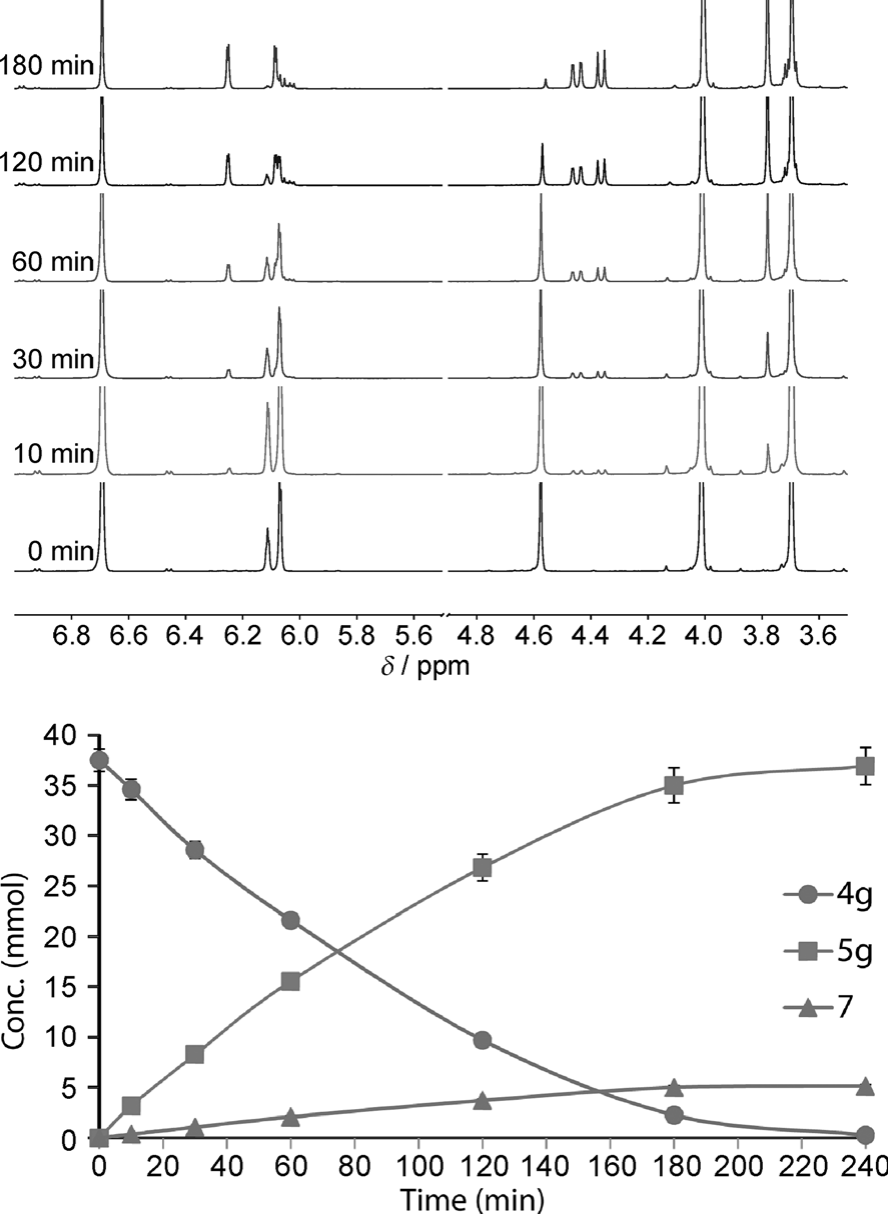
The NMR spectra of the reaction mixture (top) taken at the marked intervals and the reaction profile for photolysis of 1 with 4 g (bottom).

Due to the fact that an internal standard could not be used in the NMR scale irradiations, concentrations of each of the products were determined using the acid as a reference.[52] The ^1^H NMR spectra obtained at timed intervals during irradiation of acid **4 g** are shown in Figure 2(top). These spectra demonstrated how comparatively clean the reactions were. The growth in the signals at *δ*=4.36 and 4.45 ppm and aromatic signals at *δ*=6.25 ppm with photolysis time showed the steady accumulation of the chromenopyrroledione product **5 g**. The diminution of the signals from the acid **4 g** at *δ*=4.56 ppm revealed its corresponding consumption. The resultant growth and decay curves are plotted in Figure 2 (bottom). This illustrates the practically mirror depletion of **4 g** and build-up of **5 g** to a maximum in just 4 h. The addition–cyclisation was accompanied by a steady formation of succinimide **7** as disclosed by the growth of the signal at *δ*=2.61 ppm (not shown in Figure 1). Maleimide **1** depletion has also been omitted from this graph for purposes of clarity. Similar profiles were obtained for all the acids and are available in the Supporting Information. These showed that photo-degradation of the products **5** set in after certain irradiation times (*t*_max_). The maximum yields of chromenopyrroledione **5**, together with the corresponding optimum photolysis times (*t*_max_), derived from the NMR studies are collected in Table 2.

**Table 2:** In situ NMR monitoring data

Entry	Acid4	Conversion4[%]	Max yield5[%]	*t*_max_5[min]	Max yield7[%]	*t*_max_7[min]
1	**a**	88	7	420	>1	540
2	**b**	100	47	180	3	240
3	**c**	100	46	540	3	540
4	**d**	100	59	240	3	300
5	**e**	100	94^[a]^	180	3	240
6	**f**	98	54^[b]^	240	1	240
7	**g**	99	98	240	3	240
8	**h**	5	–	240	–	240
9	**i**	98	41	180	4	240
10	**j**	80	39	420	2	540
11	**k**	41	18	540	>1	540
12	**l**	97	96	90	3	120

[a] Combined yield of 7-methoxy (76 %) and 9-methoxy (18 %) regioisomers. [b] Yield of **5 a** (loss of MeO).

This data demonstrated generally good agreement of yields and conversions with those from the isolated components in the larger scale work (compare Table 2 with Table 1). Product **5 a** was formed from parent compound phenoxyacetic acid **4 a**, albeit in a trivial yield of 7 %. Interesting to note, however, was that 88 % of **4 a** was consumed. This suggests a significant amount of photo-degradation may be taking place. Acids **4 b** and **c** performed significantly better on the smaller scale, achieving yields of 47 and 46 % with only 5 equivalents of **1** (Table 2, entries 2 and 3). Methoxyphenoxyacetic acids **4 d** and **e** performed on a par with the larger scale work. Acid **4 e** furnished the two isomeric forms of **5 e** in a highly pleasing yield of 94 %, displaying the same selectivity towards the 7-methoxy isomer (Table 2, entry 5). The chromenopyrroledione **5 a** from reaction of **4 f** again bore no methoxy substituent following photolysis (entry 6). Acid **4 g**, bearing a 3,5-dimethoxy-substituted ring, was almost quantitatively converted to **5 g** achieving an excellent yield of 98 % with 99 % of **4 g** consumed (entry 7). Unfortunately, compounds **4 h–k** again performed relatively poorly (entries 8–11). By way of contrast *N*-phenylglycine **4 l** again showed excellent reactivity under these conditions with a product yield of 96 % and a conversion of 97 % being recorded in only 90 min (entry 12). Small yields of succinimide **7** were also recorded during each reaction. Full monitoring data and reaction profiles for all reactions are available in the Supporting Information.

### Mechanism of the addition-cyclisation process

**Scheme 3:**
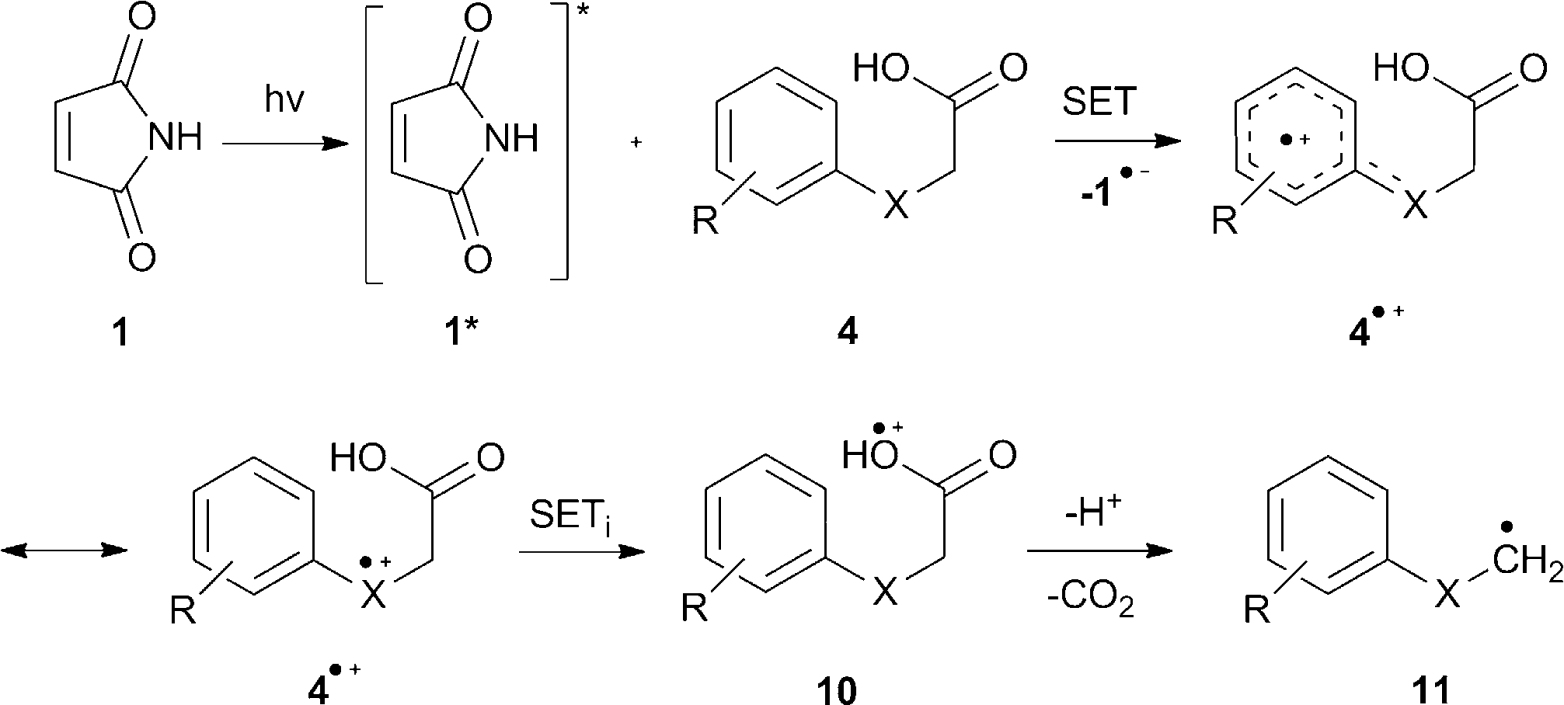
Postulated mechanism for the generation of alkyl radicals 11 from acids 4 through a SET with 1.

**Scheme 4:**
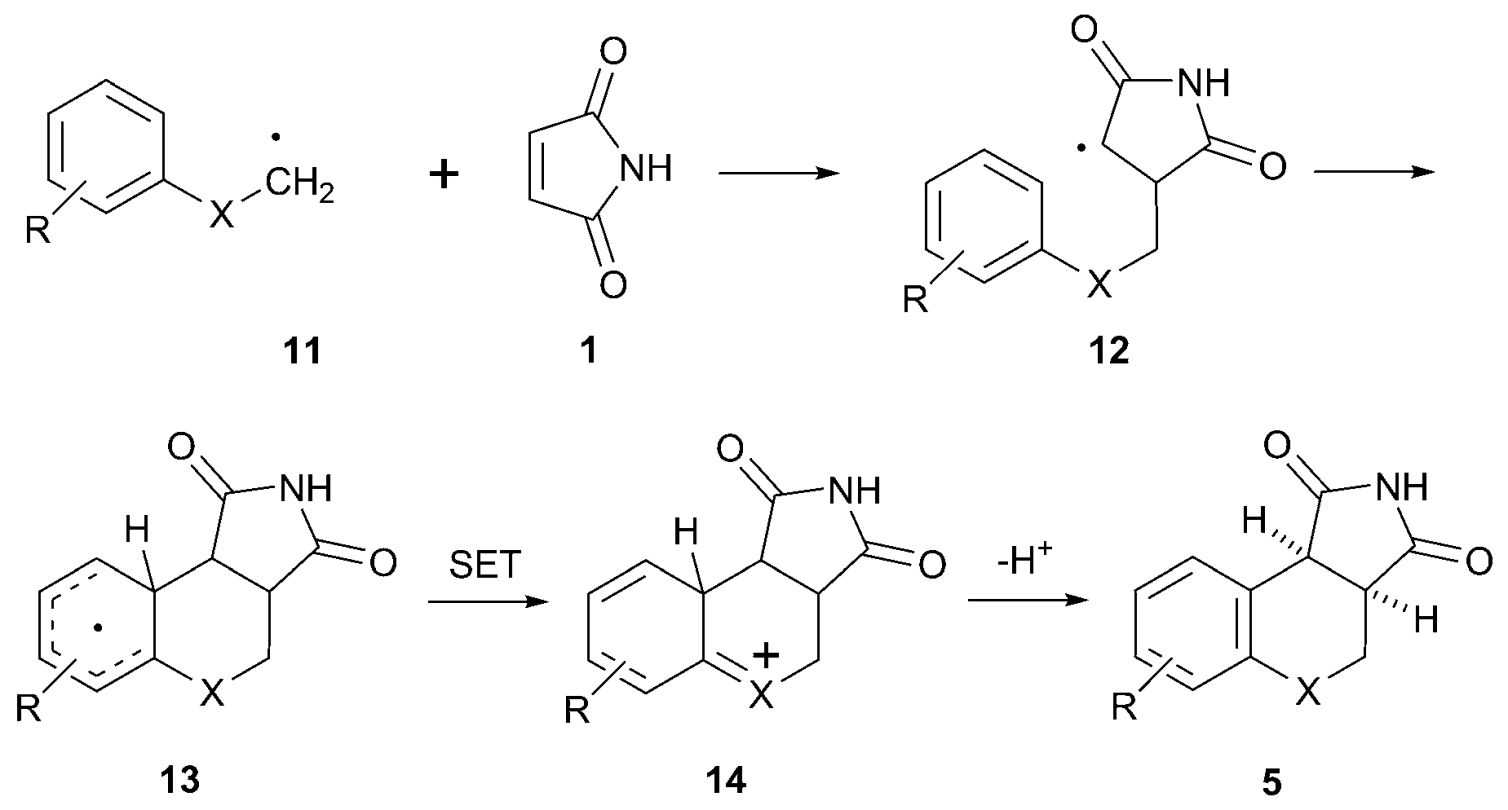
Postulated mechanism for the addition-cyclisation between photo-generated alkyl radicals 11 and 1.

We postulate an unusual and intriguing mechanism as outlined in Schemes 3 and 4. The radical chemistry of maleimides is widely thought to stem from their ability to act as hydrogen abstractors in their excited states.[53] The aforementioned addition of alcohols to maleimides is an example of this.[38] In our case, however, we believe that **1** is operating as a photo-oxidant, generating a radical ion pair (**1**^.−^and **4**^.+^**)** by a single-electron transfer (SET) from **4**. Some precedent exists for this less well-known process. For example, Mariano has proposed that singlet-excited maleimides, as well as related phthalimides, are capable of carrying out such SETs in a series of intramolecular desilylation–cyclisation reactions.[39] Upon photolysis, we believe that **1** transitions from the ground state to its excited state 1* and accepts an electron from the electron-rich aryl ring of **4** giving rise to the aryl radical cation **4**^.+^ and the maleimide radical anion **1**^.−^. This SET is thought to be favoured by π–π stacking interactions between 1* and the aryl ring systems of **4**. The proximity of the carboxylate moiety of **4**^.+^ to the aryl radical cation is such that an internal SET_i_ can easily take place, leading to the formation of unstable acyloxyl radical cation **10**. Precedent for such an internal SET exists; the research group of Young reported that during the reaction of SO_4_^.−^ and a series of phenyl-substituted carboxylic acids an electron transfer took place from the carboxylate functional group to an aromatic radical cation or an intermediate species.[54]

**Figure 3:**
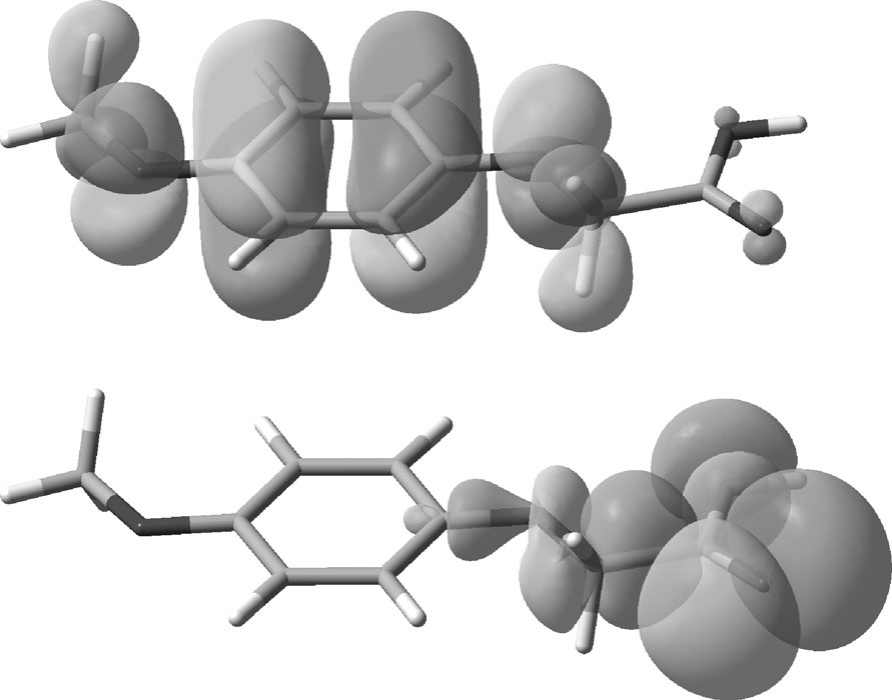
DFT optimised geometries of radical cations 4 d^.+^ and 10. Computed at the UB3LYP/aug-cc-pvtz level: left; HOMO-alpha, right; HOMO-beta.

The maleimide radical anion **1**^.−^ is a known species and has previously been characterised by EPR[55, 56] and UV/Vis[57] spectroscopy having been generated by treatment with reducing agents or photolysis under aqueous conditions. In our system the succinimide by-product of the reaction (**7**) is thought to be formed from **1**^.−^, likely by protonation and subsequent H-abstraction. To shed further light on the mechanism, DFT computations of the geometries and energies of the acids **4** and their radical cations **4**^.+^ were carried out with the Gaussian 09 software suite.[58] The standard B3LYP functional[59] was employed together with 6-311+G(2d,p) and the triple-zeta quality aug-cc-pvtz basis sets. Geometries were fully optimised with the latter basis set. The CPCM polarisable conductor calculation model[60] was then applied, with acetonitrile as the solvent, in an attempt to model the effect of solvent. For the radical cation of **4 d** the two computed frontier orbitals (HOMO-alpha and HOMO-beta, Figure 3) corresponded quite closely to structures **4 d**^.+^ and **10**. These demonstrate the ease by which internal electron migration from the carboxylate moiety into the aryl moiety could take place.

The related phthalimide chromophore is flanked by an aromatic ring whereas **1**, in contrast, has an available electron-deficient C=C bond, thus opening up a new avenue of chemical reactivity. Proton loss from **10**, followed by (or concerted with) β-scission with loss of CO_2_, furnishes nucleophilic alkyl radical **11**. This species subsequently attacks another molecule of **1** and the resultant adduct radical **12** ring closes in *6-endo trig* mode onto the nearby aryl centre. The cyclohexadienyl radical **13** formed then undergoes oxidative re-aromatisation to yield the final product **5** (Scheme 4). Control reactions supported the postulated mechanism. The necessity of light was confirmed by stirring **1** and **4 d** overnight in the dark; subsequent ^1^H NMR analysis and GC-MS revealed only unreacted starting materials present. Acid **4 d** alone was similarly unreactive on photolysis through Pyrex; confirming the necessary presence of **1** in order for photoredox chemistry to take place. As expected, photolysis of **15**, the benzyl ester of **4 d**, furnished **15**^.+^ in the EPR experiments (vide infra), but proved unreactive when photolysed with **1**, because the decarboxylation pathway was blocked. Furthermore the reaction proceeded much more favourably in polar reaction media and this is a hallmark of electron-transfer processes.[61–63]

The ionisation energies (IE) of the acids, obtained from the B3LYP/aug-cc-pvtz level computations in vacuum, correlated well with the computed IEs including CPCM (solvent=acetonitrile). Both sets of IEs decreased as the electron-releasing nature of the ring substituents increased (Table 3). Interesting to note was that *cisoid*- and *transoid*-conformers of **4 d**, **h**, **j** and **k** were computed to have slightly different IE values. As expected, radical cation formation was easier as more electron-donating groups substituted the ring. However, the IE data did not correlate with the yields from the synthetic experiments. For instance, acids **4 h**, **j** and **k** have three of the lowest computed IE values of all the acids. However, they also represent three of the poorest yields observed during the synthetic work. We conclude that product formation is not entirely controlled by the ease of radical-cation formation. In these three cases it is thought that the aryl radical cations are stabilised by the high degree of electron density that is present in the system, thus inhibiting the subsequent internal SET to **10** and/or deprotonation with decarboxylation. Alternative reactions of **4**^.+^ such as back donation of an electron (to **1**^.−^ for instance) become competitive. In some systems, particularly **4 i–k**, low yields of **5** were recorded even though acid conversions were high (Table 1). GC-MS analyses of reaction mixtures revealed only one additional product; the corresponding phenols or thiophenols ArXH. We attribute this to a competing dissociation of radical cations **4**^.+^ to ^.^CH_2_CO_2_H radicals (or CH_3_^.^ radicals for **4 k**^.+^) and an aromatic cation, which is reduced by **1**^.−^ and converts to ArXH after H-transfer. This is expected to compete particularly well for **4 i–k**, which contain weaker S–C bonds (see Tables 1 and 2, entries 9–11).

**Table 3:** DFT IEs and UV/Vis data of 1 and acids 4

Entry	Compound	Ionisation energy [eV]^[a]^	*λ*_max_ [nm]	*λ*_cutoff_^[b]^ [nm]	*ε* [L mol^−1^ cm^−1^]
1	**1**	–	269	351	652
2	**4 a**	6.28	269	309	971
3	**4 b**	6.00	281	294	1225
4	**4 c**	6.04	272	303	1229
5	**4 d**	5.69, 5.67^[c]^	286	321	2529
6	**4 e**	6.01	278	291	1708
7	**4 f**	5.80	273	292	2119
8	**4 g**	6.01	246	286	482
9	**4 h**	5.17, 5.35^[c]^	273	296	1099
10	**4 i**	5.99	246	302	6106
11	**4 j**	5.53, 5.53^[c]^	255	320	7307
12	**4 k**	5.53, 5.52^[c]^	296	323	1295
13	**4 l**	5.49	294	321	2120

[a] DFT method: UB3LYP/aug-cc-pvtz/CPCM-acetonitrile; IE values corrected to 298 K. [b] Cutoff taken to be the point at which absorbance ≤0.5 A.U. [c] *cisoid*- and *transoid*- conformers, respectively (see text).

Molar absorptivites of each of the acids **4**, and **1** were determined using a range of concentrations from 10 mm down to 0.02 mm in acetonitrile, each measured using a 5 mm quartz cuvette. Absorbance below 0.5 atomic units was deemed to be negligible and in this way absorbance cut-offs (λ_cutoff_) were calculated (Table 3). Scrutiny of the lamp output profile of the photo-reactor and of the absorbance profile of Pyrex (Supporting Information) show that below approximately 325 nm no light can enter the reaction vessel. Examination of the UV/Vis data of all compounds used (Table 3) confirmed that **1** was indeed the only compound capable of adsorbing photons during the reactions and thus was the only photoactive species present. None of the acids **4** was capable of adsorbing photons in the setup used and all were thus incapable of directly taking part in any photochemical processes.

**Figure 4:**
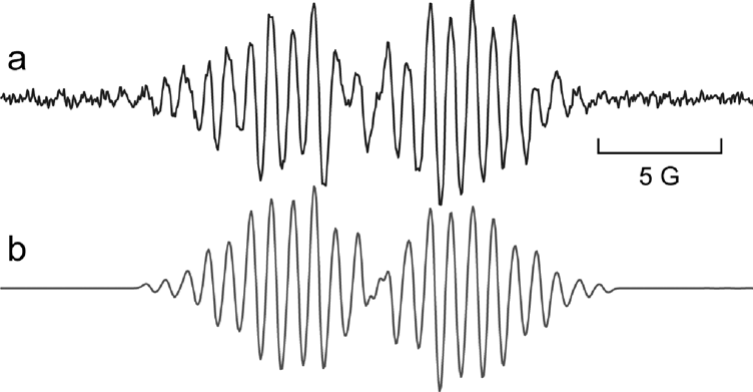
EPR spectra from photolysis of acid 4 d in PhH at 300 K. a) Experimental spectrum from 4 d. b) Computer simulation.

Photolyses of acids **4** were studied by 9 GHz EPR spectroscopy to characterise intermediates generated in solution. Samples of each arylacetic acid (∼10–20 mg) alone were dissolved or dispersed in solvent (0.5 mL) deaerated by bubbling N_2_ for 15 min and then irradiated directly in the spectrometer resonant cavity. In initial experiments, acetonitrile was used as solvent; but only very small samples in capillary tubes could be examined due to microwave absorption by the solvent. No well-defined spectra were obtained until much larger samples in benzene or *tert*-butylbenzene solvent were employed. Figure 4 a shows the weak spectrum obtained from **4 d** after accumulating 20 scans at ambient temperature. This spectrum was satisfactorily simulated (Figure 4 b) with the EPR parameters listed in Table 4.

**Table 4:** Experimental and DFT computed EPR parameters for radical cations of aryloxy-, arylthio-acetic acids and esters.^[a]^

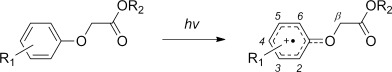											
Entry	Radical cation	R^1^	R^2^	Method (expt./DFT^[b]^)	*g-*factor	*a*(2 H^β^)^[c]^	*a*(H^2^)	*a*(H^3^)	*a*(H^4^)	*a*(H^5^)	*a*(H^6^)
1	4b^.+^	4-Me	H	Expt.	2.0036	6.3	3.5	1.0	14.3 (3 H)	1.0	2.6
2	4b^.+^	4-Me	H	DFT	–	7.8	−4.2	0.6	16.7 (3 H)	−1.2	−2.5
3	4c^.+^	4-*t*Bu	H	Expt.	2.0030	6.7	3.4	0.7	0.8 (9 H)	1.2	2.1
4	4c^.+^	4-*t*Bu	H	DFT	–	7.4	−4.2	0.4	0.9 (9 H)	−1.6	−2.0
5	4d^.+^	4-MeO	H	Expt.	2.0039	2.6	1.9	0.8	1.7 (3 H)^[c]^	4.8	0.8
6	4dt^.+^	4-MeO^[d]^	H	DFT	–	6.4	−2.7	−1.2	4.4 (3 H)^[c]^	−3.1	−0.9
7	4dc^.+^	4-MeO^[d]^	H	DFT	–	6.1	−1.3	−1.3	4.2 (3 H)^[c]^	−2.6	−2.6
8	15^.+^	4-MeO	Bn	Expt.	2.0041	3.3	1.7	0.7	1.7 (3 H)^[c]^	4.9	0.7
9	4h^.+^	3,4,5-triMeO	H	Expt.	2.0040	5.2	1.3	0.4 (3 H)^[c]^	0.9 (3 H)^[c]^	0.4 (3 H)^[c]^	0.9
10	4h^.+^	3,4,5-triMeO	H	DFT	–	3.5	−1.5	1.6 (3 H)^[c]^	5.7 (3 H)^[c]^	2.1 (3 H)^[c]^	−0.1
11	15^.+^	3,4,5-triMeO	Me	Expt.	2.0037	4.1	3.2	0.4 (3 H)^[c]^	1.1 (3 H)^[c]^	0.7 (3 H)^[c]^	1.1
12	4k^.+^	4-MeS	H	Expt.	–	2.8	1.0	1.4	5.2 (3 H)^[c]^	1.8	1.0
13	4kt^.+^	4-MeS	H	DFT	–	4.8	−1.7	−1.1	7.1 (3 H)^[c]^	−2.5	−0.5
14	4kc^.+^	4-MeS	H	DFT	–	4.5	−0.7	−1.5	6.9 (3 H)^[c]^	−2.2	−1.5
15	18^.+[e]^	–	Bn	Expt.	2.0039	3.4	1.5	1.1	2.6 (2 H)^[c]^	1.1	1.5

[a] Spectra in PhH solution at 300 K; hfs in Gauss. [b] DFT computations at the UB3LYP/6-311+G(2d,p) level of theory; note that the signs of hfs cannot be obtained from CW EPR spectra. [c] Hfs from beta-H-atoms are very sensitive to the dihedral angle subtended about the C–X bond by the three bonds linking the H-atom to the ring. The computed hfs are the values obtained for the single dihedral angle of the optimum geometry found by the DFT computation whilst the experimental EPR hfs are actually average values from all the dihedral angles the radicals access as they undergo internal motions in solution. This is the cause of the poor agreement. [d] *Transoid* and *cisoid* structures. [e] Dibenzyl ester of di-acid **8**.

We attribute the spectrum of Figure 4 a to the radical cation of **4 d**. This is thought to form by direct photo-ionisation of **4 d** upon photolysis within the spectrometer cavity. When **4 d** was irradiated through Pyrex, little to no signal was observed. We attribute this to the fact that the high energy light necessary to achieve the direct photo-ionisation of **4 d** could not penetrate the sample tube under these conditions. When **1** was included in an otherwise identical sample of **4 d** in benzene and irradiated under the same conditions, again no signal was observed. It is our belief that the SET process from **4 d** to **1** is disfavoured by the non-polar nature of the benzene solvent used. Irradiation of **1** in the presence of tetrathiafulvalene (TTF; **16)**, a known electron donor, inside the resonant cavity of the spectrometer led to the observation of a strong signal corresponding to the TTF radical cation.[64] No signal was observed when **1** with **16** was examined in the dark, or when **16** was irradiated alone, thus confirming the ability of **1** to act as a photo-oxidant under our conditions. Although we did not observe the EPR spectrum of the maleimide radical anion **1**^.−^ this should not be taken as evidence against its participation. The maleimide radical anion **1**^.−^ is transient[55] so its concentration would be below the detection level under our conditions. On the other hand the **4**^.+^ radical cations are comparatively persistent so their concentration builds to detectable levels.

**Scheme 5:**
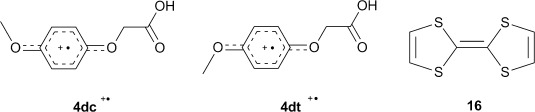
The *cisoid* and *transoid* radical cations of 4-methoxyphenoxyacetic acid 4 d and tetrathiafulvalene (TTF) 16.

Comparison of our hyperfine splittings (hfs) and *g*-factors with data available in the scientific literature enabled the possibility that we were observing spectra derived from phenoxyl[65] or cyclohexadienyl radicals[66] to be discounted. Radical cations of acids **4** had not previously been characterised by EPR spectroscopy, but the *g*-factor and hfs were very similar to those reported for the radical cations of dimethoxybenzene.[67–70] The aromatic core of this model compound is similar to that of acid **4 d**. A pair of radical cations, with *transoid-* and *cisoid*-conformations of the two methoxy groups, having slightly different EPR spectra, were described for the oxidation of 1,4-dimethoxybenzene. However, very high resolution, not achieved in our spectra, was required to distinguish the two. DFT computations[41] of the structures and energies of the *transoid* and *cisoid* conformers of the radical cations **4 d***t*^.+^ and **4 d***c*^.+^ gave hfs in reasonable accord with experiment (Scheme 5, Table 4) and supported our identification. The difference in energy of the two conformers was computed to be <0.5 kcal mol^−1^ with a sizable internal rotation barrier (twofold rotor with a 12 kcal mol^−1^ barrier, see the Supporting Information). It is probable that all our spectra are weighted averages of such conformers. When a sample of 1,4-dimethoxybenzene in benzene was irradiated under our conditions an EPR spectrum very similar to those in the literature for the radical cation pair was observed (Supporting Information). This was further support that the spectra from acids **4** were due to radical cations.

Generally similar spectra were obtained on irradiation of samples of **4 b**, **c**, **h** and the thio-analogue **4 k** alone. No signals were discernible with samples of the unsubstituted acid **4 a** or the thio-analogue **4 i**. Broad spectra with *g*-factors of 2.0039, 2.0036 and 2.0034, appropriate for radical cations, were obtained from **4 e**–**g**. Possibly the poor resolution was due to broadening from exchange processes. Interestingly, EPR spectra of the corresponding radical cations were also observed on photolysis of benzene solutions of **15**, the benzyl ester of **4 d**, **17**, the methyl ester of **4 h** and the dibenzyl ester of diacid **8** (Table 4, entries 8, 11, 15). Obviously these ester radical cations cannot deprotonate, lose CO_2_ and generate the alkyl radicals **11**.

Our acid derived radical cation spectra were much weaker than those described in the literature for alkoxybenzenes. The reason for this is that our species are believed to be formed as the result of direct photo-ionisation of acids **4**, which is inefficient in the non-polar media used. In contrast, literature alkoxybenzene radical cations were all observed on treatment of the parent alkoxybenzene with an oxidising agent in a polar solvent. Our EPR observations of radical cations **4**^.+^ from most of the acid substrates during photolyses support our mechanism with these species as important intermediates.

## Conclusion

We have discovered a distinctive new reaction sequence for electron-rich aryloxy-, arylthio- and arylamino-acetic acids whereby they decarboxylate, releasing alkyl radicals at a benign wavelength of light in the absence of a conventional photoredox catalyst. Maleimide synergistically acts as a radical generator and as a radical acceptor instigating a tandem addition-cyclisation process. Preparative scale irradiations enabled oxa-, thia- and aza-tricyclo pyrroledione derivatives to be isolated. These products can therefore be prepared from relatively simple, readily available precursors and selectively form the *cis*-isomer in each case. The lack of necessity of an extrinsic photoredox catalyst is highly pleasing from the viewpoint of subsequent purification as well as cost, availability and safety. Each reaction has been monitored by in situ NMR spectroscopy, allowing reaction profiles to be obtained for each photolysis.

A plausible mechanism highlighting the key role of maleimide as the photoactive species was presented. SET from the acids to excited maleimide yielded radical cations that de-protonated and lost CO_2_ thus supplying neutral C-centered radicals, which took part in an addition–cyclisation cascade. Several of the novel acid-derived radical cations were characterised by EPR. Maleimide was the essential UV-absorbing component as confirmed by UV/Vis spectroscopic investigations. Aryloxy-, arylthio- and arylamino-acetic acids were all suitable reaction partners, which yielded the corresponding radical cations. Balance was required in the selection of substituents in the aromatic rings. Phenoxyacetic acid, with no ring substituent, and the 4-chloro-derivative with an electron-withdrawing substituent, delivered virtually none of the cascade product. Electron-releasing substituents in the aryl rings favoured this step. However, over-substitution, as with the trimethoxyphenoxy-acid **4 h**, endowed the radical cations with inordinate stabilisation thus inhibiting dissociation and release the ArXCH_2_^.^ radicals needed to set off the cascade.

## Supporting Information

As a service to our authors and readers, this journal provides supporting information supplied by the authors. Such materials are peer reviewed and may be re-organized for online delivery, but are not copy-edited or typeset. Technical support issues arising from supporting information (other than missing files) should be addressed to the authors.

miscellaneous_information
