# Supplementary material for: Catalyst-Free Photoredox Addition–Cyclisations: Exploitation of Natural Synergy between Aryl Acetic Acids and Maleimide
Source: Chemistry. 2014 Mar 20;20(18):5492–500. doi: 10.1002/chem.201304929 (PMC4506563; doi:10.1002/chem.201304929)

CHEMISTRY
A European Journal

Supporting Information

© Copyright Wiley-VCH Verlag GmbH & Co. KGaA, 69451 Weinheim, 2014

Catalyst-Free Photoredox Addition–Cyclisations: Exploitation of Natural Synergy between Aryl Acetic Acids and Maleimide

David W. Manley,*[a] Andrew Mills,[b] Christopher O'Rourke,[b] Alexandra M. Z. Slawin,[a] and John C. Walton*[a]

chem_201304929_sm_miscellaneous_information.pdf

Table of Contents

General Experimental Section S3

Scheme S1. Preparation of carboxylic acids S3

Optimisation Studies S9

Scheme S2. Test reaction for optimisation studies. S9

Preparative Irradiations S12

X-ray Crystallography S21

Fig. S1. The X-ray crystal structure of **5g**. S21

Table S1. Crystal data & structure refinement for **5g** S21

Fig. S2. The X-ray crystal structure of **5i**. S22

Table S2. Crystal data & structure refinement for **5i** S22

Table S3. Coupling Constant between Protons at Ring Junction S24

***In-situ* NMR Monitoring** S25

Fig S3. Irradiation setup for *in-situ* NMR Monitoring S25

Fig S15. Profile showing Lamp Output and Pyrex Absorbance S38

EPR Spectroscopy S39

DFT Optimised Structures S44

DFT Optimised Energies S58

References S64

^{1}H & ^{13}C NMR Spectra for novel compounds S65

General Experimental Section

All reagents and solvents were purchased from either Sigma Aldrich, Alfa Aesar or TCI Europe and used without further purification. Tetrahydrofuran was distilled over sodium and dichloromethane was distilled over calcium hydride. Column chromatography was carried out using Silica 60A (particle size 40-63 µm, Silicycle, Canada) as the stationary phase, and TLC was performed on precoated silica gel plates (0.20 mm thick, Sil G UV_{254}, Macherey-Nagel, Germany) and observed under UV light. ^{1}H and ^{13}C NMR spectra were recorded on Bruker AV III 500, Bruker AV II 400 and Bruker AV 300 instruments. Chemical shifts are reported in parts per million (ppm) from low to high frequency and referenced to the residual solvent resonance. Coupling constants (*J*) are reported in hertz (Hz). Standard abbreviations indicating multiplicity were used as follows: s = singlet, d = doublet, t = triplet, dd = double doublet, q = quartet, m = multiplet, b = broad. Melting points (M.p.) were determined using a Sanyo Gallenkamp apparatus and are reported uncorrected. Mass spectrometry was carried out at the EPSRC National Mass Spectrometry Service Centre, Swansea, UK. UV/Vis spectrometry was carried out using a Cary 50 spectrophotometer (Varian Inc.).

General Procedure for the Preparation of Phenoxy- and Phenylthio-Acetic Acids

To a solution of the phenol/thiol (1 equiv.) in anhydrous THF was added methyl bromo acetate (2 equiv.) and potassium carbonate (5 equiv.) at room temperature. The resultant mixture was refluxed at 80°C for 48-72 hours. The mixture was concentrated under reduced pressure, dissolved in 100 mL CH_2Cl_2, washed with H_2O (3 x 100 mL), dried over $MgSO_4$ and the solvent removed under reduced pressure. To a solution of the resultant ester (1 equiv.) in $MeOH/H_2O$ (3:1 v/v) was added LiOH (5 equiv.) at room temperature and allowed to stir overnight. The reaction mixture was concentrated under reduced pressure, dissolved in 100 mL saturated $(NH_4)_2SO_4$, adjusted to ca. pH3 and extracted with EtOAc (3 x 100 mL). The combined extracts were dried over $MgSO_4$ and the solvent removed under reduced pressure.

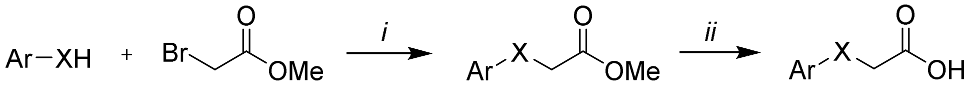

Scheme S1. Preparation of carboxylic acids. Reagents and conditions: (i) K_2CO_3, THF, reflux; (ii) LiOH, 3:1 $MeOH/H_2O$, rt.

3,5-Dimethoxyphenoxyacetic acid 4g

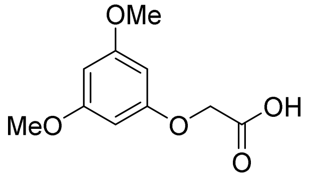

4g

Prepared from 3,5-dimethoxyphenol (3.01 g, 20 mmol), methyl bromoacetate (6.12 g, 40 mmol) and potassium carbonate (13.82 g, 100 mmol) in 100 mL THF. The resultant mixture was subjected to column chromatography on silica gel (eluent 50% CH_2Cl_2 in pentanes) to yield methyl (3,5-dimethoxyphenoxy)acetate as an off white solid (4.43g, 97%). ^{1}H NMR (300 MHz, $CDCl_3$, 294 K): δ = 3.75 (s, 6H, ArO-CH_3), 3.80 (s, 3H, OCH_3), 4.59 (s, 2H, ArOCH_2), 6.08 (m, 2H, Ar*H*), 6.11 (m, 1H. Ar*H*); ^{13}C NMR (75 MHz, $CDCl_3$, 296 K): δ = 52.7, 55.8, 65.6, 93.9, 94.3, 160.0, 162.0, 169.6. The ester (4.2 g, 18.6 mmol) was dissolved 135 mL CH_3OH and 45 mL H_2O in and subjected to hydrolysis by lithium hydroxide (9.48 g, 92.8 mmol) to yield **X** as an off-white solid (3.47 g, 88%). ^{1}H NMR (300 MHz, *d*6-acetone, 294 K): δ = 3.75 (s, 6H, ArO-CH_3), 4.13 (br-s, 1H, CO_2*H*), 4.65 (s, 2H, ArOCH_2), 6.11 (m, 3H, Ar*H*); ^{13}C NMR (75 MHz, $CDCl_3$, 295 K): δ = 55.6, 65.5, 94.2, 94.3, 161.0, 162.6, 170.2. Data consistent with literature.[1]

3,4,5-Trimethoxyphenoxyacetic acid 4h

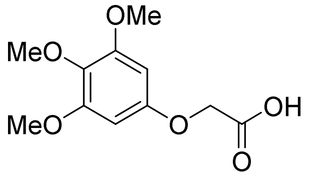

4h

Prepared from 3,4,5-trimethoxy phenol (3.68 g, 20 mmol), methyl bromoacetate (6.12 g, 40 mmol) and potassium carbonate (13.82 g, 100 mmol) in 100 mL THF. The resultant mixture was subjected to column chromatography on silica gel (eluent CH_2Cl_2) to yield methyl 3,4,5-trimethoxyphenoxyacetate as a colourless solid (3.27 g, 64%). ^{1}H NMR (300 MHz, $CDCl_3$, 294 K): δ = 3.70 (s, 3H, OCH_3), 3.74 (s, 3H, OCH_3), 3.75 (s, 6H, ArOCH_3), 4.53 (s, 2H, OCH_2), 6.10 (s, 2H, Ar*H*); ^{13}C NMR (75 MHz, $CDCl_3$, 295 K): δ = 52.6, 56.5, 61.3, 66.1, 92.9, 133.4, 154.1, 154.7, 169.7. The ester (2.86 g, 11.1 mmol) was dissolved 90 mL CH_3OH and 30 mL H_2O in and subjected to hydrolysis by lithium hydroxide (5.67 g, 55.5 mmol) to yield **X** as an off-white solid which was recrystallized from (1:5) acetone/cyclohexane (2.36 g, 88%). ^{1}H NMR (300 MHz, *d*6-acetone, 294 K): δ = 3.64 (s, 3H, ArOCH_3), 3.79 (s, 6H,

ArOCH_3), 4.52 (br-s, 1H, CO_2H), 4.66 (s, 2H, OCH_2), 6.27 (s, 2H, Ar*H*); ^{13}C NMR (75 MHz, $CDCl_3$, 295 K): δ = 56.4, 60.6, 65.8, 93.7, 133.8, 154.8, 155.6, 170.4. Data consistent with literature.[1]

4-Methoxyphenylthioacetic acid 4j

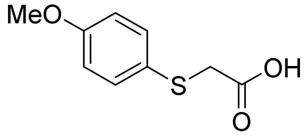

4j

Prepared from 4-methoxybenzenethiol (2.8 g, 20 mmol), methyl bromoacetate (6.12 g, 40 mmol) and potassium carbonate (13.8 g, 100 mmol) in 100 mL THF. The resultant mixture was subjected to column chromatography on silica gel (eluent 50% CH_2Cl_2 in pentanes) to yield methyl 4-methoxyphenylthioacetate as a colourless oil (3.64 g, 86%). ^{1}H NMR (300 MHz, $CDCl_3$, 294 K): δ = 3.51 (s, 2H, SCH_2), 3.68 (s, 3H, OCH_3), 3.78 (s, 3H, ArOCH_3), 6.83 (d, *J* = 8.9 Hz, 2H, Ar*H*), 7.40 (d, *J* = 8.9 Hz, 2H, Ar*H*); ^{13}C NMR (75 MHz, $CDCl_3$, 296 K): δ = 38.5, 52.4, 55.3, 114.7, 124.9, 134.2, 159.7, 170.4. The ester (3.0 g, 14.1 mmol) was dissolved 105 mL CH_3OH and 35 mL H_2O in and subjected to hydrolysis by lithium hydroxide (6.56 g, 71 mmol) to yield **X** as an off-white solid which was recrystallized from (1:10) acetone/cyclohexane (2.77 g, 99%). ^{1}H NMR (400 MHz, $CDCl_3$, 296 K): δ = 3.54 (s, 2H, SCH_2), 3.79 (s, 3H, ArOCH_3), 6.85 (d, *J* = 8.9 Hz, 2H, Ar*H*), 7.43 (d, *J* = 8.9 Hz, 2H, Ar*H*), 11.31 (br-s, 1H, CO_2H); ^{13}C NMR (75 MHz, $CDCl_3$, 295 K): δ = 38.6, 55.4, 114.8, 124.6, 134.3, 159.9, 176.3. Data consistent with literature.[2]

4-(Methylthio)phenoxyacetic acid 4k

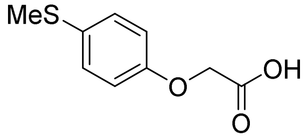

4k

Prepared from 4-(methylthio)phenol (2.80 g, 20 mmol), methyl bromoacetate (6.12 g, 40 mmol) and potassium carbonate (13.80 g, 100 mmol) in 100 mL THF. The resultant mixture was subjected to column chromatography on silica gel (eluent 50% CH_2Cl_2 in pentanes) to yield methyl 4-(methylthio)phenoxyacetate a colourless oil (3.07 g, 72%). ^{1}H NMR (400 MHz, $CDCl_3$, 294 K): δ = 2.43 (s, 3H, ArS-CH_3), 3.79 (s, 3H, CO_2-CH_3), 4.61 (s, 2H, ArO-CH_2), 6.85 (m, 2H, Ar*H*), 7.24 (m, 2H,

Ar*H*); ^{13}C NMR (75 MHz, $CDCl_3$, 295 K): δ = 18.0, 52.7, 65.9, 155.8, 130.1, 130.8, 156.6, 169.7. This ester (2.50 g, 11.8 mmol) was dissolved 90 mL CH_3OH and 30 mL H_2O in and subjected to hydrolysis by lithium hydroxide (5.47 g, 59 mmol) to yield **X** as an off-white solid which was recrystallized from acetone/cyclohexane (1:10) to yield colorless needles (2.01 g, 86%). M.p. 108 °C. ^{1}H NMR (400 MHz, $CDCl_3$, 296 K): δ = 2.45 (s, 3H, ArS-CH_3), 4.67 (s, 2H, ArO-CH_2), 6.86 (m, 2H, Ar*H*), 7.26 (m, 2H, Ar*H*), 9.54 (br-s, 1H, CO_2H); ^{13}C NMR (75 MHz, $CDCl_3$, 297 K): δ = 55.6, 65.5, 94.2, 94.3, 161.0, 162.6, 170.2; LR-ESIMS: m/z = 199 $[MH]^+$; HR-ESIMS: m/z = 199.0424 (calcd. for $C_9H_{11}O_3S$, 199.0429).

Benzyl (4-methoxyphenoxy)acetate 15

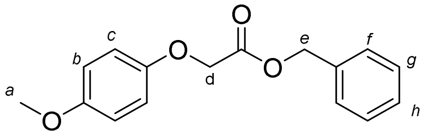

15

15 was prepared by stirring (4-methoxyphenoxy)acetic acid (182.2 mg, 1 mmol), benzyl alcohol (119 mg, 1.1 mmol), *N,N′*-dicyclohexylcarbodiimide (413 mg, 1.1 mmol) and 4-dimethylaminopyridine (12 mg, 0.1 mmol) in anhydrous CH_2Cl_2 (25 mL) overnight at room temperature in an argon atmosphere. The dicyclohexylurea biproduct was removed by filtration and the filtrate washed with water (3 x 30 mL) 5% acetic acid solution (3 x 30 mL), again with water (3 x 30 mL) and dried over anhydrous $MgSO_4$. The solvent was removed under reduced pressure and the crude reaction mixture purified by column chromatography on silica gel (eluent: CH_2Cl_2) to yield **15** as a clear oil (160.7 mg, 59%). ^{1}H NMR (400 MHz, $CDCl_3$, 300 K): δ = 3.77 (s, 3H, H_a), 4.62 (s, 2H, H_d), 5.24 (s, 2H, H_e), 6.80-6.87 (m, 4H, $H_{b,c}$), 7.33-7.37 (m, 5H, $H_{f,g,h}$); ^{13}C NMR (100 MHz, $CDCl_3$, 300 K): δ = 55.7, 66.4, 67.0, 114.7, 116.0, 128.5, 128.5, 128.7, 135.2, 152.0, 154.6, 169.1; LR-ESIMS: *m/z* = 290 [M*NH$_4$*]$^+$; HR-ESIMS: *m/z* = 290.1390 (calcd. for $C_{16}H_{20}NO_4$, 290.1387).

Dibenzyl 2,2'-(1,4-phenylenebis(oxy))diacetate 18

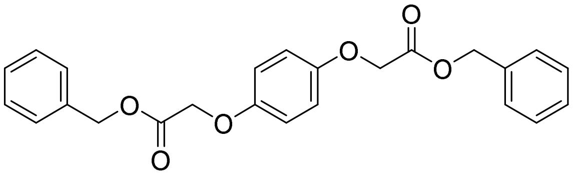

18

Hydroquinone-O,O’-dicacetic acid (452.4 mg, 2.0 mmol) and benzyl alcohol (0.19 mL, 2.2 mmol) were dissolved in 20 mL anhydrous DMF. This mixture was cooled to 0°C and to it was added 1-Ethyl-3-(3-dimethylaminopropyl)carbodiimide (0.39 ,L, 2.2 mmol) and then DMAP (24.4 mg, 0.2 mmol). The mixture was then allowed to warm to room temperature and stirred overnight. The mixture was then concentrated, the resultant residue taken up in 50 mL EtOAc, washed with H_2O (3 x 100 mL) and dried over $MgSO_4$. The solvent was removed under reduced pressure to yield **18** as a clear oil (118.5 mg, 15%). ^{1}H NMR (500 MHz, *d6*-acetone, 295 K): δ = 4.73 (s, 4H, ArOCH_2), 5.22 (s, 4H, CO_2CH_2Ph), 6.88 (s, 4H, Ar*H*), 7.35-7.40 (m, 10H, Ar*H*); ^{13}C NMR (75 MHz, *d6*-acetone, 297 K): δ = 66.5, 67.0, 116.5, 128.9, 129.0, 129.4, 137.0, 153.8, 169.6; LR-ESIMS: *m/z* = 424 [M*NH$_4$*]$^+$; HR-ESIMS: *m/z* = 424.1755 (calcd. for $C_{24}H_{26}NO_6$, 424.1760).

Optimisation Studies

The reaction between 4-methoxyphenoxyacetic acid **4d** and maleimide **1a** was used as a test reaction to determine the optimum reaction conditions. Early work indicated that the reaction generally needed to be run overnight. However, for the purposes of optimisation a reaction time of 5 hours was adopted.

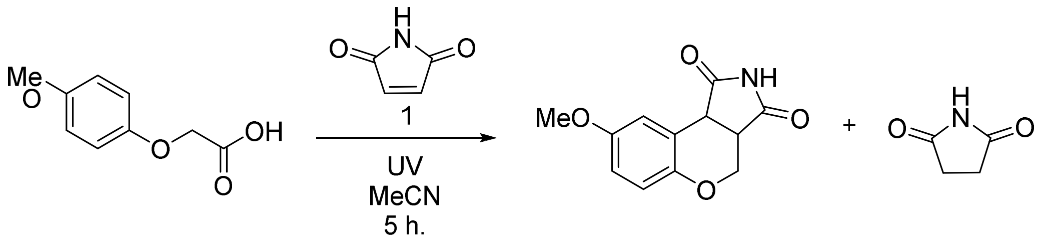

Scheme S2. Test reaction for optimisation studies.

Reaction Stoichiometry

4-Methoxyphenoxyacetic acid **4d** (47.4 mg, 0.26 mmol) was dissolved in 10 mL anhydrous MeCN along with various equivalents of maleimide **1**. The resultant mixture was degassed for 15 minutes by argon bubbling before being irradiated for 5 hours by two hemispherical banks of six Philips Cleo 15W tubes. The solvent was then removed under reduced pressure and the concentration or reagents and products were determined by ^{1}H NMR in *d6*-acetone with reference to CH_2Br_2 internal standard. This data is sumarised in the following table:

**4d (mol eq.)**	**1a (mol eq.)**	**% Conversion**	**% Yield 5d**	**% Yield 7**
1	1	28	22	13
1	2	51	33	9
1	4	66	41	5
1	10	- [a]	-	-

[a] Concentrations of reagents and products could not be determined as the sample repeatedly failed to shim.

Reaction Medium

4-Methoxyphenoxyacetic acid **4d** (47.4 mg, 0.26 mmol) and maleimide **1** (101.6 mg, 1.04 mmol) were dissolved in a variety solvents. The resultant mixtures were degassed for 15 minutes by argon bubbling before being irradiated for 5 hours by two hemispherical banks of six Philips Cleo 15W tubes. A 2 mL aliquot if the reaction mixture was taken at this time, the solvent was then removed under reduced pressure and the concentration or reagents and products were determined by ^{1}H NMR in *d6*-acetone with reference to CH_2Br_2 internal standard. This data is summarised in the following table:

Solvent	% Conversion	%Yield 5d	% Yield 7
MeCN [a]	66%	41%	5%
MeOH [a]	60%	40%	5%
t-BuOH [b]	44%	37%	5%
Acetone [c]	15%	12%	<1%
DMF [d]	-	-	-
DCM [a]	-	-	-
Benzene [b]	-	-	-
H_2O	-	-	-
35% H_2O/MeCN [b]	84%	61%	5%

[a] anhydrous, freshly distilled [b] used straight from bottle [c] dried o/n with 4Å mol. sieves [d] purchased anhydrous.

Variation of Maleate

8-Methoxy-2-methyl-3a,4-dihydrochromeno[3,4-*c*]pyrrole-1,3(2*H*,9b*H*)-dione.

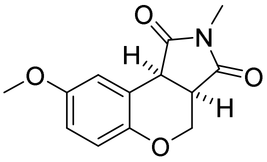

N-Methylmaleimide (417 mg, 3.75 mmol) and 4-methoxyphenoxyacetic acid (137mg, 0.75 mmol) were dissolved in 13 mL CH_3CN and 7 mL H_2O and irradiated though pyrex for 18 hours. Following irradiation a 0.5 mL aliquot was taken from the reaction mixture and the solvent was removed under reduced pressure. ^{1}H NMR analysis (w.r.t. CH_2Br_2 standard) of the resultant mixture revealed: 8-methoxy-2-methyl-3a,4-dihydrochromeno[3,4-*c*]pyrrole-1,3(2*H*,9b*H*)-dione (0.19 mmol, 25%), and unreacted **4d** (0.53 mmol, 70%).

Attempted synthesis of 8-methoxy-2-phenyl-3a,4-dihydrochromeno[3,4-*c*]pyrrole-1,3(2*H*,9b*H*)-dione.

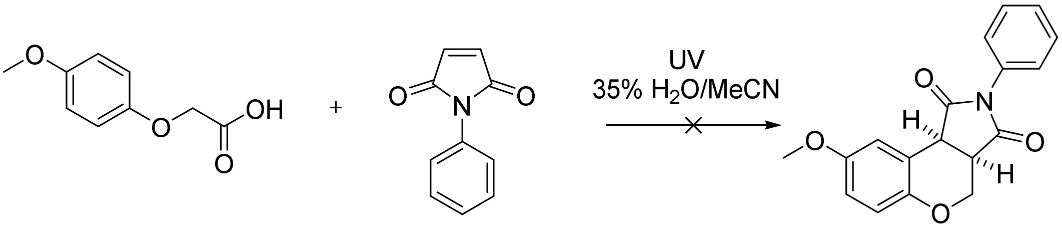

N-phenylmaleimide (1299 mg, 7.5 mmol) and 4-methoxyphenoxyacetic acid (137mg, 0.75 mmol) were dissolved in 13 mL CH_3CN and 7 mL H_2O and irradiated though pyrex for 18 hours. Following irradiation a 0.5 mL aliquot was taken from the reaction mixture and the solvent was removed under reduced pressure. ^{1}H NMR analysis revealed only starting materials present.

Attempted synthesis of 8-methoxy-3a,4-dihydro-1H-furo[3,4-c]chromene-1,3(9bH)-dione

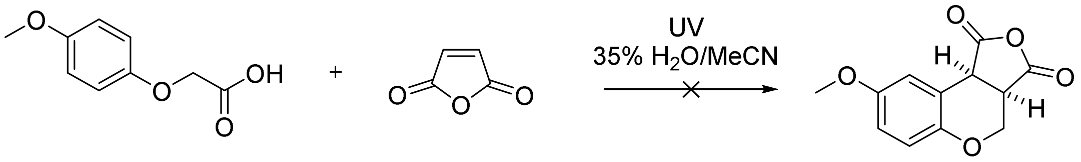

Maleic anhydride (736 mg, 7.5 mmol) and 4-methoxyphenoxyacetic acid (137mg, 0.75 mmol) were dissolved in 13 mL CH_3CN and 7 mL H_2O and irradiated though pyrex for 18 hours. Following irradiation a 0.5 mL aliquot was taken from the reaction mixture and the solvent was removed under reduced pressure. ^1H NMR analysis revealed only starting materials present.

Based on this work it was decided to use a five-fold excess of **1a** with a variety of acids **4** in 35% H_2O/MeCN as the optimum reaction conditions.

General Procedure for Preparative Irradiations

Known amounts of desired reagents were dissolved in a mixture of 35 : 65 H_2O in MeCN. The resulting mixture was degassed by bubbling with argon for 20 minutes. The mixture was then irradiated with twelve 29 cm 15 W Philips Cleo tubes (λ = 350 nm) arranged in two hemispherical banks of six for the desired reaction time. Following irradiation the solvent was removed under reduced pressure and the crude reaction mixture was purified if necessary. All reagents were commercially available, MeCN was used as purchased and H_2O was deionised.

3a,4-Dihydrochromeno[3,4-*c*]pyrrole-1,3(2*H*,9b*H*)-dione 5a (Table 1, Entry 1).

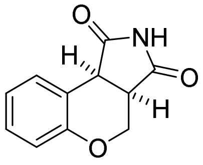

5a

Maleimide (366 mg, 3.75 mmol) and phenoxyacetic acid (114 mg, 0.75 mmol) were dissolved in 13 mL CH_3CN and 7 mL H_2O and irradiated though Pyrex for 18 hours. Following irradiation the reaction mixture was concentrated under reduced pressure. ^{1}H NMR analysis revealed that the desired product **5a** had not been formed.

8-Methyl-3a,4-dihydrochromeno[3,4-*c*]pyrrole-1,3(2*H*,9b*H*)-dione 5b (Table 1, Entry 2)

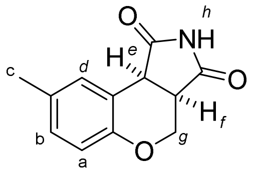

5b

Maleimide (732 mg, 7.5 mmol) and 4-methylphenoxyacetic acid (124.7 mg, 0.75 mmol) were dissolved in 13 mL CH_3CN and 7 mL H_2O and irradiated though pyrex for 25 hours. Following irradiation the reaction mixture was concentrated under reduced pressure and the crude mixture was purified by column chromatography on silica gel (eluent: 10% EtOAc in CH_2Cl_2) to yield **5b** as a colourless solid (46.6 mg, 29%). M.p. 172 °C. ^{1}H NMR (400 MHz, *d6*-acetone, 295 K): δ = 2.27 (s, 3H, H_c), 3.59 (m, 1H, H_f), 3.93 (dd, J = 3.9, 11.3 Hz, 1H, H_g), 4.12 (d, J = 9.2 Hz, 1H, H_e), 4.51 (dd, J = 2.6, 11.2 Hz, 1H, $H_{g'}$), 6.74 (d, J = 8.3 Hz, 1H, H_a), 7.00 (d, J = Hz, 1H, H_b), 7.30 (s, 1H, H_d), 10.15 (br-s, 1H, H_h); ^{13}C NMR (75 MHz,

d6-acetone, 295 K): δ = 20.7, 41.9, 44.3, 64.8, 118.0, 119.4, 131.3, 132.1, 136.0, 154.2, 177.6, 178.5; LR-ESIMS: *m/z* = 218 [M*H*]$^+$; HR-ESIMS: *m/z* = 218.0814 (calcd. for $C_{12}H_{12}NO_3$, 218.0817).

8-(*tert*-Butyl)-3a,4-dihydrochromeno[3,4-c]pyrrole-1,3(2*H*,9b*H*)-dione 5c (Table 1, Entry 3)

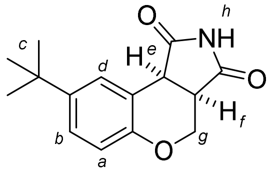

5c

Maleimide (732 mg, 7.5 mmol) and 4-tert-butylphenoxyacetic acid (156 mg, 0.75 mmol) were dissolved in 13 mL CH_3CN and 7 mL H_2O and irradiated though pyrex for 18 hours. Following irradiation the reaction mixture was concentrated under reduced pressure and the crude mixture was purified by column chromatography on silica gel (eluent: 5-10% acetone in CH_2Cl_2) to yield **5b** as a colourless solid (104 mg, 54%). Mp 174 °C. ^{1}H NMR (400 MHz, *d6*-acetone, 295 K): δ = 1.30 (s, 9H, H_c), 3.58-3.62 (m, 1H, H_f), 3.95 (dd, *J* = 4.0, 11.3 Hz, 1H, H_g), 4.16 (d, *J* = 9.2 Hz, 1H, H_e), 4.51 (dd, *J* = 2.7, 11.3 Hz, 1H, $H_{g'}$), 6.78 (d, *J* = 8.5 Hz, 1H, H_a), 7.25 (dd, *J* = 2.5, 8.6 Hz, 1H, H_b), 7.55 (d, *J* = 2.5 Hz, 1H, H_d), 10.15 (br-s, 1H, H_h); ^{13}C NMR (75 MHz, *d6*-acetone, 297 K): δ = 31.8, 34.8, 42.0, 44.3, 64.7, 117.7, 118.9, 126.4, 127.8, 145.6, 154.1, 177.7, 178.5 ; LR-ESIMS: *m/z* = 282 [M*Na*]$^+$; HR-ESIMS: *m/z* = 282.1103 (calcd. for $C_{15}H_{17}NO_3Na$, 282.1106).

8-Methoxy-3a,4-dihydrochromeno[3,4-*c*]pyrrole-1,3(2*H*,9b*H*)-dione 5d (Table 1, Entry 4)

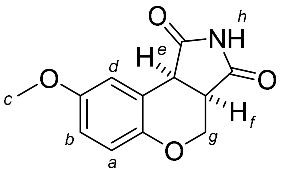

5d

Maleimide (366 mg, 3.75 mmol) and 4-(methoxy)phenoxyacetic acid (137 mg, 0.75 mmol) were dissolved in 13 mL CH_3CN and 7 mL H_2O and irradiated though pyrex for 18 hours. Following irradiation the reaction mixture was concentrated under reduced pressure and the crude mixture was purified by column chromatography on silica gel (eluent: 10% EtOAc in CH_2Cl_2) to yield **5d** as an off-white solid (93.9 mg, 54%). M.p. 217 °C. ^{1}H NMR (300 MHz, *d6*-acetone, 295 K): δ = 3.55-3.60 (m, 1H, H_f), 3.88 (s, 3H, H_c), 3.91 (dd, *J* = 4.0, 11.2 Hz, 1H, H_g), 4.16 (d, *J* = 9.4 Hz, 1H, H_e), 4.50 (dd, *J* =

2.5, 11.2 Hz, 1H, H_g), 6.79 (m, 2H, Ar-H), 7.06 (m, 1H, Ar-H), 10.18 (br-s, 1H, H_h); ^{13}C NMR (75 MHz, *d6*-acetone, 295 K): δ = 42.2, 44.3, 56.0, 65.0, 115.3, 115.4, 118.9, 120.4, 150.3, 155.6, 177.5, 178.6; LR-EIMS: *m/z* = 233 $[M]^+$; HR-ESIMS: *m/z* = 233.0680 (calcd. for $C_{12}H_{11}NO_4$, 233.0683).

7-Methoxy-3a,4-dihydrochromeno[3,4-*c*]pyrrole-1,3(2*H*,9b*H*)-dione 5e & 9-methoxy-3a,4-dihydrochromeno[3,4-*c*]pyrrole-1,3(2*H*,9b*H*)-dione 5e' (Table 1, Entry 5)

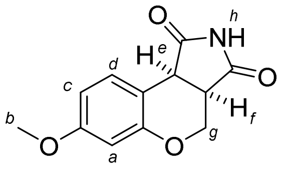

5e

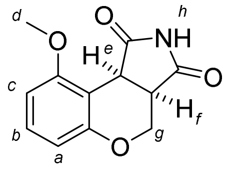

5e'

Maleimide (366 mg, 3.75 mmol) and 3-(methoxy)phenoxyacetic acid (137 mg, 0.75 mmol) were dissolved in 13 mL CH_3CN and 7 mL H_2O and irradiated though pyrex for 18 hours. Following irradiation the reaction mixture was concentrated under reduced pressure and the crude mixture was purified by column chromatography on silica gel (eluent: 5% EtOAc in CH_2Cl_2) to yield **5e** as an off-white solid (111 mg, 64%). M.p. 156 °C. ^1H NMR (300 MHz, *d6*-acetone, 295 K): δ = 3.55-3.60 (m, 1H, H_f), 3.74 (s, 3H, H_b), 3.95 (dd, *J* = 4.0, 11.4 Hz, 1H, H_g), 4.08 (d, *J* = 9.2 Hz, 1H, H_e), 4.53 (dd, *J* = 2.7, 11.3 Hz, 1H, $H_{g'}$), 6.42 (d, *J* = 2.6 Hz, 1H, H_a), 6.61 (dd, *J* = 2.8, 8.7 Hz, 1H, H_c), 7.39 (d, *J* = 8.3 Hz, 1H, H_d), 10.14 (br-s, 1H, H_h); ^{13}C NMR (75 MHz, *d6*-acetone, 296 K): δ = 41.2, 44.1, 55.7, 64.7, 103.1, 109.9, 111.5, 131.7, 157.2, 161.0, 178.1, 178.6; LR-ESIMS: *m/z* = 234 $[MH]^+$; HR-ESIMS: *m/z* = 234.0765 (calcd. for $C_{12}H_{12}NO_4$, 234.0766). As above to yield **5e'** as a yellow oil (28.2 mg, 16%). ^1H NMR (300 MHz, *d6*-acetone, 294 K): δ = 3.52-3.57 (m, 1h, H_f), 3.79 (dd, *J* = 4.3, 11.2 Hz, 1H, H_g), 3.87 (s, 3H, H_d), 4.54 (dd, *J* = 1.8, 11.2 Hz, 1H, $H_{g'}$), 4.60 (d, *J* = 9.7 Hz, 1H, H_e), 6.50 (d, *J* = 8.2 Hz, 1H, *Ar*-H), 6.71 (d, *J* = 8.3 Hz, 1H, *Ar*-H), 7.15 (t, *J* = 8.2 Hz, 1H, *Ar*-H), 10.14 (br-s, 1H, H_h); ^{13}C NMR (75 MHz, *d6*-acetone, 294 K): δ = 37.5, 45.0, 56.4, 66.7, 106.3, 110.9, 139.4, 158.5, 160.1, 176.7, 179.0; LR-EIMS: *m/z* = 234 $[MH]^+$; HR-ESIMS *m/z* = 234.0763 (calcd. for $C_{12}H_{12}NO_4$, 234.0766).

3a,4-Dihydrochromeno[3,4-*c*]pyrrole-1,3(2*H*,9b*H*)-dione 5a (Table 1, Entry 6)

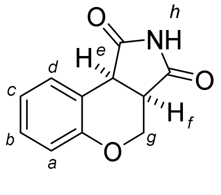

5a

Maleimide (366 mg, 3.75 mmol) and 2-(methoxy)phenoxyacetic acid (137 mg, 0.75 mmol) were dissolved in 13 mL CH_3CN and 7 mL H_2O and irradiated though pyrex for 18 hours. Following irradiation the reaction mixture was concentrated under reduced pressure and the crude mixture was purified by column chromatography on silica gel (eluent: 10% EtOAc in CH_2Cl_2) to yield **5a** as a colourless solid (81.5 mg, 54%). ^{1}H NMR (300 MHz, *d6*-acetone, 295 K): δ = 3.61-3.67 (m, 1H, H_f), 4.00 (dd, *J* = 4.2, 11.5 Hz 1H, H_g), 4.19 (d, *J* = 9.2 Hz, 1H, H_e), 4.55 (dd, *J* = 3.1, 11.4 Hz 1H, $H_{g'}$), 6.87 (d, *J* = 8.2 Hz, 1H, H_d), 7.03 (t, *J* = 8.2 Hz, 1H, H_c), 7.22 (t, *J* = 8.0 Hz, 1H, H_b), 7.51 (d, *J* = 7.8 Hz, 1H, H_a), 10.18 (br-s, 1H, H_h). Consistent with literature.[3]

7,9-Dimethoxy-3a,4-dihydrochromeno[3,4-*c*]pyrrole-1,3(2*H*,9b*H*)-dione 5g (Table 1, Entry 7)

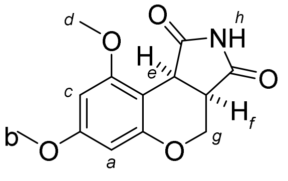

5g

Maleimide (366 mg, 3.75 mmol) and 3,5-dimethoxyphenoxyacetic acid (159.2 mg, 0.75 mmol) were dissolved in 13 mL CH_3CN and 7 mL H_2O and irradiated though pyrex for 18 hours. Following irradiation the reaction mixture was concentrated under reduced pressure and the crude mixture was purified by column chromatography on silica gel (eluent: 10-20% EtOAc in CH_2Cl_2) to yield **5g** as a colourless solid (161 mg, 82%). M.p. 236 °C. ^{1}H NMR (300 MHz, *d6*-acetone, 296 K): δ = 3.52-3.58 (m, 1H, H_f), 3.79 (s, 3H, OC*H*3), 3.82 (dd, *J* = 4.3, 11.2 Hz, 1H, H_g), 3.89 (s, 3H, OC*H*3), 4.50 (d, *J* = 9.7 Hz, 1H, H_e), 4.55 (dd, *J* = 1.8, 11.2 Hz, 1H, $H_{g,}$), 6.12 (d, *J* = 2.3 Hz, 1H, Ar*H*), 6.32 (d, *J* = 2.3 Hz, 1H, Ar*H*), 10.14 (br-s, 1H, H_h); ^{13}C NMR (75 MHz, *d6*-DMSO, 297 K): δ = 35.9, 43.5, 55.2, 55.8, 65.7, 93.6, 94.7, 101.6, 157.6, 159.2, 177.2, 179.2; LR-ESIMS: *m/z* = 264 [M*H*]$^+$; HR-ESIMS: *m/z* = 246.0869 (calcd. for $C_{13}H_{14}NO_5$, 264.0872).

7,8,9-Trimethoxy-3a,4-dihydrochromeno[3,4-*c*]pyrrole-1,3(2*H*,9b*H*)-dione 5h (Table 1, Entry 8)

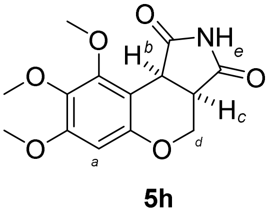

5h

Maleimide (366 mg, 3.75 mmol) and 3,4,5-trimethoxyphenoxyacetic (181.7 mg, 0.75 mmol) were dissolved in 13 mL CH_3CN and 7 mL H_2O and irradiated though pyrex for 64 hours. Following

irradiation the reaction mixture was concentrated under reduced pressure, diluted with 100 mL CH_2Cl_2, washed with saturated aqueous $NaHCO_3$ (100 mL), deionised water (2 x 100 mL), dried over anhydrous $MgSO_4$ and the solvent evaporated to yield **5h** as a yellow solid (52.2 mg, 24%). Mp 165 °C. ^{1}H NMR (400 MHz, *d6*-acetone, 295 K): δ = 3.48-3.52 (m, 1H, H_c), 3.74 (dd-overlapped, *J* = 4.4, 11.2 Hz, 1H, H_d), 3.75 (s-overlapped, 3H, Ar-OCH_3), 3.79 (s, 3H, Ar-OCH_3), 4.04 (s, 3H, Ar-OCH_3), 4.49 (d-overlapped, *J* = 9.7 Hz, 1H, H_b), 4.50 (dd-overlapped, *J* = 1.7, 11.2 Hz, 1H, $H_{d'}$), 6.31 (s, 1H, H_a), 10.21 (br-s, 1H. H_e); ^{13}C NMR (75 MHz, *d6*-acetone, 297 K): δ = 37.7, 44.9, 56.3, 60.7, 61.7, 67.0, 98.1, 107.1, 136.0, 138.6, 153.7, 154.6, 177.4, 179.2; LR-ESIMS: *m/z* = 294 [M*H*]$^+$; HR-ESIMS: *m/z* = 294.0975 (calcd. for $C_{14}H_{16}NO_6$, 294.0978). Analysis of the reaction mixture by ^{1}H NMR prior to chromatography revealed 71% unreacted 3,4,5-trimethoxyphenoxyacetic (0.53 mmol w.r.t. **5h**) indicating that the reaction had achieved only 29% conversion.

3a,4-Dihydrothiochromeno[3,4-*c*]pyrrole-1,3(2*H*,9b*H*)-dione 5i (Table 1, Entry 9)

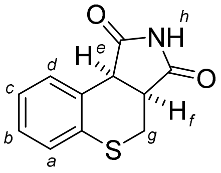

5i

Maleimide (366 mg, 3.75 mmol) and phenylthioacetic acid (126 mg, 0.75 mmol) were dissolved in 13 mL CH_3CN and 7 mL H_2O and irradiated though pyrex for 18 hours. Following irradiation the reaction mixture was concentrated under reduced pressure and the crude mixture was purified by column chromatography on silica gel (eluent: 7.5% EtOAc in CH_2Cl_2) to yield **5i** as a colourless solid (62.9 mg, 39%). Mp 157 °C. ^{1}H NMR (300 MHz, *d6*-acetone, 295 K): δ = 2.91 (dd, *J* = 4.9, 13.3 Hz, 1H, H_g), 3.26 (dd, *J* = 2.4, 13.3 Hz, 1H, $H_{g'}$), 3.82-3.88 (m, 1H, H_f), 4.35 (d, *J* = 9.5 Hz, 1H, H_e), 7.20-7.30 (m, 3H, Ar*H*), 7.43-7.49 (m, 1H. Ar*H*),10.37 (br-s, 1H, H_h).; ^{13}C NMR (75 MHz, *d6*-acetone, 295 K): δ = 30.1, 46.4, 47.2, 127.3, 128.4, 129.9, 132.5, 133.1, 135.4, 177.3, 179.3; LR-ESIMS: *m/z* = 237 [M*NH$_4$*]$^+$; HR-ESIMS: *m/z* = 237.0696 (calcd. for $C_{11}H_{13}N_2O_2S$, 237.0698).

8-Methoxy-3a,4-dihydrothiochromeno[3,4-c]pyrrole-1,3(2*H*,9b*H*)-dione 5j (Table 1, Entry 10)

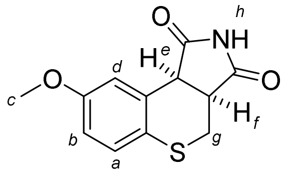

5j

Maleimide (366 mg, 3.75 mmol) and 4-methoxyphenyltioacetic acid (148.6 mg, 0.75 mmol) were dissolved in 13 mL CH_3CN and 7 mL H_2O and irradiated though pyrex for 72 hours. Following irradiation the reaction mixture was concentrated under reduced pressure and the crude mixture was purified by column chromatography on silica gel (eluent: 10-20% EtOAc in CH_2Cl_2) to yield **5j** as a yellow oil (67.5 mg, 36%). ^{1}H NMR (300 MHz, *d6*-acetone, 296 K): $\delta = 2.85$ (dd, $J = 4.9, 13.3$ Hz, 1H, H_g), 3.23 (dd, $J = 2.3, 13.3$ Hz, 1H, $H_{g'}$), 3.77-3.83 (m, overlapped, 1H, H_f), 3.81 (s, overlapped, 3H, H_c), 4.31 (d, $J = 9.4$ Hz, 1H, H_e), 6.84 (dd, $J = 2.8, 8.7$ Hz, 1H, H_b), 7.05 (d, $J = 2.7$ Hz, 1H, H_d), 7.20 (d, $J = 8.6$ Hz, 1H, H_a), 10.37 (br-s, 1H, H_h) ;^{13}C NMR (75 MHz, *d6*-acetone, 297 K): $\delta = 30.7$, 46.2, 47.6, 55.8, 114.2, 118.8, 126.1, 130.9, 133.8, 159.4, 177.2, 179.4; LR-ESIMS: *m/z* = 250 [M*H*]$^+$; HR-ESIMS: *m/z* = 250.0535 (calcd. for $C_{12}H_{12}NO_3S$, 250.0538).

8-(Methylthio)-3a,4-dihydrochromeno[3,4-c]pyrrole-1,3(2*H*,9b*H*)-dione 5k (Table 1, Entry 11)

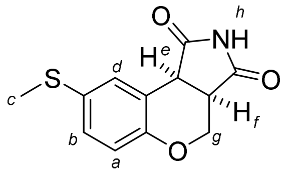

5k

Maleimide (366 mg, 3.75 mmol) and 4-methylthiophenoxyacetic acid (148.6 mg, 0.75 mmol) were dissolved in 13 mL CH_3CN and 7 mL H_2O and irradiated though pyrex for 72 hours. Following irradiation the reaction mixture was concentrated under reduced pressure and the crude mixture was purified by column chromatography on silica gel (eluent: 10% EtOAc in CH_2Cl_2) to yield **5k** as a off-white solid (27.3 mg, 14%). M.p. 160 °C. ^{1}H NMR (400 MHz, *d6*-acetone, 300 K): $\delta = 2.46$ (s, 3H, H_c), 3.61-3.64 (m, 1H, H_f), 3.98 (dd, $J = 4.1, 11.3$ Hz, 1H, H_g), 4.18 (d, $J = 9.2$ Hz, 1H, H_e), 4.53 (dd, $J = 2.8, 11.3$ Hz, 1H, H_g), 6.84 (d, $J = 8.5$ Hz, 1H, H_a), 7.16 (dd, $J = 2.3, 8.5$ Hz, 1H, H_b), 7.47 (d, $J = 2.3$ Hz, 1H, H_d), 10.16 (br-s, 1H, H_h); ^{13}C NMR (100 MHz, $CDCl_3$, 296 K): $\delta = 17.2$, 40.8, 43.2, 63.8, 117.7, 118.5, 128.9, 129.0, 131.9, 153.4, 175.7, 176.6; LR-ESIMS: *m/z* = 272 [M*Na*]$^+$; HR-ESIMS: *m/z* = 272.0354 (calcd. for $C_{12}H_{11}NO_3SNa$, 272.0357). Analysis of the reaction mixture by ^{1}H NMR prior to

chromatography revealed 47% unreacted 4-methylthiophenoxyacetic acid (0.35 mmol w.r.t. **5k**) indicating that the reaction had achieved only 53% conversion.

3a,4,5,9b-Tetrahydro-1*H*-pyrrolo[3,4-*c*]quinoline-1,3(2*H*)-dione 5l (Table 1, Entry 12)

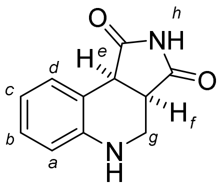

5l

Maleimide (366 mg, 3.75 mmol) and *N*-phenylglycine (113.4 mg, 0.75 mmol) were dissolved in 13 mL CH_3CN and 7 mL H_2O and irradiated though pyrex for 18 hours. Following irradiation the reaction mixture was concentrated under reduced pressure and the crude mixture was purified by column chromatography on silica gel (eluent: 20% EtOAc in CH_2Cl_2) to yield **5l** as an off-white solid (135 mg, 89%). M.p. 173-176 °C. ^{1}H NMR (500 MHz, *d6*-acetone, 293 K): δ = 3.12 (dd, *J* = 4.4, 11.3 Hz, 1H, H_g), 3.47-3.51 (m, 1H, H_f), 3.54 (dt, *J* = 3.0, 11.3 Hz, 1H, H_g), 4.04 (d, *J* = 9.3 Hz, 1H, H_e), 5.00 (br-s, 1H, H_h), 6.68 (d, *J* = 7.9 Hz, 1H, Ar*H*), 6.72 (t, *J* = 7.5 Hz, 1H, Ar*H*), 7.01 (t, *J* = 7.4 Hz, 1H, Ar*H*), 7.36 (d, *J* = 7.6 Hz, 1H, Ar*H*), 10.04 (br-s, 1H, H_i); ^{13}C NMR (75 MHz, *d6*-acetone, 298 K): δ = 41.9, 43.5, 45.2, 116.2, 118.5, 119.5, 128.5, 131.1, 148.1, 178.2, 179.9; LR-ESIMS: *m/z* = 203 [M*H*]$^+$; HR-ESIMS: *m/z* = 203.0815 (calcd. for $C_{11}H_{10}N_2O_2$, 203.0821).

8-Chloro-3a,4-dihydrochromeno[3,4-*c*]pyrrole-1,3(2*H*,9b*H*)-dione 5m (Table 1, Entry 1).

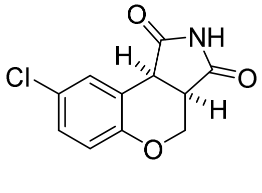

Maleimide (366 mg, 3.75 mmol) and 4-chlorophenoxyacetic acid (140 mg, 0.75 mmol) were dissolved in 13 mL CH_3CN and 7 mL H_2O and irradiated though pyrex for 18 hours. Following irradiation the

reaction mixture was concentrated under reduced pressure. ^{1}H NMR analysis revealed peaks that could be attributed to the desired product **5m** (0.025 mmol; 3%).

Attempted Synthesis of 9

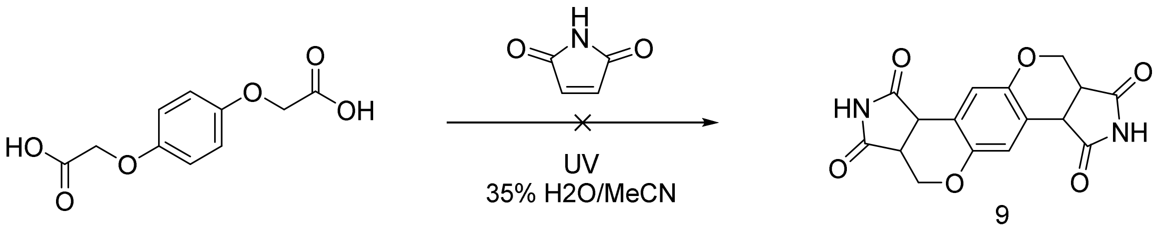

Maleimide (732 mg, 7.5 mmol) and hydroquinone-O,O'-diacetic acid (169.7 mg, 0.75 mmol) were dissolved in 26 mL CH_3CN and 7 mL H_2O and irradiated though pyrex for 18 hours. Following irradiation the reaction mixture was concentrated under reduced pressure. ^{1}H NMR and GC-MS analyses revealed that the desired product **9** had not been formed.

X-ray Crystallography

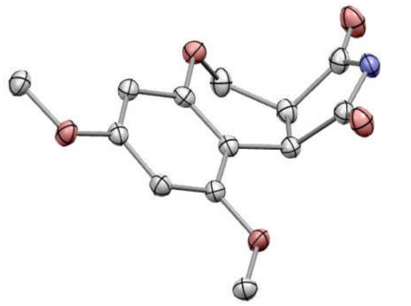

Fig. S1. The X-ray crystal structure of **5g**.

Table S1. Crystal data & structure refinement for **5g**.

Identification code	**5g**
CCDC code	962517
Empirical Formula	$C_{13}H_{13}NO_5$
Formula Weight	263.25
Crystal Colour, Habit	colourless prism
Crystal Dimensions	0.200 mm x 0.100 mm x 0.030 mm
Crystal System	monoclinic
Lattice Type	Primitive
Lattice Parameters	*a* = 7.925(4) Å *β* = 92.336(8)°<br>*b* = 8.466(3) Å<br>*c* = 17.187(6) Å
Volume	1152.2(8) $Å^3$
Space Group	$P2_1$/n(#14)
Z value	4
Density (calculated)	1.517 g/mL
F(000)	552.00
μ(CuKα)	9.993 cm^{-1}
Diffractometer	Saturn70
Radiation Voltage	CuKα (λ = 1.54187 Å)
Current Temperature	173 K
Detector Aperture	70 x 70 mm
Pixel Size	0.034 nm
2θmax	136.7°
No. of Reflections Measured	Total: 15,295, Unique: 2084 (R_{int} = 0.0431)
Corrections	Lorentz-polarization
Structure Solution	Direct Methods
Refinement	Full-matrix least-squares on F^2
Function Minimized	Σ w $(Fo^2 - Fc^2)^2$
Least Squares Weights	w = $1/[\sigma^2(Fo^2) + (0.1051P)^2 + 2.9953P]$<br>where P = $(Max(Fo^2, 0) + 2Fc^2)/3$
2θmax cutoff	136.7°
Anomalous Dispersion	All non-hydrogen atoms
No. Observations	(All reflections) 2084
No. Variables	176
Reflection/Parameter Ratio	11.9
Residuals: R1 (I>2.00σ(I))	0.0361
Residuals: R (All reflections)	0.0389

Residuals: wR2 (All reflections)	0.1115
Goodness of Fit	0.956
Indicator Max Shift/Error in Final Cycle	0.000
Maximum peak in Final Diff. Map	0.22 e /Å^3
Minimum peak in Final Diff. Map	-0.20 e /Å^3

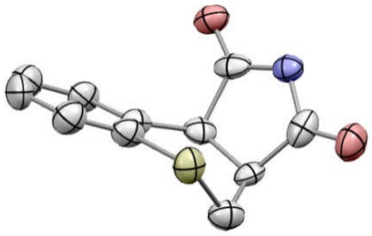

Fig. S2. The X-ray crystal structure of **5i**.

Table S2. Crystal data & structure refinement for **5i**.

Identification code	**5i**
CCDC code	962518
Empirical Formula	$C_{11}H_9NO_2S$
Formula Weight	219.26
Crystal Colour, Habit	colourless prism
Crystal Dimensions	0.100 mm x 0.060 mm x 0.030 mm
Crystal System	monoclinic
Lattice Type	C-centered
Lattice Parameters	a = 12.590(7) Å β = 96.690(12)°
	b = 11.384(5) Å
	c = 27.890(13) Å
Volume	3970(3) Å^3
Space Group	Cc(#9)
Z value	16
Density (calculated)	1.467 g/mL
F(000)	1824.00
μ(CuKα)	3.016 cm^{-1}
Diffractometer	Mercury70
Radiation Voltage	MoKα (λ = 0.71075)
Current Temperature	93 K
Detector Aperture	70 x 70 mm
Pixel Size	0.068 mm
2θmax	50.8°
No. of Reflections Measured	Total: 10656, Unique: 5199 (R_{int} = 0.0744)
Corrections	Lorentz-polarization
Structure Solution	Charge Flipping (Superflip)
Refinement	Full-matrix least-squares on F^2
Function Minimized	Σ w $(Fo^2 - Fc^2)^2$
Least Squares Weights	w = $1/[\sigma^2(Fo^2) + (0.1051P)^2 + 2.9953P]$
	where P = $(Max(Fo^2, 0) + 2Fc^2)/3$
2θmax cutoff	50.8°
Anomalous Dispersion	All non-hydrogen atoms
No. Observations	(All reflections) 5199
No. Variables	557

Reflection/Parameter Ratio	9.33
Residuals: R1 (I>2.00σ(I))	0.0738
Residuals: R (All reflections)	0.0979
Residuals: wR2 (All reflections)	0.1927
Goodness of Fit	1.073
Indicator Max Shift/Error in Final Cycle	0.000
Maximum peak in Final Diff. Map	0.52 e /Å^3
Minimum peak in Final Diff. Map	-0.43 e /Å^3

Table S3. Coupling Constant between Protons at Ring Junction

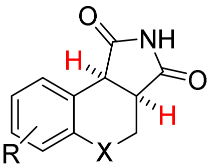

Entry	Product		$^{3}J_{H\text{-}H}$ (Hz)
1	**4a**	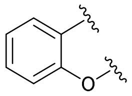	-
2	**4b**	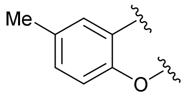	9.2
3	**4c**	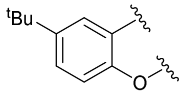	9.2
4	**4d**	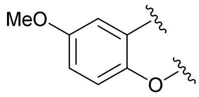	9.4
5	**4e**	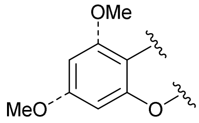	9.2 ; 9.7 [a]
6	**4f**	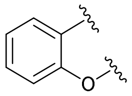	9.2
7	**4g**	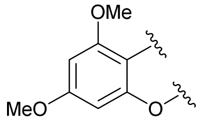	9.7
8	**4h**	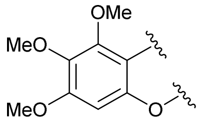	9.7
9	**4i**	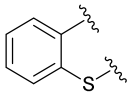	9.5
10	**4j**	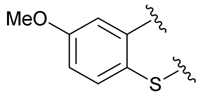	9.4
11	**4k**	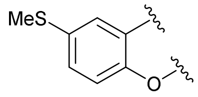	9.3
12	**4l**	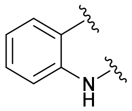	9.3

[a] 7-methoxy and 9-methoxy regioisomers respectively.

In-situ NMR Monitoring

A 5 mL stock solution of 37.5 mM of the acid **4** and 187.5 mM of maleimide **1a** was prepared in a 65 : 35 mixture of CD_3CN and D_2O. The solution was purged with argon for 5 minutes before transferring 1 mL to an NMR tube. The tube was irradiated using a 12 x 8 W BLB photoreactor (λ_{max} = 365 nm).

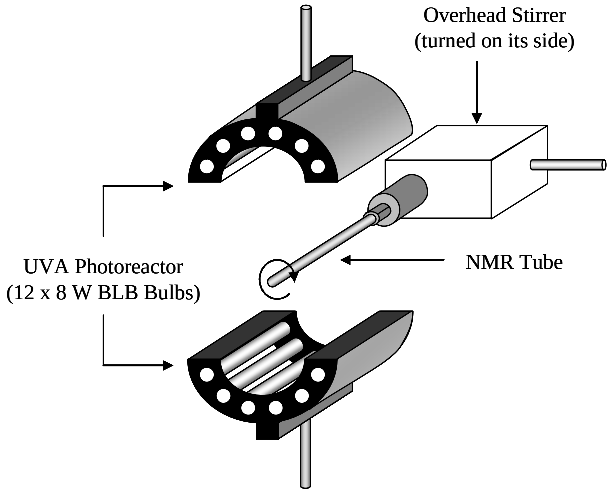

Figure S3. Irradiation setup for in-situ NMR monitoring.

Data for each reaction studied is provided below.

Reaction of phenoxyacetic acid 4a and maleimide 1a (Table 2, Entry 1).

Time (min)	[4a] (mM)	[1a] (mM)	[5a] (mM)	[7] (mM)
0	37.50	187.50	0.00	0.00
10	35.65	171.17	0.14	0.00
30	33.02	157.75	0.21	0.01
60	29.43	139.37	0.51	0.03
120	22.97	107.69	0.86	0.05
180	17.91	84.31	1.74	0.13
240	14.38	69.29	1.85	0.10
300	10.73	54.02	1.97	0.24
420	7.62	40.13	2.45	0.30
540	4.56	26.11	2.33	0.44

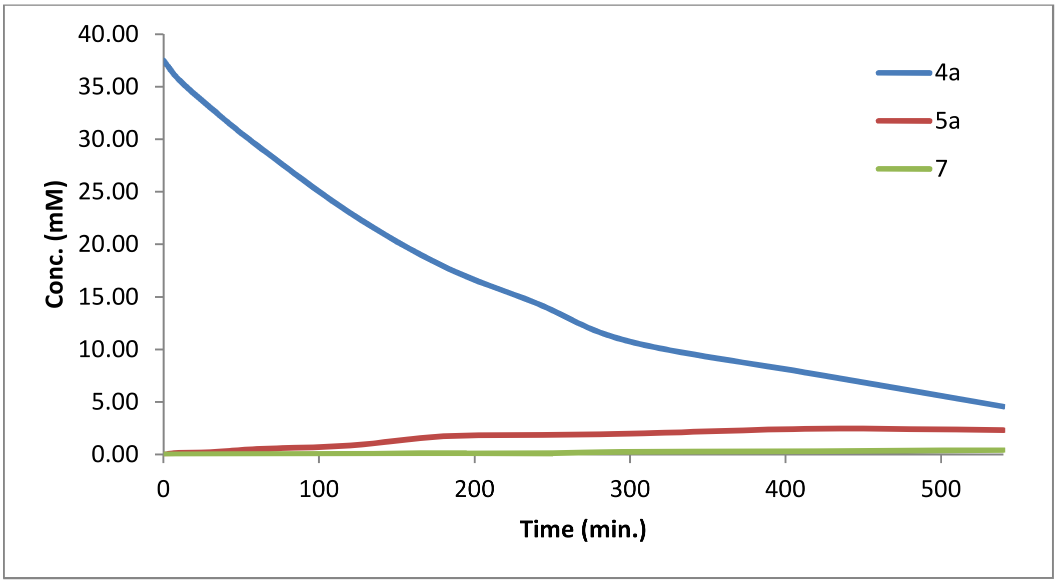

Figure S4. Reaction profile for **4a**

Reaction of 4-methylphenoxyacetic acid 4b and maleimide 1a (Table 2, Entry 2).

Time (min)	[4b] (mM)	[1a] (mM)	[5b] (mM)	[7] (mM)
0	37.50	187.50	0.00	0.00
10	32.45	126.83	1.12	0.61
20	26.93	115.05	3.31	1.44
40	18.27	83.96	5.19	2.12
60	12.70	67.49	7.28	2.80
90	8.71	63.70	11.56	4.31
120	4.76	43.47	12.05	4.61
150	1.94	41.87	15.53	6.38
180	0.71	31.62	14.61	5.41
240	0.91	27.27	12.18	6.49

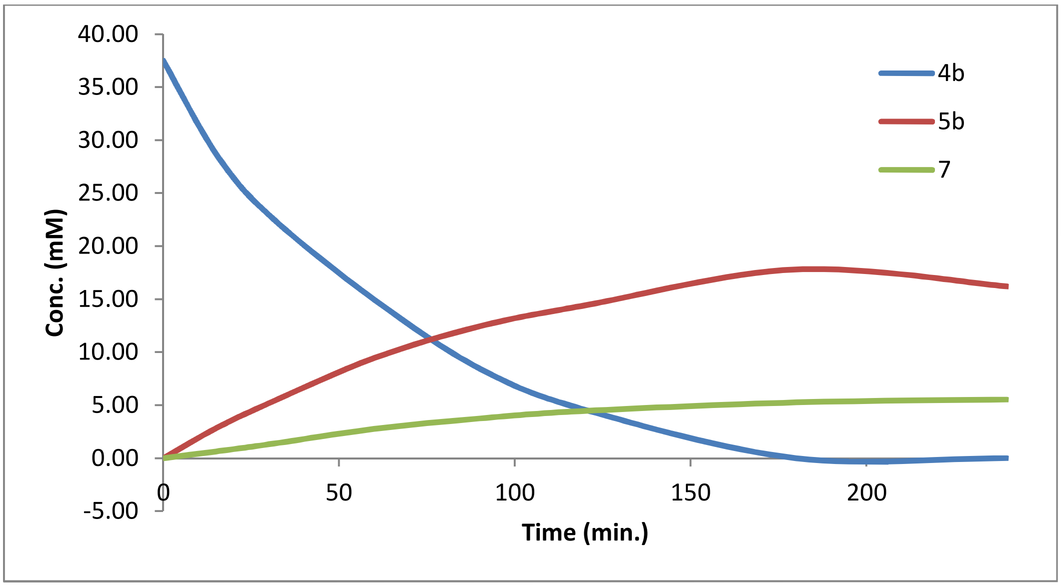

Figure S5. Reaction profile for **4b**

Reaction of 4-t-butylphenoxyacetic acid 4c and maleimide 1a (Table 2, Entry 3)

Time (min)	[4c] (mM)	[1a] (mM)	[5c] (mM)	[7] (mM)
0	37.50	187.50	0.00	0.00
10	33.79	144.36	1.50	0.40
30	26.82	98.87	4.52	1.28
60	19.15	54.63	8.01	2.40
120	9.32	15.53	12.09	4.00
180	5.80	5.61	14.14	4.50
240	4.06	2.68	14.81	4.69
300	2.76	2.17	14.68	4.82
420	1.82	1.34	14.74	4.74
540	0.12	2.98	17.27	5.00

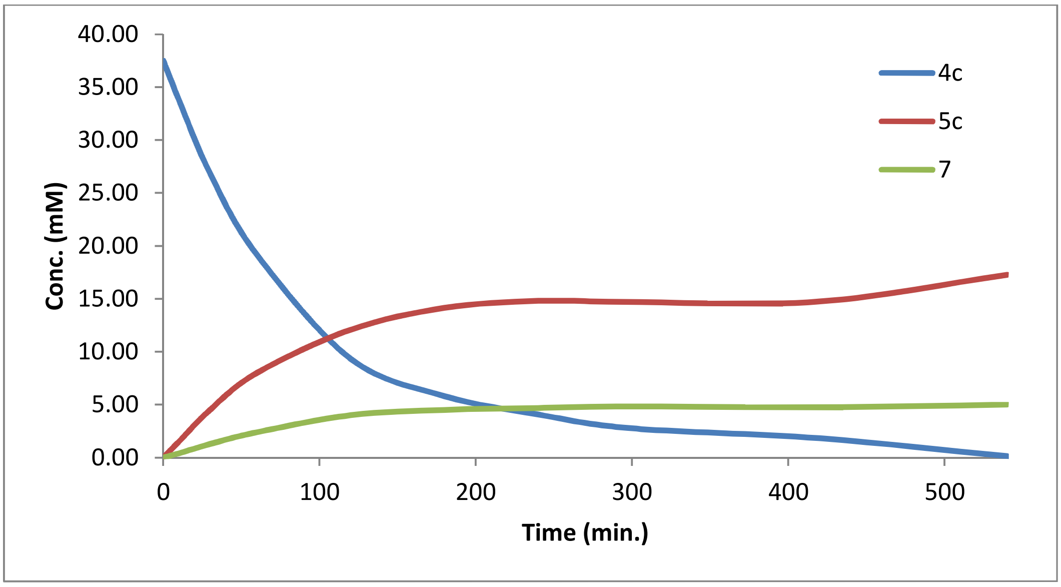

Figure S6. Reaction profile for **4c**

Reaction of 4-methoxyphenoxyacetic acid 4d and maleimide 1a (Table 2, Entry 4).

Time (min)	[4d] (mM)	[1a] (mM)	[5d] (mM)	[7] (mM)
0	37.50	187.50	0.00	0.00
10	33.42	122.23	1.94	0.47
20	26.17	93.80	3.87	0.96
40	20.15	74.21	6.64	1.72
60	15.72	58.60	9.31	2.41
90	11.33	42.21	12.18	3.03
120	8.17	32.04	14.35	3.80
150	5.96	26.34	15.60	3.95
180	4.85	23.36	18.84	5.26
240	0.00	14.13	22.06	5.14
300	0.03	11.75	20.89	5.31

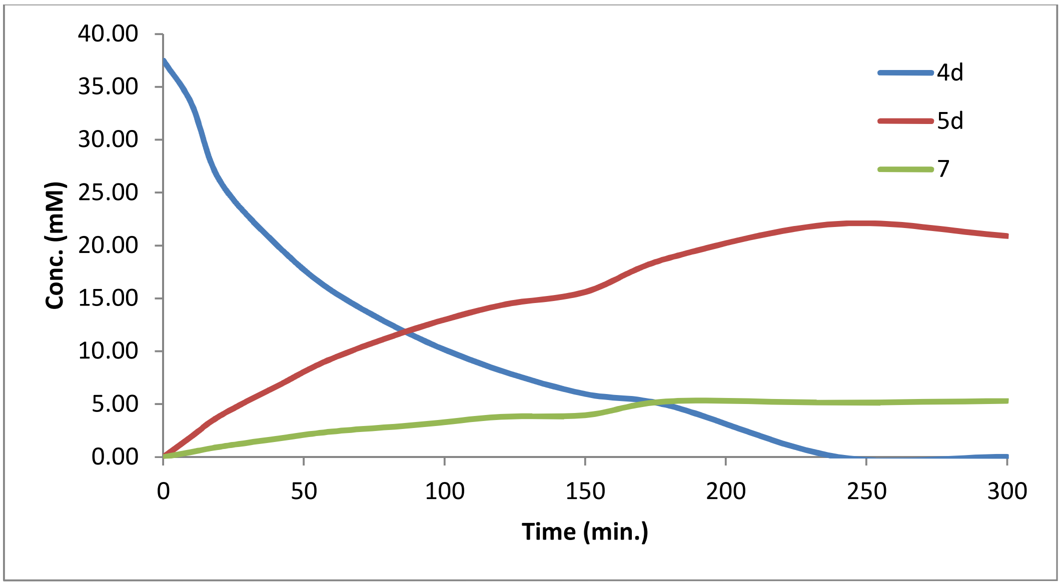

Figure S7. Reaction profile for **4d**

Reaction of 3-methoxyphenoxyacetic acid 4e and maleimide 1a (Table 2, Entry 5).

Time (min)	[4e] (mM)	[1a] (mM)	[5e] (mM)	[5e'] (mM)	[7] (mM)
0	37.50	187.50	0.00	0.00	0.00
15	32.13	119.52	3.40	1.37	0.60
30	27.03	106.46	6.38	2.11	1.13
60	18.80	96.69	12.45	3.68	2.31
90	12.13	75.00	17.57	4.05	3.40
120	6.39	48.31	22.08	4.78	4.32
180	-0.15	35.31	28.33	6.76	5.92
240	0.04	37.91	28.42	5.34	6.18

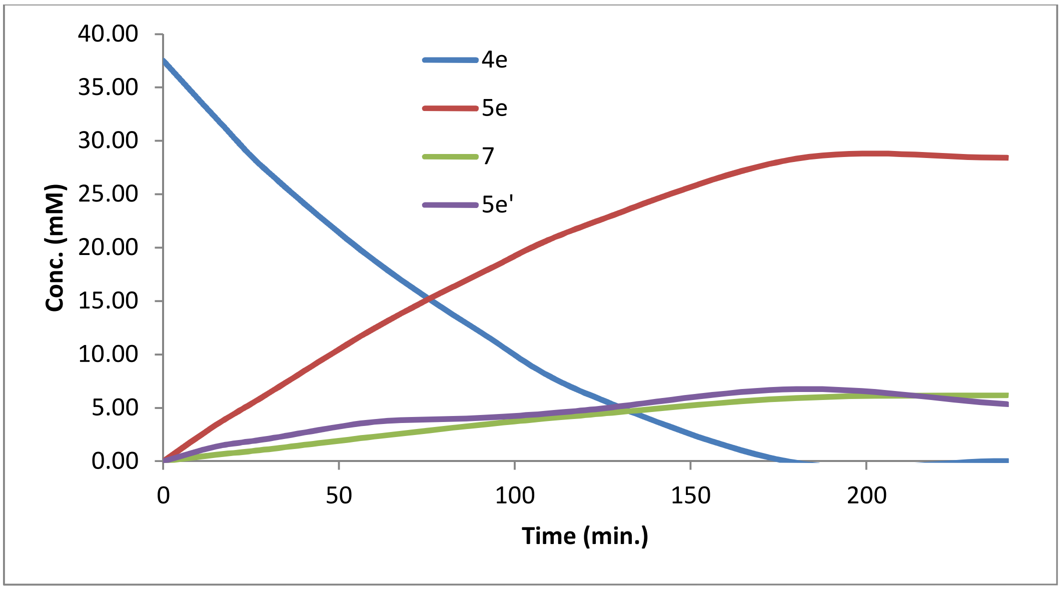

Figure S8. Reaction profile for **4e**

Reaction of 2-methoxyphenoxyacetic acid 4f and maleimide 1a (Table 2, Entry 6).

Time (min)	[4f] (mM)	[1a] (mM)	[5f] (mM)	[7] (mM)
0	37.50	187.50	0.00	0.00
15	25.86	118.09	6.05	0.37
30	20.90	84.36	10.60	0.66
60	12.12	45.82	16.26	0.99
120	5.17	19.51	20.15	1.14
180	2.12	8.58	18.93	1.43
240	0.67	4.58	20.17	1.64

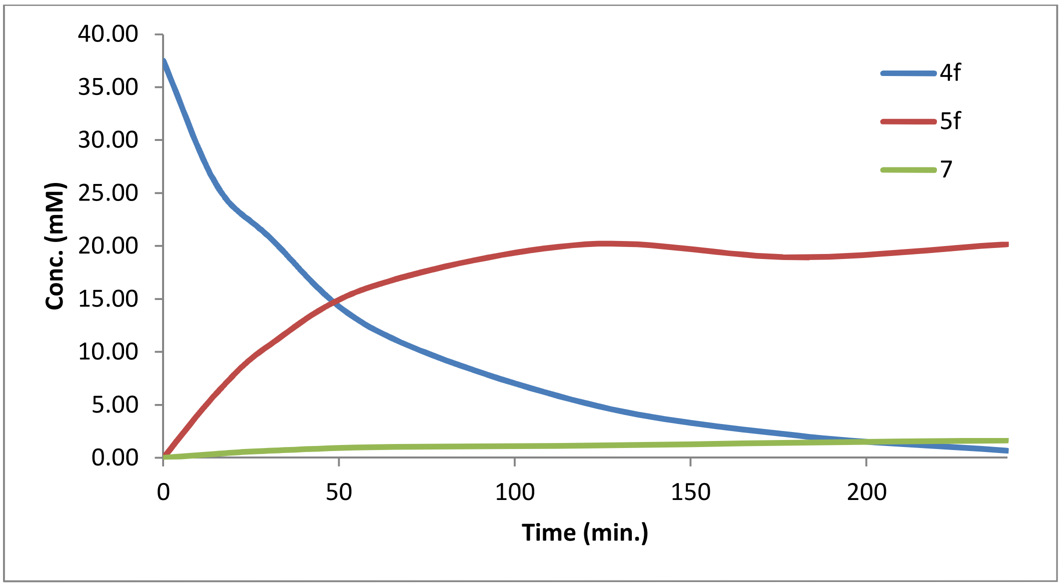

Figure S9. Reaction profile for **4f**

Reaction of 3,5-dimethoxyphenoxyacetic acid 4g and maleimide 1a (Table 2, Entry 7).

Time (min)	[4g] (mM)	[1a] (mM)	[5g] (mM)	[7] (mM)
0	37.50	187.50	0.00	0.00
10	34.59	166.71	3.15	0.37
30	28.57	143.33	8.26	1.07
60	21.60	114.31	15.56	2.08
120	9.68	75.93	26.83	3.69
180	2.25	54.30	34.99	4.98
240	0.28	42.99	36.92	5.11
0	37.50	187.50	0.00	0.00

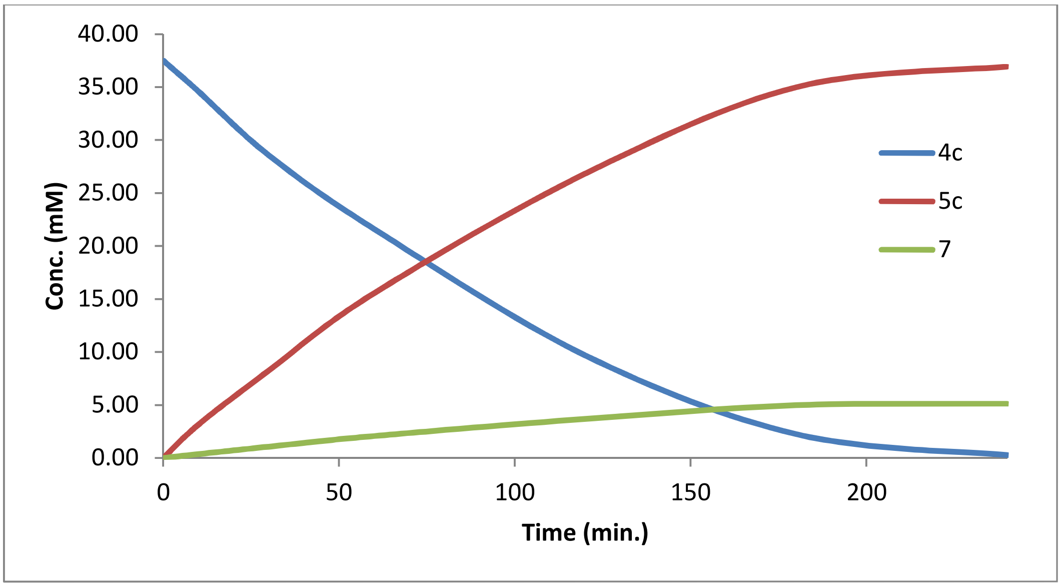

Figure S10. Reaction profile for **4g**

Reaction of 3,4,5-trimethoxyphenoxyacetic acid 4h and maleimide 1a (Table 2, Entry 8).

No reaction observed over 240 minutes.

Reaction of phenylthioacetic acid 4i and maleimide 1a (Table 2, Entry 9).

Time (min)	[4i] (mM)	[1a] (mM)	[5i] (mM)	[7] (mM)
0	37.50	187.50	0.00	0.00
10	32.45	126.83	1.12	0.61
20	26.93	115.05	3.31	1.44
40	18.27	83.96	5.19	2.12
60	12.70	67.49	7.28	2.80
90	8.71	63.70	11.56	4.31
120	4.76	43.47	12.05	4.61
150	1.94	41.87	15.53	6.38
180	0.71	31.62	14.61	5.41
240	0.91	27.27	12.18	6.49

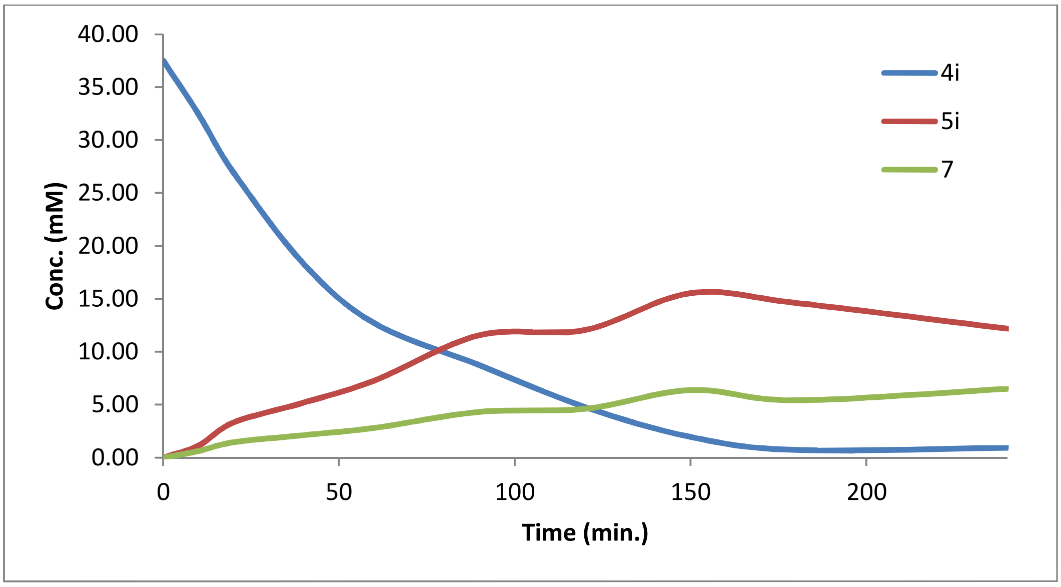

Figure S11. Reaction profile for **4i**

Reaction of 4-methoxyphenylthioacetic acid 4j and maleimide 1a (Table 2, Entry 11).

Time (min)	[4j] (mM)	[1a] (mM)	[5j] (mM)	[7] (mM)
0	37.50	187.50	0.00	0.00
2	35.50	125.46	1.29	0.20
20	33.37	120.12	2.48	0.75
40	29.49	102.46	4.28	1.31
60	26.72	91.34	5.90	1.77
120	19.78	64.97	9.64	2.56
180	14.07	45.96	10.30	3.04
240	11.21	38.60	13.67	3.89
300	8.86	33.09	14.76	4.01
420	8.27	29.34	14.93	4.45
540	7.30	26.86	14.46	4.37

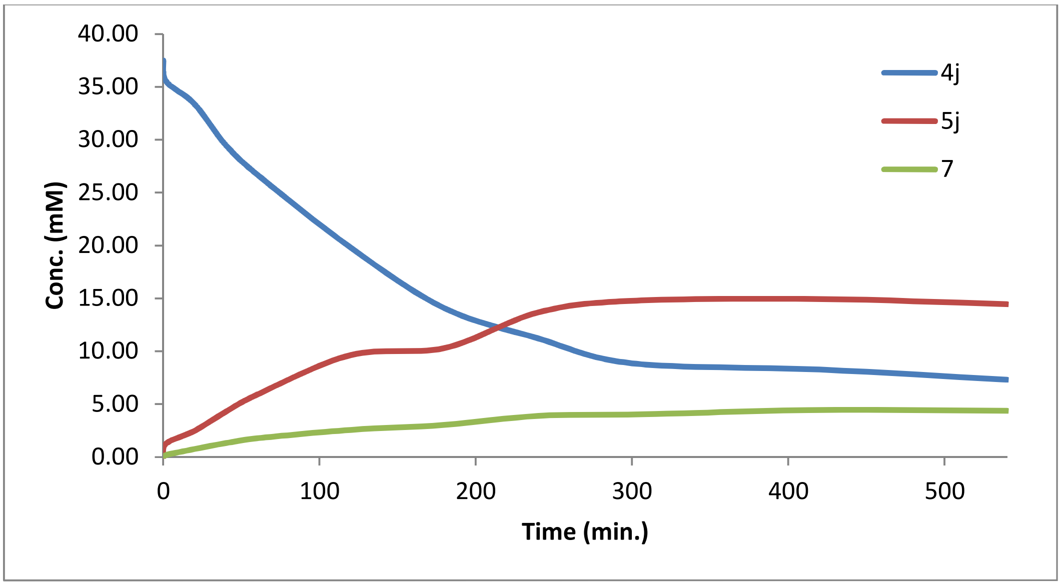

Figure S12. Reaction profile for **4j**

Reaction of 4-methylsulfanylphenoxyacetic acid 4k and maleimide 1a (Table 2, Entry 12).

Time (min)	[4k] (mM)	[1a] (mM)	[5k] (mM)	[7] (mM)
0	37.50	187.50	0.38	0.00
20	34.67	172.21	1.02	0.23
40	33.09	158.72	1.89	0.36
60	30.56	144.93	1.77	0.29
120	28.71	126.51	2.98	0.45
180	27.71	112.92	4.04	0.77
240	24.93	98.48	3.76	0.57
300	24.55	89.15	4.81	0.79
420	22.71	70.93	5.57	0.89
540	22.04	61.85	6.71	1.19

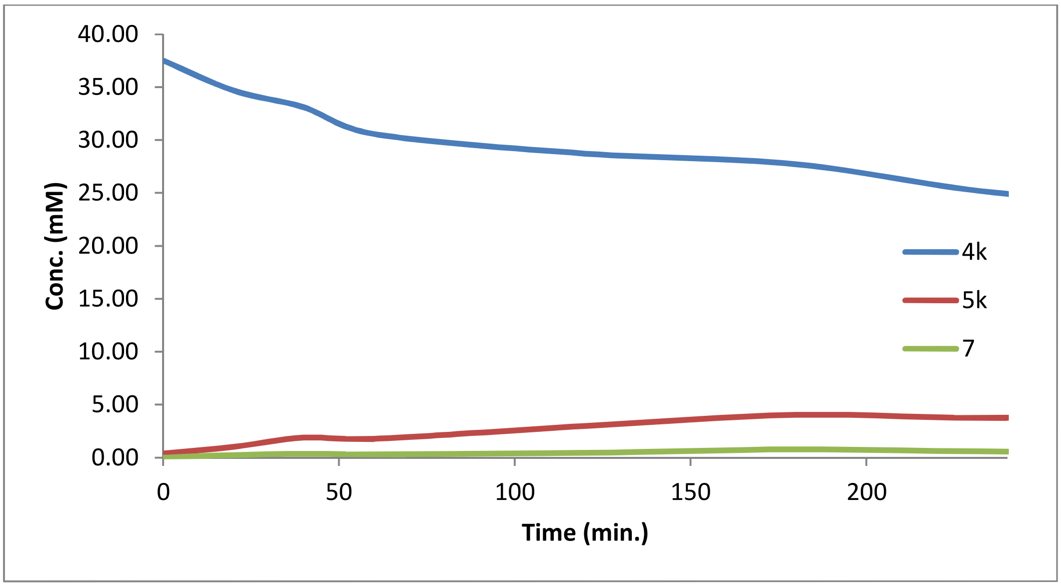

Figure S13. Reaction profile for 4k

Reaction of *N*-phenyl glycine acid 4l and maleimide 1a (Table 2, Entry 13).

Time (min)	[4k] (mM)	[1a] (mM)	[5k] (mM)	[7] (mM)
0	37.50	187.50	0.06	0.01
5	36.05	149.88	2.40	0.35
10	33.63	151.14	4.84	0.72
20	26.22	132.95	9.56	1.53
40	21.22	109.57	17.70	2.73
60	12.55	90.02	25.85	4.14
90	1.23	68.00	35.97	5.93
120	2.89	69.55	34.46	6.42

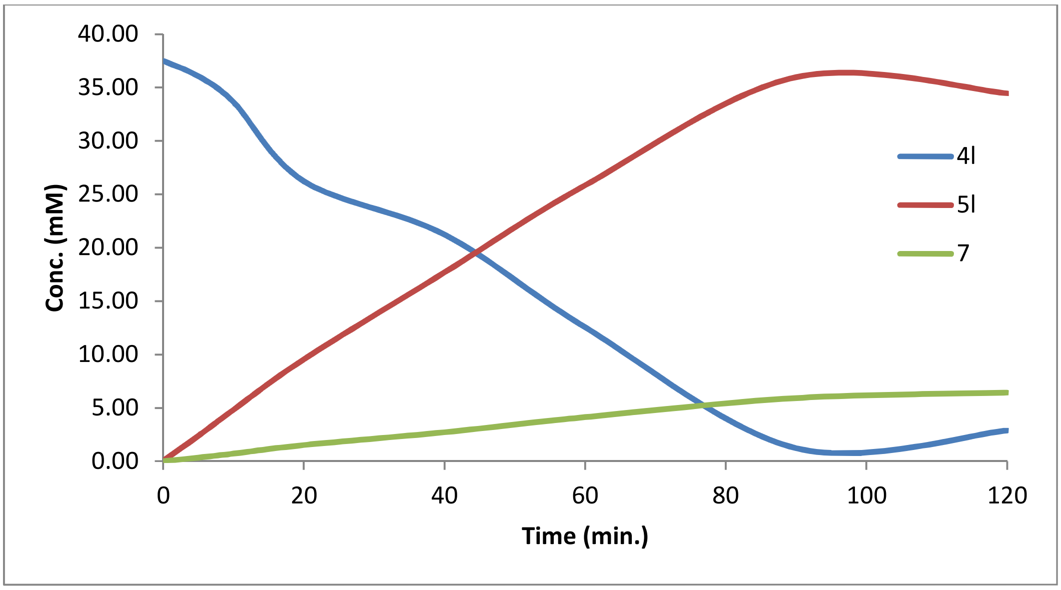

Figure S14. Reaction profile for **4l**

Figure S15. Profile showing Lamp Output and Pyrex Absorbance

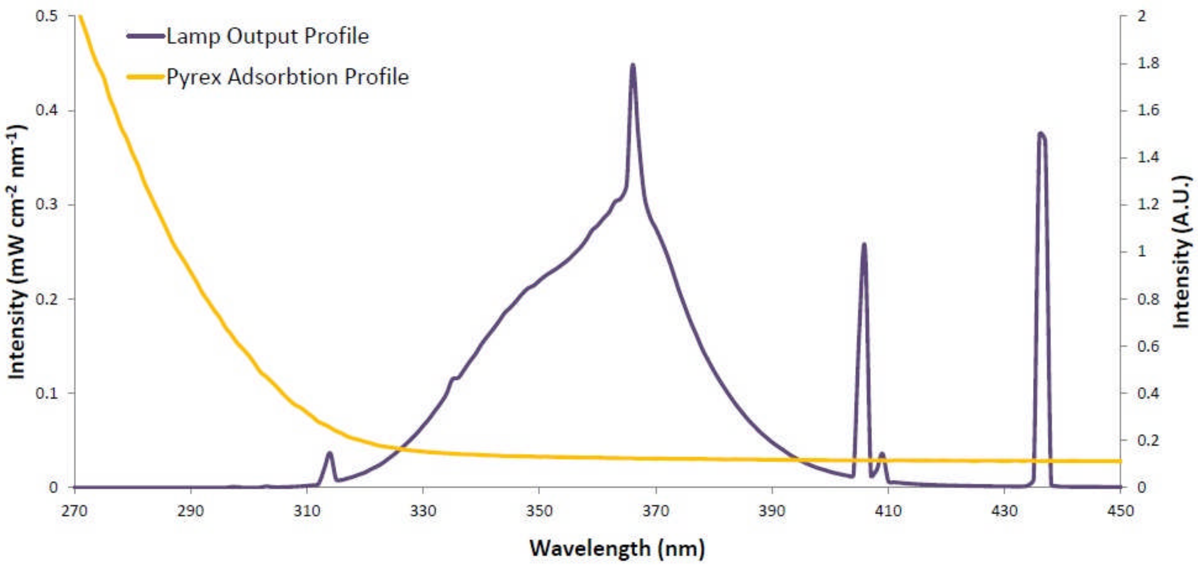

Figure S16. Profile showing scale expansion of absorbance of maleimide **1a**

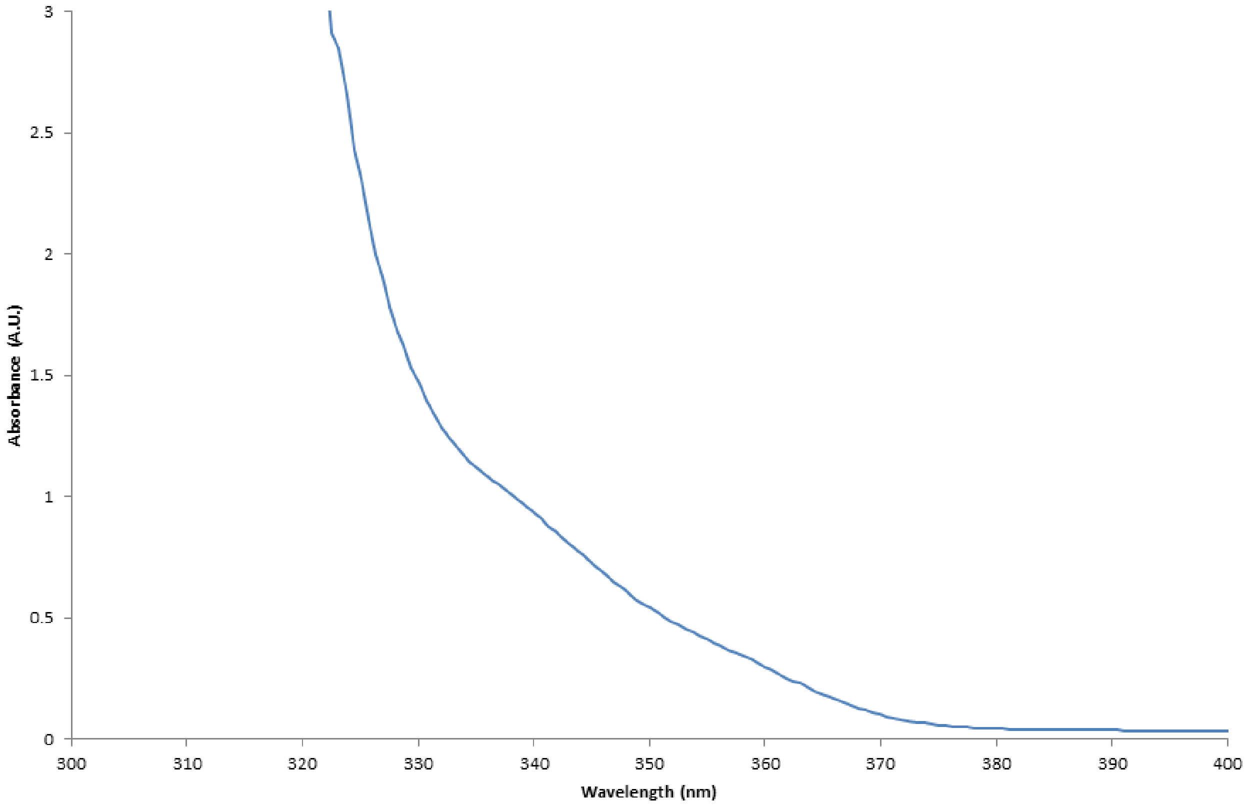

Figure S17. Profile showing absorbances of acids **4b-l**

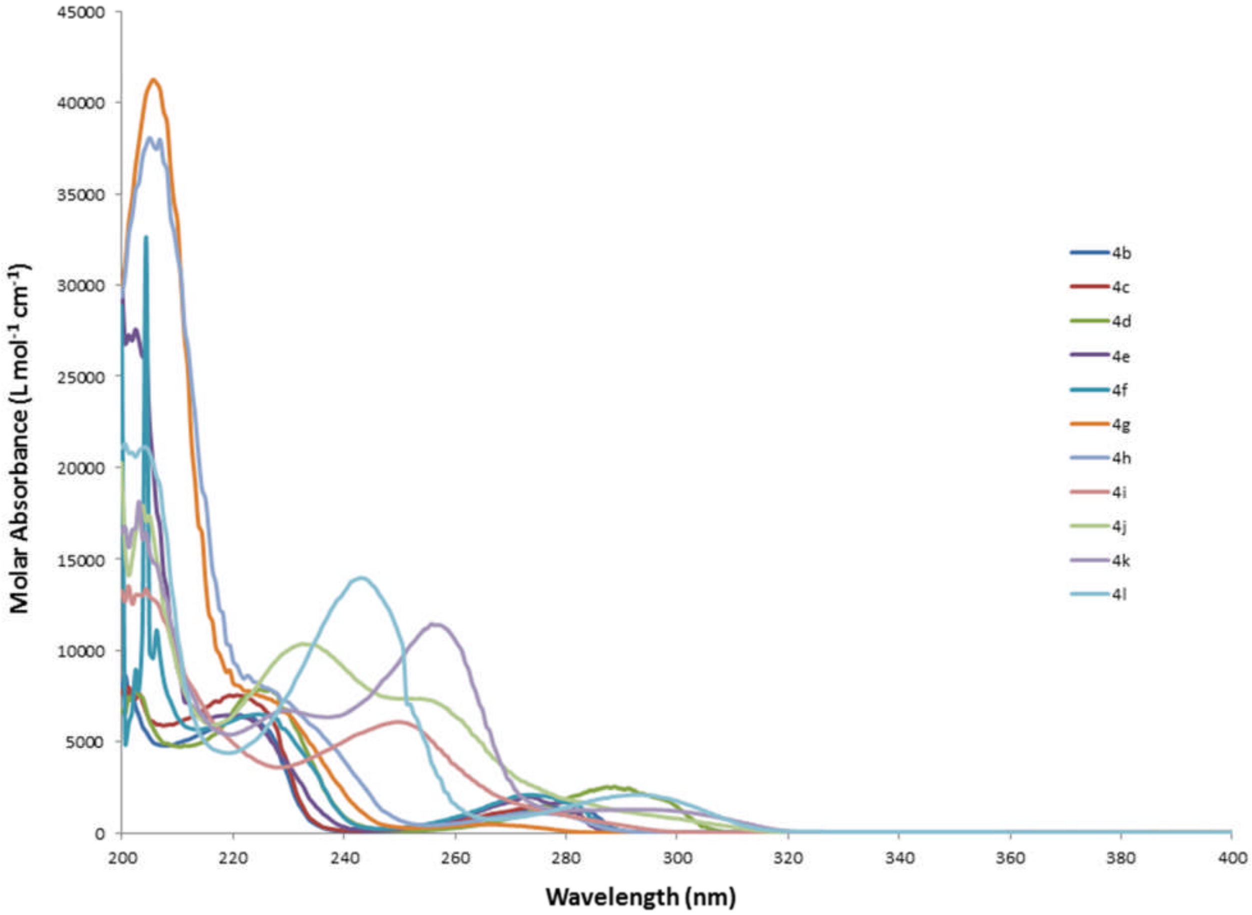

EPR Spectroscopy

EPR spectra were obtained at 9.5 GHz with 100 kHz modulation employing a Bruker EMX 10/12 spectrometer fitted with a rectangular ER4122 SP resonant cavity. Stock solutions of each acid (10 to 50 mg) in benzene or *tert*-butylbenzene (0.5 mL) were prepared and sonicated if necessary. An aliquot (0.2 mL), to which any additional reactant had been added, was placed in a 5 mm o.d. quartz tube, de-aerated by bubbling nitrogen for 15 min. Photolysis in the resonant cavity was by unfiltered light from a 500 W super pressure mercury arc lamp. In all cases where spectra were obtained, hfs were assigned with the aid of computer simulations using the Bruker SimFonia and NIEHS Winsim2002 software packages. For kinetic measurements, precursor samples were used mainly in 'single shot' experiments, i.e. new samples were prepared for each temperature and each concentration to minimize sample depletion effects. EPR signals were digitally filtered and double integrated using the Bruker WinEPR software and radical concentrations were calculated by reference to the double integral of the signal from a known concentration of the stable radical DPPH [1×10^{-3} M in PhMe], run under identical conditions. The majority of EPR spectra were recorded with 2.0 mW power, 0.8 G_{pp} modulation intensity and gain of *ca.* 10^6.

Sample EPR spectra of radical cations

Fig. S18. EPR spectrum on UV photolysis of 1,4-dimethoxybenzene in PhH at 300 K.

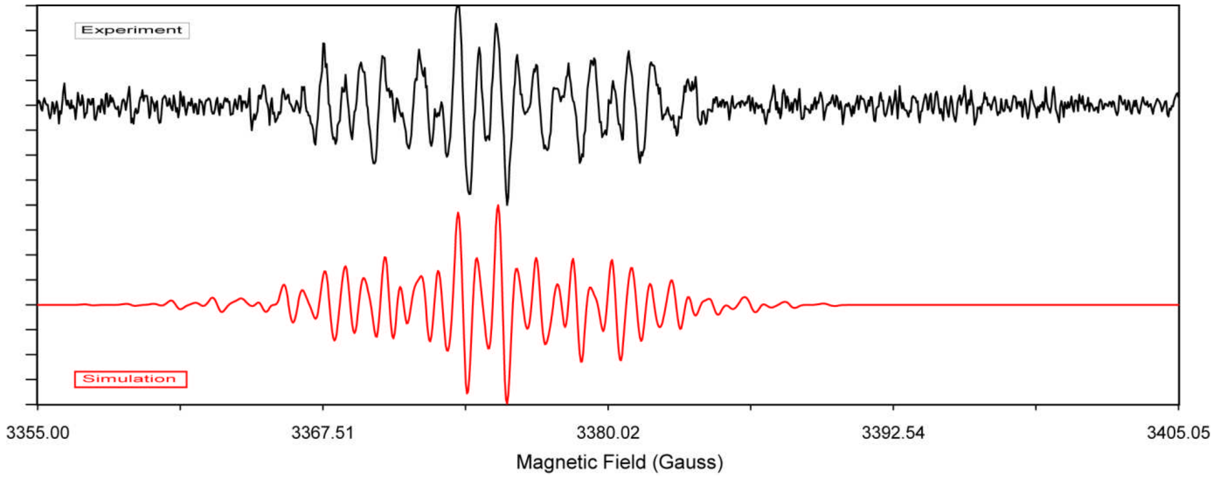

Simulation with two radical cations (*transoid* and *cisoid*): one 30% having hfs/G: a(2H) = 1.8, a(2H) = 3.0, a(2H) = 3.8 and a second 70% with: a(2H) = 1.7, a(2H) 2.7, a(2H) = 3.3 G.

Figure S19. EPR spectrum obtained on UV photolysis of 4-*t*-Bu-phenoxyacetic acid 4c in PhH at 300 K.

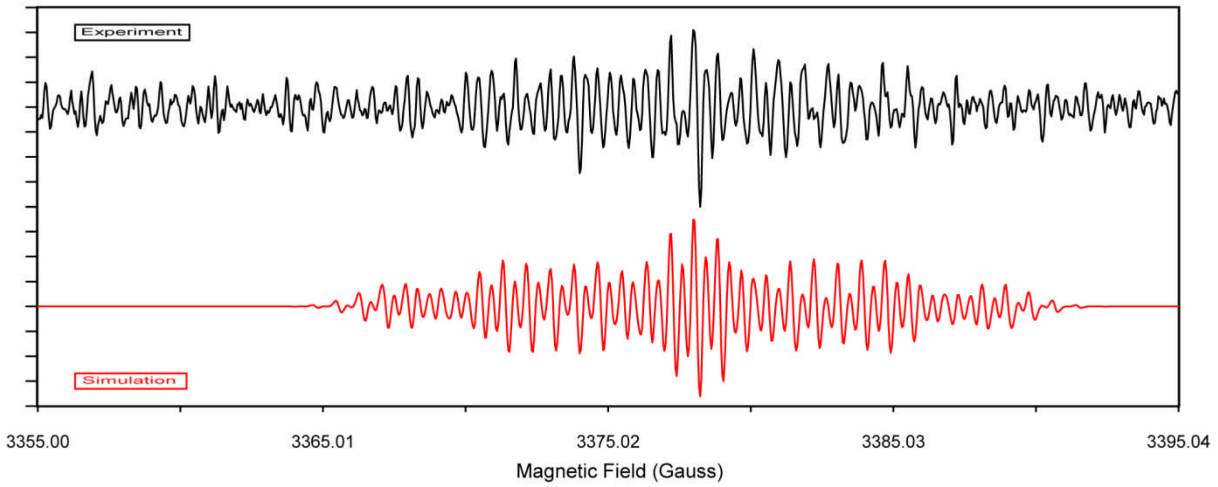

EPR parameters in main text Table 4.

Figure S20. EPR spectrum obtained on UV photolysis of benzyl (4-methoxyphenoxy)acetate 15 in PhH at 300K.

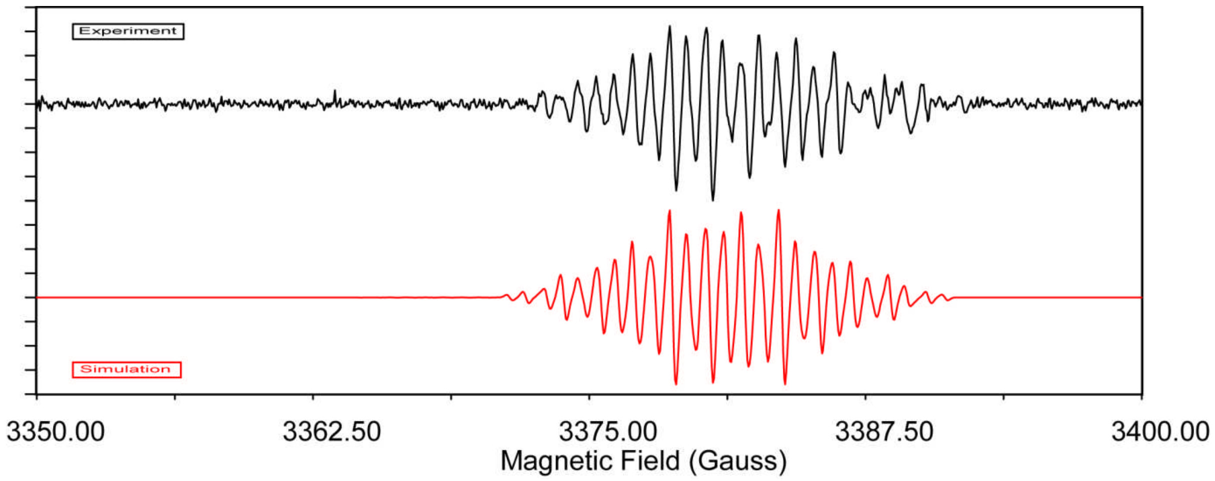

EPR parameters in table 4 of the main text.

Figure S21. EPR spectrum obtained on UV photolysis of methyl (4-methylthiophenoxy) acetate 15 in PhH at 300K.

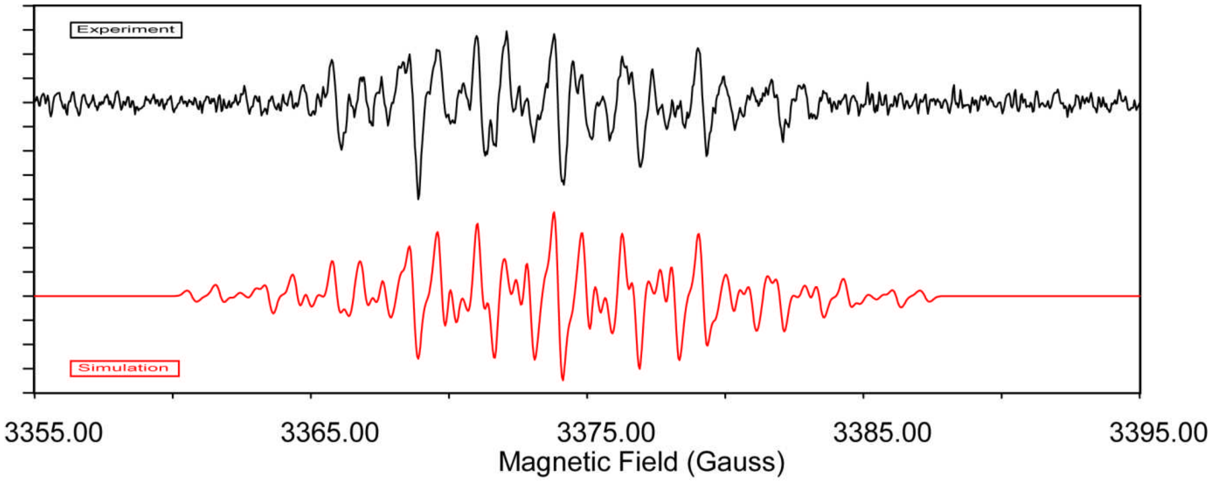

EPR parameters in Table 4 of the main text.

Figure S22. EPR spectrum obtained on UV photolysis of 3,4,5-trimethoxyphenoxyacetic acid 4h in PhH at 300K.

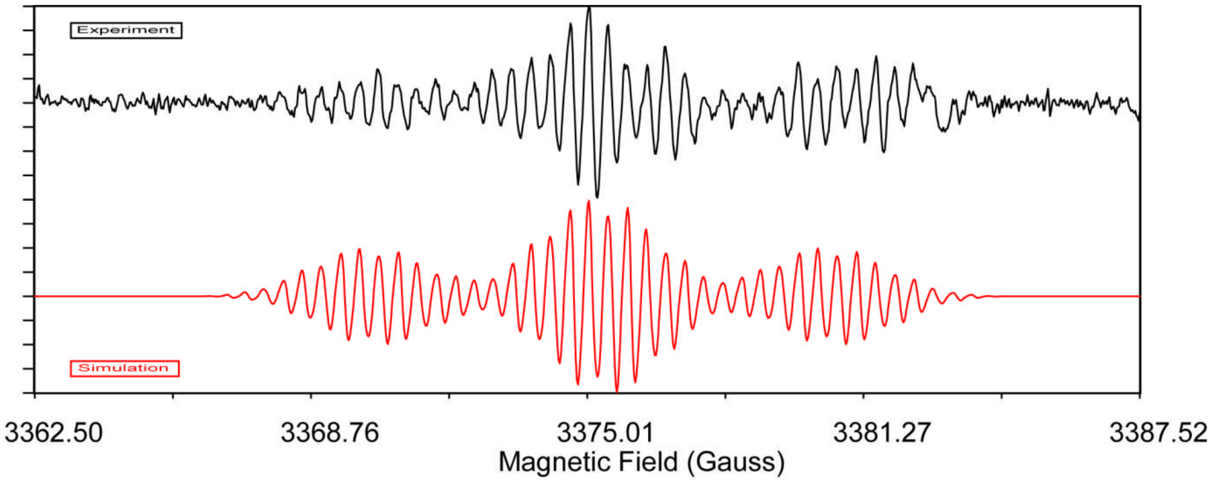

EPR parameters in Table 4 of the main text.

Figure S23. EPR spectrum obtained on UV photolysis of methyl (3,4,5-trimethoxyphenoxy)acetate 17 in PhH at 300K.

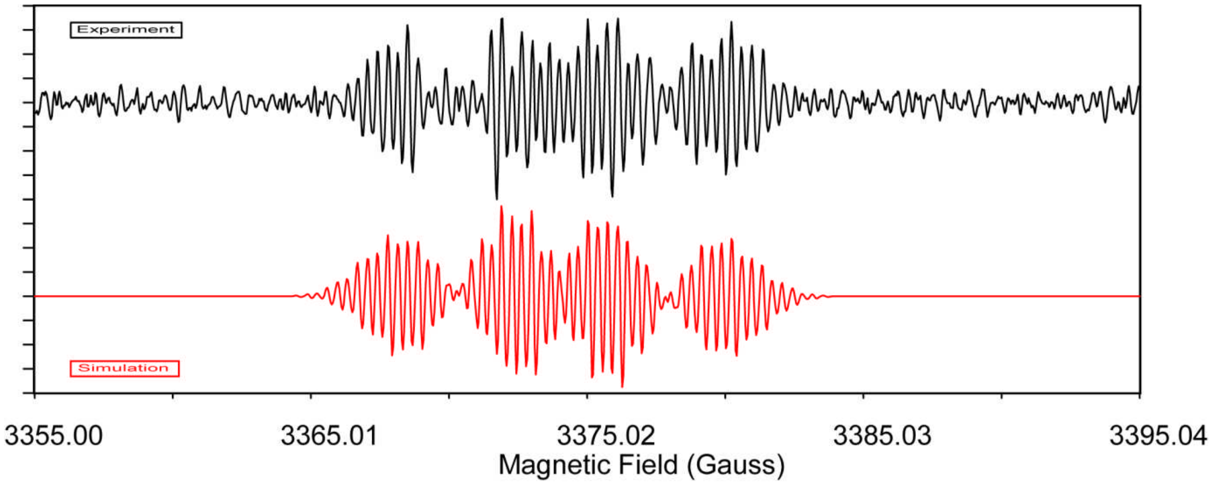

EPR parameters in Table 4 of the main text.

Figure S24. EPR spectrum obtained on UV photolysis of dibenzyl 2,2'-(1,4-phenylenebis(oxy))diacetate 18 in PhH at 300K.

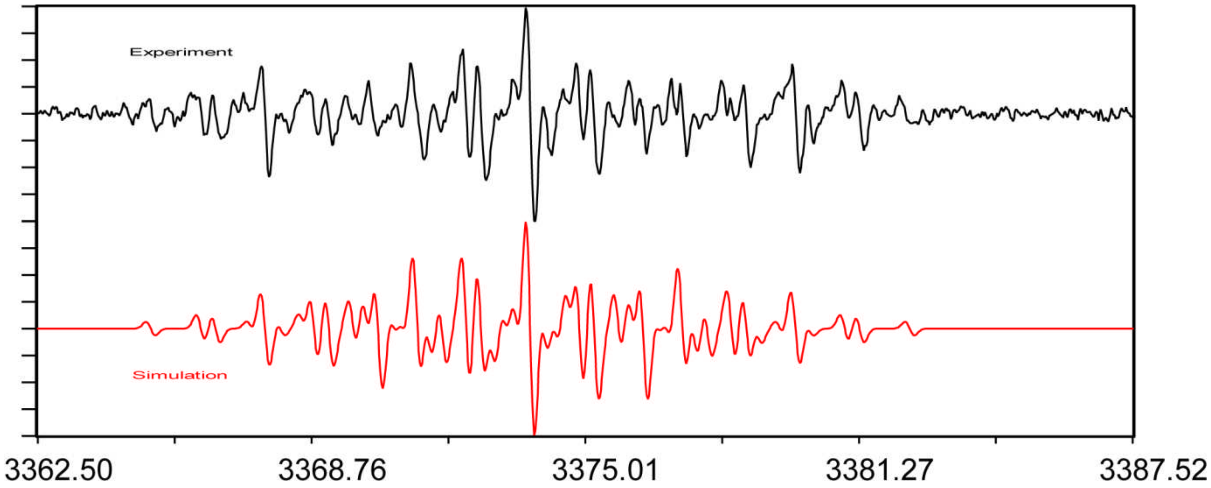

EPR parameters in Table 4 of main text.

Computational Methods. The ground-state geometries and energies of the precursor acids and their ions were investigated using the Gaussian 09 program package. The standard UB3LYP functional with the split-valence and with the aug-cc-pvtz basis sets was employed. Geometries were fully optimized with both basis sets without any symmetry constraints for all model compounds. Optimized structures were characterized as minima or saddle points by frequency calculations at the 6-311+G(2d,p) level. The experimental EPR data was all obtained in the non-polar hydrocarbon solvents *tert*-butylbenzene or benzene. Solvent effects, particularly differences in solvation between the neutral reactants and neutral transition states, are therefore expected to be minimal for the EPR work. However, the addition/cyclisation reactions were carried out in acetonitrile/water. In an attempt to model the effect of solvent the CPCM polarizable conductor calculation model was then applied, with acetonitrile as the solvent, and with the aug-cc-pvtz basis set and geometry.

Gaussian 09, Revision A.02, M. J. Frisch, G. W. Trucks, H. B. Schlegel, G. E. Scuseria, M. A. Robb, J. R. Cheeseman, G. Scalmani, V. Barone, B. Mennucci, G. A. Petersson, H. Nakatsuji, M. Caricato, X. Li, H. P. Hratchian, A. F. Izmaylov, J. Bloino, G. Zheng, J. L. Sonnenberg, M. Hada, M. Ehara, K. Toyota, R. Fukuda, J. Hasegawa, M. Ishida, T. Nakajima, Y. Honda, O. Kitao, H. Nakai, T. Vreven, J. A. Montgomery, Jr., J. E. Peralta, F. Ogliaro, M. Bearpark, J. J. Heyd, E. Brothers, K. N. Kudin, V. N. Staroverov, R. Kobayashi, J. Normand, K. Raghavachari, A. Rendell, J. C. Burant, S. S. Iyengar, J. Tomasi, M. Cossi, N. Rega, J. M. Millam, M. Klene, J. E. Knox, J. B. Cross, V. Bakken, C. Adamo, J. Jaramillo, R. Gomperts, R. E. Stratmann, O. Yazyev, A. J. Austin, R. Cammi, C. Pomelli, J. W. Ochterski, R. L. Martin, K. Morokuma, V. G. Zakrzewski, G. A. Voth, P. Salvador, J. J. Dannenberg, S. Dapprich, A. D. Daniels, O. Farkas, J. B. Foresman, J. V. Ortiz, J. Cioslowski, and D. J. Fox, Gaussian, Inc., Wallingford CT, 2009.

DFT Optimised Structures

Optimized coordinates for acid $PhOCH_2CO_2H$ (4a).

UB3LYP/6-311+G(2d,p)

Center Number	Atomic Number	Atomic Type	Coordinates (Angstroms) X	Y	Z
1	6	0	2.710630	-1.290383	0.000038
2	6	0	1.326149	-1.118237	0.000072
3	6	0	0.798395	0.171704	0.000003
4	6	0	1.652347	1.277063	-0.000098
5	6	0	3.024514	1.088829	-0.000129
6	6	0	3.564448	-0.197444	-0.000062
7	1	0	3.114770	-2.295832	0.000092
8	1	0	0.685048	-1.988686	0.000154
9	1	0	1.217987	2.268842	-0.000151
10	1	0	3.679338	1.952217	-0.000208
11	8	0	-0.540574	0.464123	0.000033
12	6	0	-1.454419	-0.606602	0.000059
13	1	0	-1.334256	-1.241817	0.885026
14	1	0	-1.334221	-1.241893	-0.884846
15	6	0	-2.889568	-0.121600	0.000013
16	8	0	-3.815049	-0.892409	-0.000019
17	8	0	-3.029095	1.212885	0.000062
18	1	0	-3.983572	1.391162	0.000047
19	1	0	4.637676	-0.340769	-0.000087

Optimized coordinates for radical cation $4a^{+\bullet}$

UB3LYP/6-311+G(2d,p)

Center Number	Atomic Number	Atomic Type	Coordinates (Angstroms) X	Y	Z
1	6	0	2.720917	-1.297233	0.000077
2	6	0	1.358319	-1.183714	0.000060
3	6	0	0.785292	0.123386	-0.000041
4	6	0	1.619162	1.292988	-0.000124
5	6	0	2.978897	1.149871	-0.000106
6	6	0	3.542443	-0.140691	-0.000006
7	1	0	3.182971	-2.276248	0.000155
8	1	0	0.730494	-2.063633	0.000125
9	1	0	1.132668	2.260185	-0.000200
10	1	0	3.621459	2.020630	-0.000167
11	8	0	-0.495997	0.374761	-0.000064
12	6	0	-1.499997	-0.673606	-0.000007
13	1	0	-1.382049	-1.287559	0.893806
14	1	0	-1.381968	-1.287755	-0.893671
15	6	0	-2.905758	-0.091199	-0.000159
16	8	0	-3.846009	-0.830527	0.000198
17	8	0	-2.938964	1.242016	0.000056
18	1	0	-3.870883	1.522530	0.000259
19	1	0	4.619418	-0.256966	0.000009

Optimized coordinates for acid 4-$MePhOCH_2CO_2H$ (4b).

UB3LYP/6-311+G(2d,p)

Center Number	Atomic Number	Atomic Type	Coordinates (Angstroms) X	Y	Z
1	6	0	2.261300	-1.190306	0.002081
2	6	0	0.871425	-1.053857	0.001529
3	6	0	0.309321	0.218306	-0.000878
4	6	0	1.141846	1.339835	-0.002762
5	6	0	2.515869	1.181345	-0.002189
6	6	0	3.107947	-0.088507	0.000247

7	1	0	2.684135	-2.189068	0.003964
8	1	0	0.256252	-1.943027	0.002970
9	1	0	0.689549	2.323704	-0.004639
10	1	0	3.146232	2.064434	-0.003668
11	8	0	-1.037513	0.480749	-0.001593
12	6	0	-1.921995	-0.613200	0.000278
13	1	0	-1.784637	-1.244066	0.886178
14	1	0	-1.784904	-1.247233	-0.883270
15	6	0	-3.369605	-0.167287	-0.000202
16	8	0	-4.274716	-0.962151	-0.005157
17	8	0	-3.545444	1.163121	0.006305
18	1	0	-4.504489	1.314790	0.005805
19	6	0	4.608213	-0.241986	0.000799
20	1	0	5.056975	0.228626	0.880126
21	1	0	4.899023	-1.293578	0.002655
22	1	0	5.057305	0.225602	-0.879969

Optimized coordinates for radical cation of 4-MePhOCH$_2$CO$_2$H (4b$^{+\bullet}$).

UB3LYP/6-311+G(2d,p)

Center Number	Atomic Number	Atomic Type	Coordinates (Angstroms) X	Y	Z
1	6	0	2.264147	-1.212062	0.000033
2	6	0	0.902343	-1.122116	0.000054
3	6	0	0.302409	0.169601	-0.000002
4	6	0	1.118336	1.345770	-0.000080
5	6	0	2.477720	1.227308	-0.000100
6	6	0	3.095442	-0.049088	-0.000044
7	1	0	2.735059	-2.187997	0.000075
8	1	0	0.294504	-2.015957	0.000113
9	1	0	0.620775	2.307289	-0.000122
10	1	0	3.097776	2.114903	-0.000161
11	8	0	-0.986899	0.397132	0.000012
12	6	0	-1.959405	-0.675720	0.000081
13	1	0	-1.827961	-1.288285	0.893142
14	1	0	-1.827949	-1.288417	-0.892887
15	6	0	-3.380708	-0.133729	0.000031
16	8	0	-4.302281	-0.897026	0.000069
17	8	0	-3.452742	1.198555	-0.000033
18	1	0	-4.392747	1.449982	-0.000051
19	6	0	4.572278	-0.197940	-0.000044
20	1	0	4.895982	-0.776016	0.873921
21	1	0	4.895960	-0.776503	-0.873688
22	1	0	5.088604	0.759571	-0.000301

Optimized coordinates for acid 4-t-BuPhOCH$_2$CO$_2$H (4c).

UB3LYP/6-311+G(2d,p)

Center Number	Atomic Number	Atomic Type	Coordinates (Angstroms) X	Y	Z
1	6	0	-1.154300	1.112051	0.000024
2	6	0	0.238813	1.000818	0.000058
3	6	0	0.828388	-0.256973	0.000064
4	6	0	0.015165	-1.391673	0.000055
5	6	0	-1.360667	-1.257535	0.000024
6	6	0	-1.988701	-0.001343	-0.000007
7	1	0	-1.573716	2.108824	0.000033
8	1	0	0.834154	1.903562	0.000074
9	1	0	0.482788	-2.368461	0.000072
10	1	0	-1.958930	-2.160825	0.000019
11	8	0	2.179745	-0.492524	0.000109
12	6	0	3.040582	0.620408	-0.000001
13	1	0	2.889322	1.249708	0.884617
14	1	0	2.889239	1.249581	-0.884698
15	6	0	4.497532	0.206451	-0.000028
16	8	0	5.384952	1.021074	-0.000022
17	8	0	4.702818	-1.119782	-0.000094

18	1	0	5.665017	-1.249950	-0.000118
19	6	0	-3.522139	0.095290	-0.000028
20	6	0	-4.084336	-0.600244	-1.259482
21	1	0	-3.808488	-1.655602	-1.297596
22	1	0	-5.176259	-0.541416	-1.271040
23	1	0	-3.708915	-0.124078	-2.168269
24	6	0	-4.013405	1.552225	-0.000139
25	1	0	-5.105622	1.570655	-0.000170
26	1	0	-3.675281	2.095983	0.885080
27	1	0	-3.675218	2.095847	-0.885419
28	6	0	-4.084351	-0.600066	1.259526
29	1	0	-5.176272	-0.541188	1.271086
30	1	0	-3.808537	-1.655427	1.297735
31	1	0	-3.708895	-0.123803	2.168247

Optimized coordinates for radical cation of 4-t-BuPhOCH$_2$CO$_2$H (4c$^{+\bullet}$).

UB3LYP/6-311+G(2d,p)

Center Number	Atomic Number	Atomic Type	Coordinates (Angstroms) X	Y	Z
1	6	0	-1.171460	-1.104737	-0.000585
2	6	0	0.192073	-1.048539	-0.000899
3	6	0	0.823882	0.224364	-0.000811
4	6	0	0.032807	1.414754	-0.000487
5	6	0	-1.330005	1.329562	-0.000118
6	6	0	-1.990391	0.072496	-0.000074
7	1	0	-1.649922	-2.074298	-0.000739
8	1	0	0.775818	-1.958315	-0.001293
9	1	0	0.549922	2.366017	-0.000522
10	1	0	-1.911841	2.239482	0.000156
11	8	0	2.120014	0.422426	-0.001117
12	6	0	3.060278	-0.676245	-0.001020
13	1	0	2.913237	-1.285445	-0.894087
14	1	0	2.911740	-1.286756	0.890882
15	6	0	4.496428	-0.176069	0.000546
16	8	0	5.397145	-0.964393	0.001698
17	8	0	4.607895	1.153889	0.000352
18	1	0	5.555091	1.376224	0.001300
19	6	0	-3.494456	-0.065591	0.000366
20	6	0	-4.221017	1.288101	0.000782
21	1	0	-3.989368	1.880538	-0.887002
22	1	0	-5.297798	1.116905	0.000980
23	1	0	-3.988969	1.880233	0.888663
24	6	0	-3.917150	-0.863154	1.267594
25	1	0	-5.003040	-0.970951	1.262076
26	1	0	-3.486444	-1.864670	1.297948
27	1	0	-3.632284	-0.336130	2.179763
28	6	0	-3.917960	-0.862863	-1.266714
29	1	0	-5.003818	-0.970927	-1.260425
30	1	0	-3.633887	-0.335494	-2.178931
31	1	0	-3.487035	-1.864266	-1.297711

Optimized coordinates 4-MeOPhOCH$_2$CO$_2$H cis 4dc

UB3LYP/6-311+G(2d,p)

Center Number	Atomic Number	Atomic Type	Coordinates (Angstroms) X	Y	Z
1	6	0	1.925133	-0.942322	0.000160
2	6	0	0.526093	-0.887666	-0.000051
3	6	0	-0.123594	0.338454	-0.000233
4	6	0	0.630585	1.516422	-0.000364
5	6	0	2.008848	1.461919	-0.000141
6	6	0	2.671684	0.228451	0.000199
7	1	0	2.404632	-1.910991	0.000162
8	1	0	-0.025581	-1.817543	-0.000101
9	1	0	0.111765	2.466762	-0.000648
10	1	0	2.599381	2.369544	-0.000294

11	8	0	-1.487997	0.508513	-0.000363
12	6	0	-2.290210	-0.645530	-0.000097
13	1	0	-2.107280	-1.266858	0.884700
14	1	0	-2.107580	-1.267095	-0.884780
15	6	0	-3.766526	-0.307267	0.000091
16	8	0	-4.611123	-1.166337	-0.000265
17	8	0	-4.039829	1.006648	0.000676
18	1	0	-5.007458	1.086938	0.000751
19	8	0	4.038210	0.287084	0.000254
20	6	0	4.759076	-0.934336	0.000052
21	1	0	5.812115	-0.660793	-0.000065
22	1	0	4.539798	-1.528014	0.893392
23	1	0	4.539586	-1.527960	-0.893221

Optimized coordinates 4-MeOPhOCH$_2$CO$_2$H trans 4dt

UB3LYP/6-311+G(2d,p)

Center Number	Atomic Number	Atomic Type	Coordinates (Angstroms) X	Y	Z
1	6	0	1.767951	-1.352438	-0.000422
2	6	0	0.393603	-1.147135	-0.000379
3	6	0	-0.115163	0.151970	-0.000347
4	6	0	0.764844	1.229098	-0.000131
5	6	0	2.138860	1.023196	-0.000179
6	6	0	2.650833	-0.275848	-0.000463
7	1	0	2.171558	-2.357201	-0.000320
8	1	0	-0.260136	-2.008236	-0.000323
9	1	0	0.359812	2.233241	0.000069
10	1	0	2.794990	1.882132	0.000242
11	8	0	-1.453032	0.471279	-0.000625
12	6	0	-2.380865	-0.584492	0.000149
13	1	0	-2.271101	-1.222523	0.885147
14	1	0	-2.271682	-1.223325	-0.884349
15	6	0	-3.809174	-0.080001	0.000386
16	8	0	-4.746330	-0.836935	0.000997
17	8	0	-3.930966	1.256655	-0.000397
18	1	0	-4.883193	1.446506	-0.000265
19	8	0	3.982790	-0.589034	-0.000469
20	6	0	4.921818	0.474107	0.001223
21	1	0	5.903751	0.005544	0.001646
22	1	0	4.820813	1.099344	-0.891724
23	1	0	4.819245	1.098066	0.894810

Optimized coordinates for radical cation 4dt$^{+\bullet}$

UB3LYP/6-311+G(2d,p)

Center Number	Atomic Number	Atomic Type	Coordinates (Angstroms) X	Y	Z
1	6	0	1.767951	-1.352438	-0.000422
2	6	0	0.393603	-1.147135	-0.000379
3	6	0	-0.115163	0.151970	-0.000347
4	6	0	0.764844	1.229098	-0.000131
5	6	0	2.138860	1.023196	-0.000179
6	6	0	2.650833	-0.275848	-0.000463
7	1	0	2.171558	-2.357201	-0.000320
8	1	0	-0.260136	-2.008236	-0.000323
9	1	0	0.359812	2.233241	0.000069
10	1	0	2.794990	1.882132	0.000242
11	8	0	-1.453032	0.471279	-0.000625
12	6	0	-2.380865	-0.584492	0.000149
13	1	0	-2.271101	-1.222523	0.885147
14	1	0	-2.271682	-1.223325	-0.884349
15	6	0	-3.809174	-0.080001	0.000386
16	8	0	-4.746330	-0.836935	0.000997
17	8	0	-3.930966	1.256655	-0.000397
18	1	0	-4.883193	1.446506	-0.000265
19	8	0	3.982790	-0.589034	-0.000469

20	6	0	4.921818	0.474107	0.001223
21	1	0	5.903751	0.005544	0.001646
22	1	0	4.820813	1.099344	-0.891724
23	1	0	4.819245	1.098066	0.894810

Optimized coordinates for radical cation 4dc$^{+\bullet}$

UB3LYP/6-311+G(2d,p)

Center Number	Atomic Number	Atomic Type	Coordinates (Angstroms) X	Y	Z
1	6	0	1.925133	-0.942322	0.000160
2	6	0	0.526093	-0.887666	-0.000051
3	6	0	-0.123594	0.338454	-0.000233
4	6	0	0.630585	1.516422	-0.000364
5	6	0	2.008848	1.461919	-0.000141
6	6	0	2.671684	0.228451	0.000199
7	1	0	2.404632	-1.910991	0.000162
8	1	0	-0.025581	-1.817543	-0.000101
9	1	0	0.111765	2.466762	-0.000648
10	1	0	2.599381	2.369544	-0.000294
11	8	0	-1.487997	0.508513	-0.000363
12	6	0	-2.290210	-0.645530	-0.000097
13	1	0	-2.107280	-1.266858	0.884700
14	1	0	-2.107580	-1.267095	-0.884780
15	6	0	-3.766526	-0.307267	0.000091
16	8	0	-4.611123	-1.166337	-0.000265
17	8	0	-4.039829	1.006648	0.000676
18	1	0	-5.007458	1.086938	0.000751
19	8	0	4.038210	0.287084	0.000254
20	6	0	4.759076	-0.934336	0.000052
21	1	0	5.812115	-0.660793	-0.000065
22	1	0	4.539798	-1.528014	0.893392
23	1	0	4.539586	-1.527960	-0.893221

Energy scan for MeO–ring dihedral UB3LYP/6-31G(d) of 4-MeOPhOCH$_2$CO$_2$H radical cation.

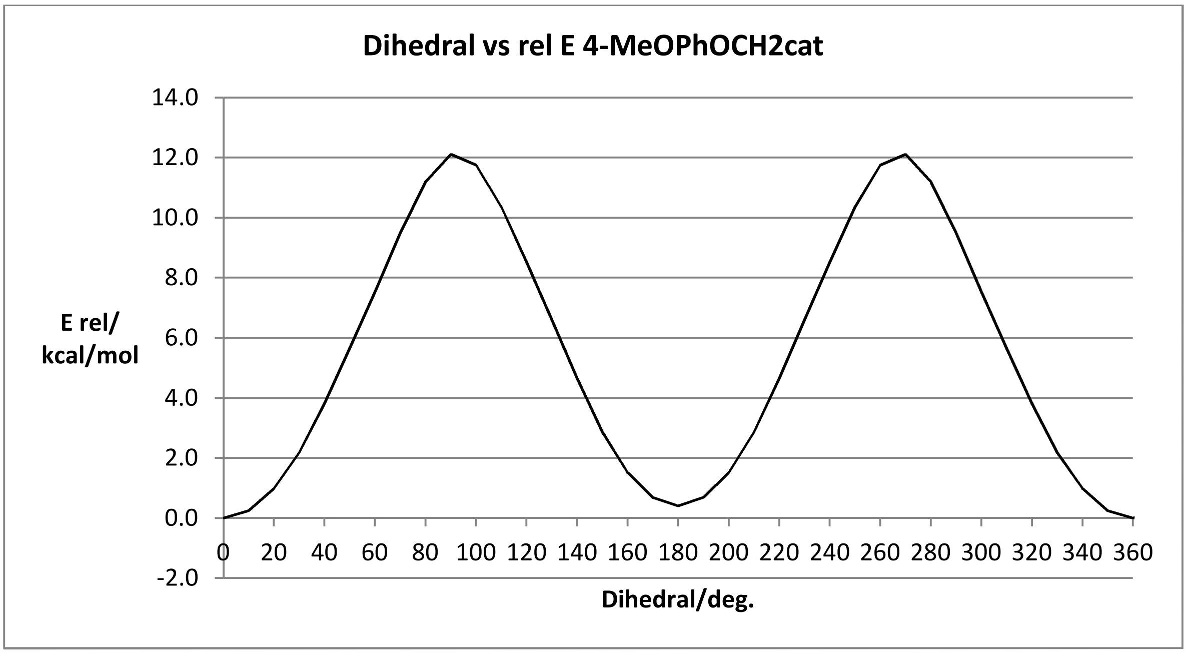

Figure S25. Relative Energy vs. dihedral angle for **4dc**$^{+\bullet}$

Optimized coordinates 3-MeOPhOCH$_2$CO$_2$H 4e

UB3LYP/6-311+G(2d,p)

Center Number	Atomic Number	Atomic Type	Coordinates (Angstroms) X	Y	Z
1	6	0	2.185627	-0.311584	-0.000041
2	6	0	0.792969	-0.465985	0.000009
3	6	0	-0.019932	0.658523	0.000026
4	6	0	0.540231	1.941332	-0.000007
5	6	0	1.915087	2.071519	-0.000067
6	6	0	2.757098	0.956429	-0.000089
7	1	0	0.398917	-1.471985	0.000044
8	1	0	-0.115283	2.801887	0.000009
9	1	0	2.354492	3.062017	-0.000104
10	8	0	-1.388344	0.615101	0.000089
11	6	0	-2.012916	-0.646644	0.000043
12	1	0	-1.740332	-1.233063	0.884780
13	1	0	-1.740312	-1.233008	-0.884724
14	6	0	-3.522744	-0.526623	0.000040
15	8	0	-4.232675	-1.499714	-0.000164
16	8	0	-3.984454	0.733520	0.000088
17	1	0	-4.953462	0.672100	0.000009
18	1	0	3.828543	1.092399	-0.000158
19	8	0	2.890225	-1.479412	-0.000094
20	6	0	4.309135	-1.410592	0.000125
21	1	0	4.682633	-0.901432	0.893473
22	1	0	4.656535	-2.441493	0.000295
23	1	0	4.682928	-0.901636	-0.893218

Optimized coordinates for radical cation of 3-MeOPhOCH$_2$CO$_2$H 4e$^{+\bullet}$

UB3LYP/6-311+G(2d,p)

Center Number	Atomic Number	Atomic Type	Coordinates (Angstroms) X	Y	Z
1	6	0	2.185305	-0.332645	0.000018
2	6	0	0.800755	-0.538953	0.000030
3	6	0	-0.030332	0.564852	0.000006
4	6	0	0.518072	1.900730	-0.000032
5	6	0	1.891569	2.091931	-0.000046
6	6	0	2.737803	1.001111	-0.000023
7	1	0	0.433168	-1.554485	0.000061
8	1	0	-0.177814	2.730268	-0.000049
9	1	0	2.297955	3.094683	-0.000077
10	8	0	-1.351204	0.544966	0.000016
11	6	0	-2.057741	-0.707703	0.000044
12	1	0	-1.795913	-1.282483	0.891194
13	1	0	-1.795877	-1.282547	-0.891053
14	6	0	-3.563277	-0.498819	0.000011
15	8	0	-4.297283	-1.445014	-0.000093
16	8	0	-3.933437	0.784722	-0.000034
17	1	0	-4.905716	0.814384	-0.000106
18	1	0	3.809303	1.141528	-0.000041
19	8	0	2.930543	-1.413352	0.000039
20	6	0	4.381023	-1.363386	0.000064
21	1	0	4.738157	-0.865627	0.900792
22	1	0	4.690526	-2.403224	0.000154
23	1	0	4.738185	-0.865775	-0.900735

Optimized coordinates for acid 2-MeOPhOCH$_2$CO$_2$H 4f

UB3LYP/6-311+G(2d,p)

Center Number	Atomic Number	Atomic Type	Coordinates (Angstroms) X	Y	Z
1	6	0	2.735775	0.363200	-0.000026
2	6	0	1.346969	0.495211	-0.000141
3	6	0	0.550613	-0.668423	0.000016

4	6	0	1.176022	-1.912424	0.000228
5	6	0	2.558761	-2.031929	0.000310
6	6	0	3.343091	-0.888135	0.000197
7	1	0	0.537742	-2.787199	0.000334
8	1	0	3.013445	-3.014618	0.000477
9	8	0	-0.819254	-0.737904	-0.000068
10	6	0	-1.661379	0.398361	0.000478
11	1	0	-1.506367	1.024111	-0.880213
12	1	0	-1.506065	1.023479	0.881568
13	6	0	-3.119172	-0.013489	0.000631
14	8	0	-4.004476	0.804735	0.000345
15	8	0	-3.336370	-1.339167	-0.000576
16	1	0	-4.300002	-1.457752	-0.000986
17	1	0	4.423628	-0.957106	0.000270
18	1	0	3.354895	1.248867	-0.000133
19	8	0	0.708370	1.709458	-0.000439
20	6	0	1.495907	2.891178	-0.000603
21	1	0	2.124248	2.952295	0.892722
22	1	0	2.124420	2.951930	-0.893833
23	1	0	0.788379	3.717726	-0.000839

Optimized coordinates for Radical cation of 2-MeOPhOCH$_2$CO$_2$H 4f$^{+\bullet}$

UB3LYP/6-311+G(2d,p)

Center Number	Atomic Number	Atomic Type	Coordinates (Angstroms) X	Y	Z
1	6	0	-2.763968	0.363580	-0.000072
2	6	0	-1.371569	0.518080	0.000078
3	6	0	-0.528527	-0.672648	0.000058
4	6	0	-1.139206	-1.949868	-0.000062
5	6	0	-2.498652	-2.062146	-0.000197
6	6	0	-3.316114	-0.897109	-0.000209
7	1	0	-0.486633	-2.813000	-0.000058
8	1	0	-2.963831	-3.039299	-0.000304
9	8	0	0.782370	-0.691195	0.000174
10	6	0	1.680400	0.443838	0.000098
11	1	0	1.511089	1.050556	0.888213
12	1	0	1.510849	1.050649	-0.887903
13	6	0	3.128031	-0.019658	-0.000120
14	8	0	4.005703	0.795448	-0.000235
15	8	0	3.282859	-1.345590	-0.000181
16	1	0	4.236938	-1.535927	-0.000325
17	1	0	-4.393424	-1.005819	-0.000322
18	1	0	-3.403202	1.234108	-0.000073
19	8	0	-0.740474	1.676390	0.000264
20	6	0	-1.481596	2.925333	0.000343
21	1	0	-2.092889	2.990852	-0.899181
22	1	0	-2.093080	2.990624	0.899753
23	1	0	-0.722281	3.700418	0.000521

Optimized coordinates for acid 3,5-Di-MeOPhOCH$_2$CO$_2$H 4g

UB3LYP/6-311+G(2d,p)

Center Number	Atomic Number	Atomic Type	Coordinates (Angstroms) X	Y	Z
1	6	0	1.674624	1.382942	-0.000222
2	6	0	0.299625	1.108613	0.000838
3	6	0	-0.104286	-0.224278	0.001426
4	6	0	0.816353	-1.270731	0.000988
5	6	0	2.174972	-0.965622	-0.000006
6	6	0	2.609851	0.358931	-0.000654
7	1	0	-0.420082	1.910134	0.001499
8	1	0	0.442887	-2.283222	0.001415
9	8	0	-1.418007	-0.619093	0.002709
10	6	0	-2.413973	0.374197	0.000680
11	1	0	-2.344842	1.015802	-0.885146
12	1	0	-2.347019	1.017478	0.885419

13	6	0	-3.805932	-0.223175	-0.000402
14	8	0	-4.789684	0.472049	0.000372
15	8	0	-3.840631	-1.564650	-0.002686
16	1	0	-4.778479	-1.816228	-0.003357
17	8	0	3.161323	-1.905381	-0.000545
18	8	0	2.186205	2.647644	-0.000757
19	6	0	2.790329	-3.276796	-0.000198
20	1	0	2.212450	-3.533134	-0.892997
21	1	0	3.723297	-3.836344	-0.000927
22	1	0	2.213892	-3.533051	0.893572
23	6	0	1.291578	3.749661	-0.000602
24	1	0	0.661068	3.753608	0.893812
25	1	0	1.917500	4.639494	-0.001350
26	1	0	0.659981	3.752914	-0.894286
27	1	0	3.666863	0.585544	-0.001473

Optimized coordinates for radical cation 3,5-Di-MeOPhOCH$_2$CO$_2$H 4g$^{+\bullet}$

UB3LYP/6-311+G(2d,p)

Center Number	Atomic Number	Atomic Type	Coordinates (Angstroms) X	Y	Z
1	6	0	1.676943	1.383432	-0.000234
2	6	0	0.311076	1.170500	0.000565
3	6	0	-0.135165	-0.163908	0.000969
4	6	0	0.755844	-1.280270	0.000709
5	6	0	2.114938	-1.058182	-0.000147
6	6	0	2.590783	0.276789	-0.000637
7	1	0	-0.385550	1.992042	0.000976
8	1	0	0.317097	-2.266813	0.001215
9	8	0	-1.406146	-0.514790	0.001776
10	6	0	-2.456518	0.468798	0.001399
11	1	0	-2.382277	1.094124	-0.890159
12	1	0	-2.383961	1.092790	0.894048
13	6	0	-3.829917	-0.183176	-0.000420
14	8	0	-4.813162	0.500713	-0.001003
15	8	0	-3.800023	-1.518385	-0.001213
16	1	0	-4.719381	-1.836280	-0.002326
17	8	0	3.068669	-1.986575	-0.000561
18	8	0	2.282992	2.557615	-0.000587
19	6	0	2.692039	-3.375704	-0.000109
20	1	0	2.117073	-3.612827	-0.897339
21	1	0	3.626910	-3.927021	-0.001084
22	1	0	2.118977	-3.612802	0.898350
23	6	0	1.506667	3.773611	-0.000271
24	1	0	0.891166	3.823366	0.899177
25	1	0	2.235776	4.577201	-0.000972
26	1	0	0.889832	3.823008	-0.898836
27	1	0	3.655540	0.473246	-0.001290

Optimized coordinates for acid 3,4,5-Tri-MeOPhOCH$_2$CO$_2$H 4h

UB3LYP/6-311+G(2d,p)

Center Number	Atomic Number	Atomic Type	Coordinates (Angstroms) X	Y	Z
1	6	0	-1.275311	-1.088471	-0.000108
2	6	0	0.122593	-0.955479	0.000052
3	6	0	0.693700	0.307394	0.000181
4	6	0	-0.105484	1.445603	0.000157
5	6	0	-1.486665	1.310853	0.000022
6	6	0	-2.104339	0.038643	-0.000104
7	1	0	0.735477	-1.841021	0.000079
8	1	0	0.373046	2.412804	0.000248
9	8	0	2.049301	0.544401	0.000358
10	6	0	2.910845	-0.564683	0.000079
11	1	0	2.762567	-1.194684	-0.885151
12	1	0	2.762726	-1.195025	0.885091
13	6	0	4.367239	-0.149046	0.000033

14	8	0	5.256024	-0.962581	0.000030
15	8	0	4.571860	1.177338	-0.000013
16	1	0	5.534060	1.307395	-0.000045
17	8	0	-3.468132	0.085729	-0.000249
18	6	0	-4.298134	-1.072837	0.000092
19	1	0	-5.314438	-0.680795	0.000093
20	1	0	-4.141606	-1.683148	-0.889723
21	1	0	-4.141447	-1.682723	0.890177
22	8	0	-2.344346	2.364835	-0.000007
23	8	0	-1.882590	-2.319194	-0.000296
24	6	0	-1.800819	3.675543	0.000114
25	1	0	-1.195309	3.857876	-0.892969
26	1	0	-2.655560	4.348701	0.000072
27	1	0	-1.195473	3.857779	0.893328
28	6	0	-1.076284	-3.485370	-0.000319
29	1	0	-0.447410	-3.540419	0.893631
30	1	0	-1.770419	-4.323634	-0.000493
31	1	0	-0.447189	-3.540229	-0.894126

Optimized coordinates for radical cation 3,4,5-Tri-MeOPhOCH$_2$CO$_2$H 4h$^{+\bullet}$

UB3LYP/6-311+G(2d,p)

Center Number	Atomic Number	Atomic Type	Coordinates (Angstroms) X	Y	Z
1	6	0	-1.264568	-1.127536	-0.000001
2	6	0	0.110623	-1.012850	0.000187
3	6	0	0.702970	0.265346	0.000326
4	6	0	-0.069237	1.444225	0.000304
5	6	0	-1.438242	1.359801	0.000119
6	6	0	-2.087095	0.059709	-0.000039
7	1	0	0.722755	-1.899238	0.000282
8	1	0	0.450263	2.390258	0.000411
9	8	0	2.014717	0.469312	0.000533
10	6	0	2.933218	-0.630464	0.000218
11	1	0	2.790871	-1.245208	-0.891116
12	1	0	2.791467	-1.245202	0.891655
13	6	0	4.375943	-0.152234	-0.000279
14	8	0	5.272059	-0.948349	-0.000220
15	8	0	4.511957	1.177053	-0.000480
16	1	0	5.463947	1.375872	-0.000679
17	8	0	-3.392552	0.115166	-0.000219
18	6	0	-4.322514	-1.001688	-0.000284
19	1	0	-5.297466	-0.524908	-0.000326
20	1	0	-4.181886	-1.601725	-0.895247
21	1	0	-4.181975	-1.601754	0.894673
22	8	0	-2.281082	2.382375	0.000061
23	8	0	-1.927607	-2.284861	-0.000142
24	6	0	-1.757630	3.723158	0.000222
25	1	0	-1.161765	3.897701	-0.897147
26	1	0	-2.629692	4.369390	0.000157
27	1	0	-1.161991	3.897574	0.897766
28	6	0	-1.195250	-3.525561	-0.000147
29	1	0	-0.581181	-3.602833	0.898325
30	1	0	-1.951678	-4.304262	-0.000333
31	1	0	-0.580908	-3.602666	-0.898447

Optimized coordinates for acid PhSCH$_2$CO$_2$H 4i

UB3LYP/6-311+G(2d,p)

Center Number	Atomic Number	Atomic Type	Coordinates (Angstroms) X	Y	Z
1	6	0	-2.766884	1.475332	0.000037
2	6	0	-1.429016	1.086639	0.000068
3	6	0	-1.095623	-0.268365	-0.000007
4	6	0	-2.121239	-1.221680	-0.000112
5	6	0	-3.449395	-0.824577	-0.000140
6	6	0	-3.782013	0.528409	-0.000067

7	1	0	-3.008949	2.531709	0.000095
8	1	0	-0.665336	1.851550	0.000152
9	1	0	-1.873695	-2.277199	-0.000172
10	1	0	-4.229245	-1.576953	-0.000221
11	6	0	1.584340	0.587217	0.000093
12	1	0	1.371833	1.192897	0.882350
13	1	0	1.371827	1.192981	-0.882103
14	6	0	3.076411	0.328425	0.000082
15	8	0	3.886412	1.218132	-0.000197
16	8	0	3.412942	-0.975355	0.000169
17	1	0	4.382692	-1.020586	0.000063
18	1	0	-4.819896	0.837440	-0.000091
19	16	0	0.561028	-0.926403	0.000027

Optimized coordinates for radical cation of $PhSCH_2CO_2H$ 4i$^{+\bullet}$

UB3LYP/6-311+G(2d,p)

Center Number	Atomic Number	Atomic Type	Coordinates (Angstroms) X	Y	Z
1	6	0	-2.787166	1.469184	0.000020
2	6	0	-1.452473	1.140798	0.000040
3	6	0	-1.077338	-0.225522	-0.000018
4	6	0	-2.077085	-1.242540	-0.000096
5	6	0	-3.406565	-0.892211	-0.000114
6	6	0	-3.768759	0.460944	-0.000057
7	1	0	-3.086105	2.509545	0.000065
8	1	0	-0.707155	1.922837	0.000101
9	1	0	-1.780941	-2.284879	-0.000140
10	1	0	-4.169455	-1.659702	-0.000173
11	6	0	1.617861	0.647150	0.000089
12	1	0	1.413264	1.245006	0.889168
13	1	0	1.413245	1.245140	-0.888894
14	6	0	3.102442	0.288567	0.000047
15	8	0	3.939837	1.138137	-0.000006
16	8	0	3.316253	-1.035169	0.000067
17	1	0	4.275077	-1.202405	0.000039
18	1	0	-4.816160	0.736780	-0.000071
19	16	0	0.531500	-0.825892	-0.000003

Optimized coordinates for acid 4-MeO-$PhSCH_2CO_2H$ 4j

UB3LYP/6-311+G(2d,p)

Center Number	Atomic Number	Atomic Type	Coordinates (Angstroms) X	Y	Z
1	6	0	2.087168	-0.952837	0.000072
2	6	0	0.707355	-0.732132	-0.000053
3	6	0	0.189849	0.557817	-0.000248
4	6	0	1.087077	1.635944	-0.000325
5	6	0	2.450967	1.424460	-0.000216
6	6	0	2.966619	0.123895	-0.000011
7	1	0	2.449814	-1.971158	0.000226
8	1	0	0.058423	-1.596812	-0.000001
9	1	0	0.708021	2.651659	-0.000463
10	1	0	3.141296	2.258759	-0.000273
11	6	0	-2.342450	-0.651036	0.000157
12	1	0	-2.045002	-1.220280	0.882355
13	1	0	-2.045023	-1.220987	-0.881571
14	6	0	-3.855662	-0.609237	0.000138
15	8	0	-4.530159	-1.606013	-0.000703
16	8	0	-4.376811	0.632381	0.001287
17	1	0	-5.342857	0.536692	0.001214
18	8	0	4.327946	0.022136	0.000092
19	6	0	4.903937	-1.275215	0.000318
20	1	0	5.981195	-1.123604	0.000394
21	1	0	4.617611	-1.838943	0.893768
22	1	0	4.617771	-1.839184	-0.893031
23	16	0	-1.542389	0.990367	-0.000439

Optimized coordinates for radical cation 4-MeO-PhSCH$_2$CO$_2$H 4j$^{+•}$

UB3LYP/6-311+G(2d,p)

Center Number	Atomic Number	Atomic Type	Coordinates (Angstroms) X	Y	Z
1	6	0	2.082972	-0.989677	0.000065
2	6	0	0.725076	-0.800426	0.000061
3	6	0	0.179271	0.507131	-0.000085
4	6	0	1.061029	1.628112	-0.000230
5	6	0	2.410446	1.445449	-0.000226
6	6	0	2.950928	0.131359	-0.000077
7	1	0	2.481538	-1.994035	0.000175
8	1	0	0.079213	-1.666857	0.000168
9	1	0	0.650209	2.630591	-0.000343
10	1	0	3.096016	2.282814	-0.000333
11	6	0	-2.367601	-0.709938	0.000116
12	1	0	-2.088353	-1.275680	0.889142
13	1	0	-2.088437	-1.275899	-0.888794
14	6	0	-3.886034	-0.563300	0.000175
15	8	0	-4.600162	-1.520739	0.000000
16	8	0	-4.288872	0.716154	0.000336
17	1	0	-5.261473	0.738644	0.000332
18	8	0	4.264685	0.059728	-0.000087
19	6	0	4.951201	-1.213716	0.000054
20	1	0	6.006697	-0.962258	0.000023
21	1	0	4.698581	-1.775117	0.899584
22	1	0	4.698576	-1.775318	-0.899350
23	16	0	-1.495093	0.901376	-0.000107

Optimized coordinates for *transoid* acid 4-MeS-PhOCH$_2$CO$_2$H 4kt

UB3LYP/6-311+G(2d,p)

Center Number	Atomic Number	Atomic Type	Coordinates (Angstroms) X	Y	Z
1	6	0	-1.379862	-1.332161	-0.000052
2	6	0	-0.003278	-1.133707	-0.000057
3	6	0	0.509434	0.162799	0.000008
4	6	0	-0.368448	1.244349	0.000078
5	6	0	-1.739849	1.038712	0.000081
6	6	0	-2.266403	-0.255917	0.000016
7	1	0	-1.762935	-2.346210	-0.000100
8	1	0	0.646570	-1.997732	-0.000110
9	1	0	0.039739	2.247304	0.000127
10	1	0	-2.390987	1.901995	0.000133
11	6	0	2.773067	-0.579453	-0.000068
12	1	0	2.662221	-1.216721	-0.885066
13	1	0	2.662262	-1.216819	0.884864
14	6	0	4.201429	-0.074855	-0.000073
15	8	0	5.137702	-0.832531	-0.000079
16	8	0	4.322670	1.261570	-0.000092
17	1	0	5.274689	1.452598	-0.000104
18	6	0	-4.808646	0.986757	0.000191
19	1	0	-5.878158	0.779335	0.000199
20	1	0	-4.558293	1.554776	0.895789
21	1	0	-4.558343	1.554958	-0.895304
22	8	0	1.844776	0.477950	0.000010
23	16	0	-4.005163	-0.644284	0.000007

Optimized coordinates for *transoid* radical cation 4-MeS-PhOCH$_2$CO$_2$H 4kt$^{+•}$

UB3LYP/6-311+G(2d,p)

Center Number	Atomic Number	Atomic Type	Coordinates (Angstroms) X	Y	Z
1	6	0	1.385858	-1.357814	0.000081
2	6	0	0.027150	-1.189865	0.000100
3	6	0	-0.507866	0.119989	-0.000019
4	6	0	0.362027	1.244107	-0.000156
5	6	0	1.716301	1.071322	-0.000175
6	6	0	2.262621	-0.236905	-0.000056
7	1	0	1.801820	-2.358327	0.000170
8	1	0	-0.618545	-2.056252	0.000201
9	1	0	-0.082132	2.231165	-0.000246
10	1	0	2.361387	1.938331	-0.000289
11	6	0	-2.793602	-0.624262	0.000115
12	1	0	-2.689633	-1.245527	0.891724
13	1	0	-2.689677	-1.245711	-0.891370
14	6	0	-4.197726	-0.041841	0.000090
15	8	0	-5.147070	-0.772276	0.000097
16	8	0	-4.234031	1.293528	0.000087
17	1	0	-5.168077	1.564866	0.000085
18	6	0	4.818777	0.973996	-0.000061
19	1	0	5.874407	0.707094	-0.000569
20	1	0	4.584746	1.539504	-0.900486
21	1	0	4.585527	1.539108	0.900812
22	8	0	-1.794931	0.411884	-0.000014
23	16	0	3.937950	-0.614482	-0.000056

Optimized coordinates for *cisoid* acid 4-MeS-PhOCH$_2$CO$_2$H 4kc

UB3LYP/6-311+G(2d,p)

Center Number	Atomic Number	Atomic Type	Coordinates (Angstroms) X	Y	Z
1	6	0	1.545700	-0.893620	0.000110
2	6	0	0.148604	-0.863366	0.000090
3	6	0	-0.519334	0.355131	-0.000039
4	6	0	0.218165	1.542389	-0.000157
5	6	0	1.598089	1.504664	-0.000146
6	6	0	2.286037	0.282249	-0.000008
7	1	0	2.034913	-1.857897	0.000221
8	1	0	-0.389572	-1.801197	0.000178
9	1	0	-0.312511	2.486161	-0.000263
10	1	0	2.154215	2.435118	-0.000247
11	6	0	-2.672002	-0.660324	0.000082
12	1	0	-2.481416	-1.278432	0.885109
13	1	0	-2.481459	-1.278638	-0.884805
14	6	0	-4.152232	-0.338904	0.000093
15	8	0	-4.985939	-1.208267	-0.000201
16	8	0	-4.440128	0.971609	0.000214
17	1	0	-5.408575	1.041814	0.000122
18	6	0	4.578437	-1.373671	-0.000042
19	1	0	5.667710	-1.352306	-0.000107
20	1	0	4.234654	-1.890942	0.895478
21	1	0	4.234542	-1.890898	-0.895544
22	8	0	-1.883076	0.504535	-0.000066
23	16	0	4.064492	0.369807	0.000024

Optimized coordinates for *cisoid* radical cation 4-MeS-PhOCH$_2$CO$_2$H 4kc$^{+\bullet}$

UB3LYP/6-311+G(2d,p)

Center Number	Atomic Number	Atomic Type	Coordinates (Angstroms) X	Y	Z
1	6	0	1.544866	-0.942122	0.000045
2	6	0	0.173479	-0.927575	0.000071
3	6	0	-0.515701	0.310170	0.000011
4	6	0	0.212191	1.529248	-0.000077
5	6	0	1.574237	1.509761	-0.000104
6	6	0	2.277493	0.269862	-0.000044

7	1	0	2.058266	-1.893210	0.000096
8	1	0	-0.368697	-1.862453	0.000140
9	1	0	-0.342749	2.458318	-0.000122
10	1	0	2.129830	2.439762	-0.000171
11	6	0	-2.692427	-0.711614	0.000107
12	1	0	-2.513075	-1.315222	0.891838
13	1	0	-2.513090	-1.315337	-0.891549
14	6	0	-4.157857	-0.306691	0.000100
15	8	0	-5.009572	-1.149034	-0.000116
16	8	0	-4.358018	1.013679	0.000145
17	1	0	-5.318203	1.169067	0.000060
18	6	0	4.626787	-1.311348	-0.000006
19	1	0	5.710192	-1.202112	-0.000114
20	1	0	4.313873	-1.836293	0.900942
21	1	0	4.313710	-1.836446	-0.900806
22	8	0	-1.828961	0.439427	0.000030
23	16	0	3.990246	0.390200	-0.000088

Optimized coordinates for acid $PhNHCH_2CO_2H$ 4l

UB3LYP/aug-cc-pvtz

Center Number	Atomic Number	Atomic Type	Coordinates (Angstroms) X	Y	Z
1	6	0	3.076646	1.073740	0.016759
2	6	0	1.710074	1.270341	-0.075327
3	6	0	0.827916	0.178124	-0.097868
4	6	0	1.365544	-1.113070	-0.021819
5	6	0	2.741925	-1.296103	0.066815
6	6	0	3.608281	-0.212622	0.087646
7	1	0	3.734382	1.932781	0.033297
8	1	0	1.310621	2.275358	-0.136386
9	1	0	0.719392	-1.978630	-0.037078
10	1	0	3.133927	-2.303157	0.122143
11	1	0	4.676196	-0.362639	0.158291
12	7	0	-0.535303	0.409427	-0.227896
13	1	0	-0.841503	1.340292	-0.002158
14	6	0	-1.504061	-0.626661	0.027683
15	1	0	-1.357153	-1.116121	1.003130
16	1	0	-1.445665	-1.417560	-0.722010
17	6	0	-2.937283	-0.144336	0.021824
18	8	0	-3.887916	-0.873141	-0.071451
19	8	0	-3.051991	1.196042	0.176278
20	1	0	-3.998079	1.403998	0.183152

Optimized coordinates for cation $PhNHCH_2CO_2H$ $4l^{+\bullet}$

UB3LYP/aug-cc-pvtz

Center Number	Atomic Number	Atomic Type	Coordinates (Angstroms) X	Y	Z
1	6	0	-3.030550	1.126637	-0.000089
2	6	0	-1.671153	1.286872	0.000058
3	6	0	-0.818271	0.137660	0.000108
4	6	0	-1.392081	-1.168483	-0.000010
5	6	0	-2.755602	-1.301518	-0.000164
6	6	0	-3.584848	-0.164286	-0.000187
7	1	0	-3.678121	1.991381	-0.000138
8	1	0	-1.226951	2.273570	0.000115
9	1	0	-0.758403	-2.042524	0.000022
10	1	0	-3.199718	-2.286529	-0.000286
11	1	0	-4.658836	-0.286667	-0.000270
12	7	0	0.504903	0.329915	0.000336
13	1	0	0.851130	1.283860	0.000470
14	6	0	1.539326	-0.695488	0.000511
15	1	0	1.444801	-1.333810	-0.880202

16	1	0	1.445234	-1.333044	0.881840
17	6	0	2.950456	-0.121896	-0.000079
18	8	0	3.918137	-0.817060	-0.000374
19	8	0	2.951094	1.227439	-0.000163
20	1	0	3.869033	1.544342	-0.000500

DFT Optimised Energies

Acid $PhOCH_2CO_2H$ (4a).

UB3LYP/6-311+G(2d,p): E = -535.50894310 AU

UB3LYP/aug-cc-pvtz: E = -535.5474946

UB3LYP/aug-cc-pvtz CPCM (solvent = acetonitrile): E = -535.5577593

Cation $PhOCH_2CO_2H$ ($4a^{+\bullet}$).

UB3LYP/6-311+G(2d,p): E = -535.20722747 AU

UB3LYP/aug-cc-pvtz: E = -535.2455421

UB3LYP/aug-cc-pvtz CPCM (solvent = acetonitrile): E = -535.3267171

Acid 4-$MePhOCH_2CO_2H$ (4b).

UB3LYP/6-311+G(2d,p): E = -574.83709304 AU

UB3LYP/aug-cc-pvtz: E = -574.878329

UB3LYP/aug-cc-pvtz CPCM (solvent = acetonitrile): E = -574.8885358

Cation 4-$MePhOCH_2CO_2H$ ($4b^{+\bullet}$).

UB3LYP/6-311+G(2d,p): E = -574.54764345 AU

UB3LYP/aug-cc-pvtz: E = -574.5888231

UB3LYP/aug-cc-pvtz CPCM (solvent = acetonitrile): E = -574.6669601

Acid 4-t-$BuPhOCH_2CO_2H$ (4c).

UB3LYP/6-311+G(2d,p): E = -692.80775208 AU

UB3LYP/aug-cc-pvtz: E = -692.8575113

UB3LYP/aug-cc-pvtz CPCM (solvent = acetonitrile): E = -692.8678447

Cation 4-t-$BuPhOCH_2CO_2H$ ($4c^{+\bullet}$).

UB3LYP/6-311+G(2d,p): E = -692.52231987 AU

UB3LYP/aug-cc-pvtz: E = -692.5720663

UB3LYP/aug-cc-pvtz CPCM (solvent = acetonitrile): E = -692.646069

Acid 4-MeOPhOCH$_2$CO$_2$H *cis* (4d$_c$).

UB3LYP/6-311+G(2d,p): E = -650.06645741 AU

UB3LYP/aug-cc-pvtz: E = -650.1132596

UB3LYP/aug-cc-pvtz CPCM (solvent = acetonitrile): E = -650.1251373

Cation 4-MeOPhOCH$_2$CO$_2$H *cis* (4d$_c^{+\bullet}$).

UB3LYP/6-311+G(2d,p): E = -650.06645741 AU

UB3LYP/aug-cc-pvtz: E = -649.8396988

UB3LYP/aug-cc-pvtz CPCM (solvent = acetonitrile): E = -649.9169588

Acid 4-MeOPhOCH$_2$CO$_2$H *trans* (4d$_t$).

UB3LYP/6-311+G(2d,p): E = -650.06606387 AU

UB3LYP/aug-cc-pvtz: E = -650.1128708

UB3LYP/aug-cc-pvtz CPCM (solvent = acetonitrile): E = -649.8403027

Cation 4-MeOPhOCH$_2$CO$_2$H *trans* (4d$_t^{+\bullet}$).

UB3LYP/6-311+G(2d,p): E = -650.06606387 AU

UB3LYP/aug-cc-pvtz: E = -650.1247972

UB3LYP/aug-cc-pvtz CPCM (solvent = acetonitrile): E = -649.9172627

Acid 3-MeOPhOCH$_2$CO$_2$H (4e).

UB3LYP/6-311+G(2d,p): E = -650.06917168 AU

UB3LYP/aug-cc-pvtz: E = -650.1160242

UB3LYP/aug-cc-pvtz CPCM (solvent = acetonitrile): E = -650.1277081

Cation 3-MeOPhOCH$_2$CO$_2$H (4e$^{+\bullet}$).

UB3LYP/6-311+G(2d,p): E = -649.78265669 AU

UB3LYP/aug-cc-pvtz: E = -649.8293864

UB3LYP/aug-cc-pvtz CPCM (solvent = acetonitrile): E = -649.9068673

Acid 2-MeOPhOCH$_2$CO$_2$H (4f).

UB3LYP/6-311+G(2d,p): E = -650.05904354 AU

UB3LYP/aug-cc-pvtz: E = -650.1057583

UB3LYP/aug-cc-pvtz CPCM (solvent = acetonitrile): E = -650.116515

Cation 2-MeOPhOCH$_2$CO$_2$H (4f$^{+\bullet}$).

UB3LYP/6-311+G(2d,p): E = -649.78110303 AU

UB3LYP/aug-cc-pvtz: E = -649.8277148

UB3LYP/aug-cc-pvtz CPCM (solvent = acetonitrile): E = -649.9046441

Acid 3,5-DiMeOPhOCH$_2$CO$_2$H (4g).

UB3LYP/6-311+G(2d,p): E = -764.62806667 AU

UB3LYP/aug-cc-pvtz: E = -764.68494407 AU

UB3LYP/aug-cc-pvtz CPCM (solvent = acetonitrile): E = -764.6978636 AU

Cation 3,5-DiMeOPhOCH$_2$CO$_2$H (4g$^{+\bullet}$).

UB3LYP/6-311+G(2d,p): E = -764.35033790 AU

UB3LYP/aug-cc-pvtz: E = -764.4023231 AU

UB3LYP/aug-cc-pvtz CPCM (solvent = acetonitrile): E = -764.4771956

Acid 3,4,5-TriMeOPhOCH$_2$CO$_2$H *cis* (4h$_c$).

UB3LYP/6-311+G(2d,p): E = -878.907662

UB3LYP/aug-cc-pvtz: E = -879.2317218 AU

UB3LYP/aug-cc-pvtz CPCM (solvent = acetonitrile): E = -879.2469809

Cation 3,4,5-TriMeO-PhOCH$_2$CO$_2$H *cis* (4h$_c^{+\bullet}$).

UB3LYP/6-311+G(2d,p): E = -878.659239

UB3LYP/aug-cc-pvtz: E = -878.9853928 AU

UB3LYP/aug-cc-pvtz CPCM (solvent = acetonitrile): E = -879.0569931 AU

Acid 3,4,5-TriMeOPhOCH$_2$CO$_2$H *trans* (4h$_t$).

UB3LYP/6-311+G(2d,p): E = -879.16840878 AU

UB3LYP/aug-cc-pvtz: E = -879.2375054 AU

UB3LYP/aug-cc-pvtz CPCM (solvent = acetonitrile): E = -879.2536305 AU

Cation 3,4,5-TriMeO-PhOCH$_2$CO$_2$H *trans* (4h$_t^{+\bullet}$).

UB3LYP/6-311+G(2d,p): E = -878.92198901 AU

UB3LYP/aug-cc-pvtz: E = -878.9861185 AU

UB3LYP/aug-cc-pvtz CPCM (solvent = acetonitrile): E = -879.0571986 AU

Acid PhSCH$_2$CO$_2$H (4i).

UB3LYP/6-311+G(2d,p): E = -858.48749778 AU

UB3LYP/aug-cc-pvtz: E = -858.5270246

UB3LYP/aug-cc-pvtz CPCM (solvent = acetonitrile): E = -858.537174

Cation PhSCH$_2$CO$_2$H (4i$^{+\bullet}$).

UB3LYP/6-311+G(2d,p): E = -858.19958129 AU

UB3LYP/aug-cc-pvtz: E = -858.2388601

UB3LYP/aug-cc-pvtz CPCM (solvent = acetonitrile): E = -858.3171062

Acid 4-MeOPhSCH$_2$CO$_2$H *cis* (4j$_c$).

UB3LYP/aug-cc-pvtz: E = -973.096988 AU

UB3LYP/aug-cc-pvtz CPCM (solvent = acetonitrile): E = -973.1093324 AU

Cation 4-MeOPhSCH$_2$CO$_2$H *cis* (4j$_c^{+\bullet}$).

UB3LYP/aug-cc-pvtz: E = -972.8298526 AU

UB3LYP/aug-cc-pvtz CPCM (solvent = acetonitrile): E = -972.9061419 AU

Acid 4-MeOPhSCH$_2$CO$_2$H *trans* (4j$_t$).

UB3LYP/6-311+G(2d,p): E = -973.04601836 AU

UB3LYP/aug-cc-pvtz: E = -973.09362539 AU

UB3LYP/aug-cc-pvtz CPCM (solvent = acetonitrile): E = -973.10554888 AU

Cation 4-MeOPhSCH$_2$CO$_2$H *trans* (4j$_t^{+\bullet}$).

UB3LYP/6-311+G(2d,p): E = -972.77988485 AU

UB3LYP/aug-cc-pvtz: E = -972.82811764 AU

UB3LYP/aug-cc-pvtz CPCM (solvent = acetonitrile): E = -972.90225631 AU

Acid 4-MeSPhOCH$_2$CO$_2$H *cis* (4k$_c$).

UB3LYP/6-311+G(2d,p): E = -973.04915177 AU

UB3LYP/aug-cc-pvtz: E = -973.096988 AU

UB3LYP/aug-cc-pvtz CPCM (solvent = acetonitrile): E = -973.1093324 AU

Cation 4-MeSPhOCH$_2$CO$_2$H *cis* (4k$_c^{+\bullet}$).

UB3LYP/6-311+G(2d,p): E = -972.78213124 AU

UB3LYP/aug-cc-pvtz: E = -972.8298535 AU

UB3LYP/aug-cc-pvtz CPCM (solvent = acetonitrile): E = -972.9061423 AU

Acid 4-MeSPhOCH$_2$CO$_2$H *trans* (4k$_t$).

UB3LYP/6-311+G(2d,p): E = -973.04890294 AU

UB3LYP/aug-cc-pvtz: E = -973.0967375 AU

UB3LYP/aug-cc-pvtz CPCM (solvent = acetonitrile): E = -973.1090995 AU

Cation 4-MeSPhOCH$_2$CO$_2$H *trans* (4k$_t^{+\bullet}$).

UB3LYP/6-311+G(2d,p): E = -972.78259053 AU

UB3LYP/aug-cc-pvtz: E = -972.8303133 AU

UB3LYP/aug-cc-pvtz CPCM (solvent = acetonitrile): E = -972.9061423 AU

Acid $PhNHCH_2CO_2H$ (4l).

UB3LYP/aug-cc-pvtz: E = -515.6816366 AU

UB3LYP/aug-cc-pvtz CPCM (solvent = acetonitrile): E = -515.6915143 AU

Cation $PhNHCH_2CO_2H$ ($4l^{+\bullet}$).

UB3LYP/aug-cc-pvtz: E = -515.4099793 AU

UB3LYP/aug-cc-pvtz CPCM (solvent = acetonitrile): E = -515.4898162 AU

References

1. Morimoto, M.; Fukumoto, H.; Nozoe T.; Hagiwara, A.; Komai, K., *J. Agric. Food Chem.* **2007**, 55, 700-705

2. Cabiddu, S.; Fattuoni, C.; Floris, C.; Gelli, G.; Melis, S., *Tetrahedron* **1993** 49, 4965-4974.

3. Manley, D. W.; McBurney, R. T.; Miller, P.; Howe, R.l F.; Rhydderch, S.; Walton, J. C., *J. Am. Chem. Soc.* **2012**, 134(33), 13580-13583.

^{1}H and ^{13}C NMR spectra of novel compounds

4-(methylthio)phenoxyacetic acid 4k

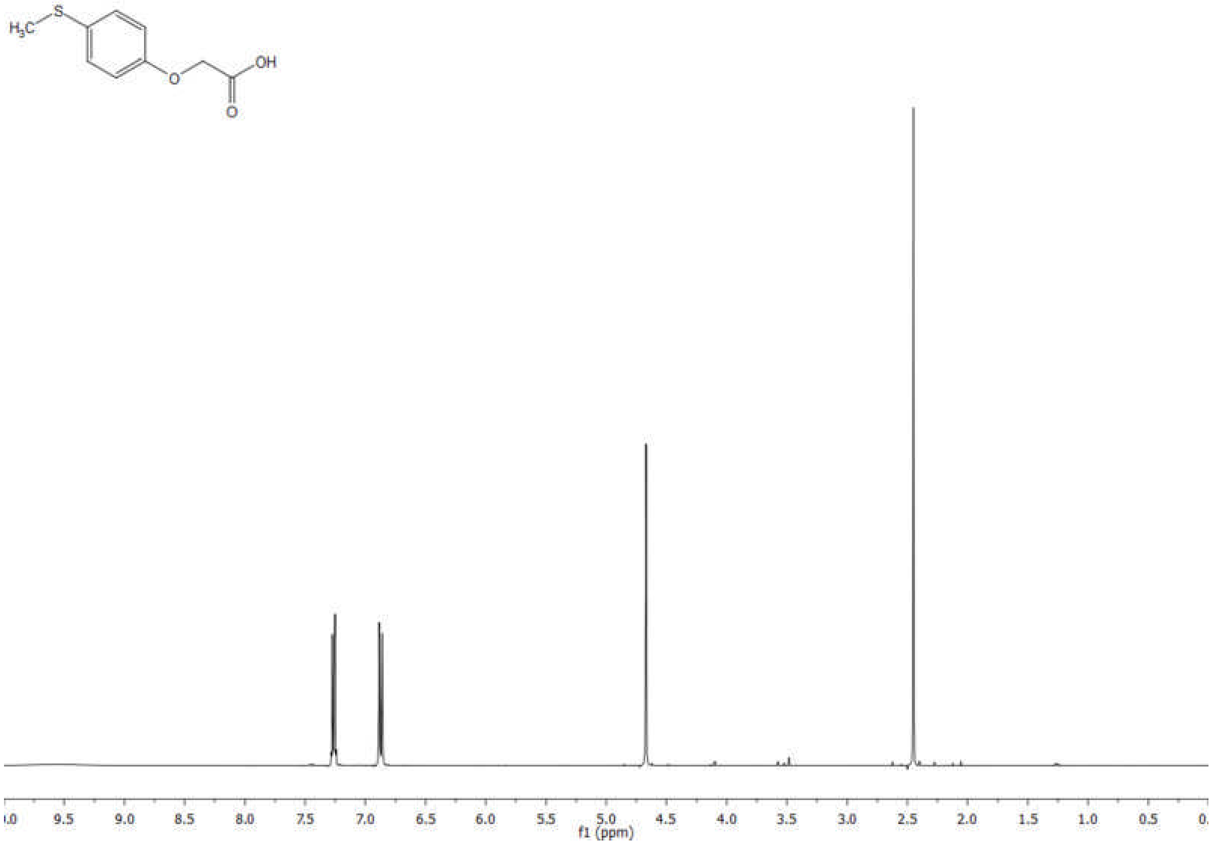

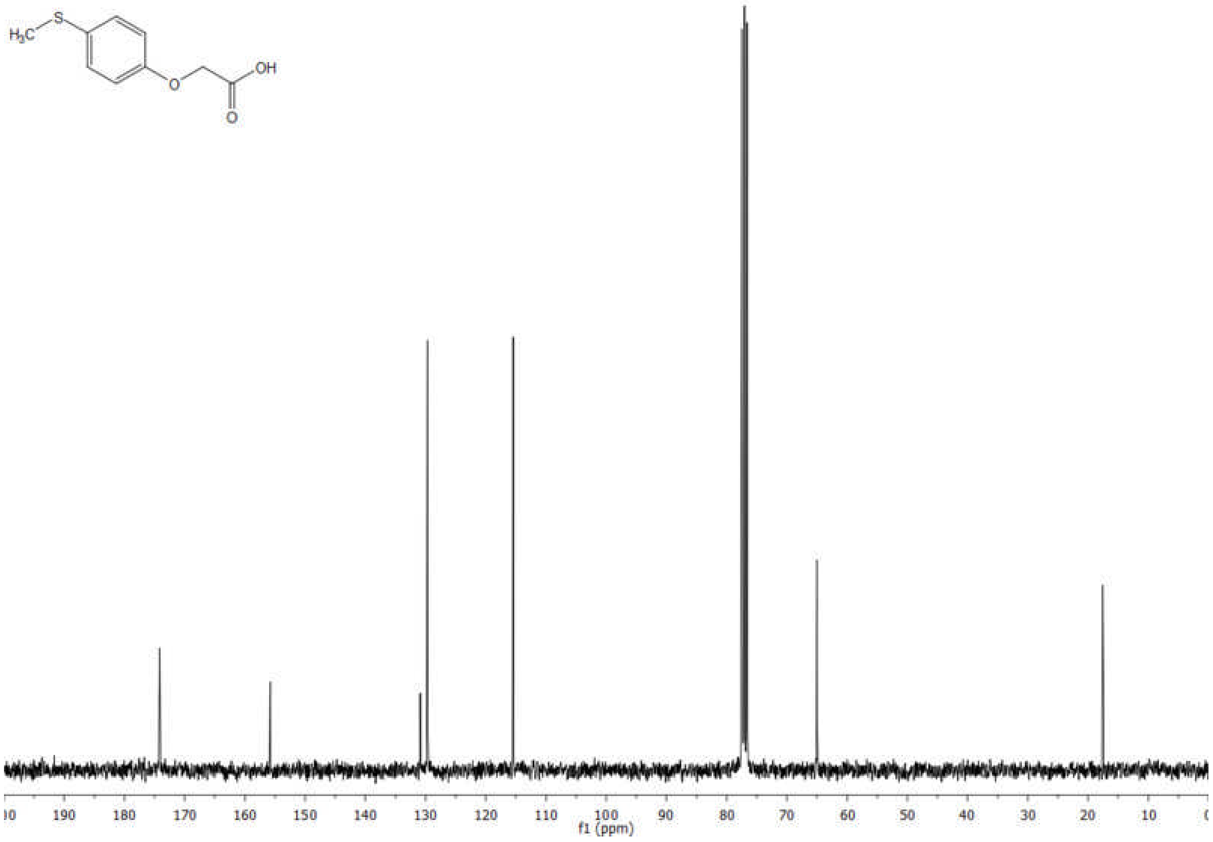

8-methyl-3a,4-dihydrochromeno[3,4-*c*]pyrrole-1,3(2*H*,9b*H*)-dione 5b

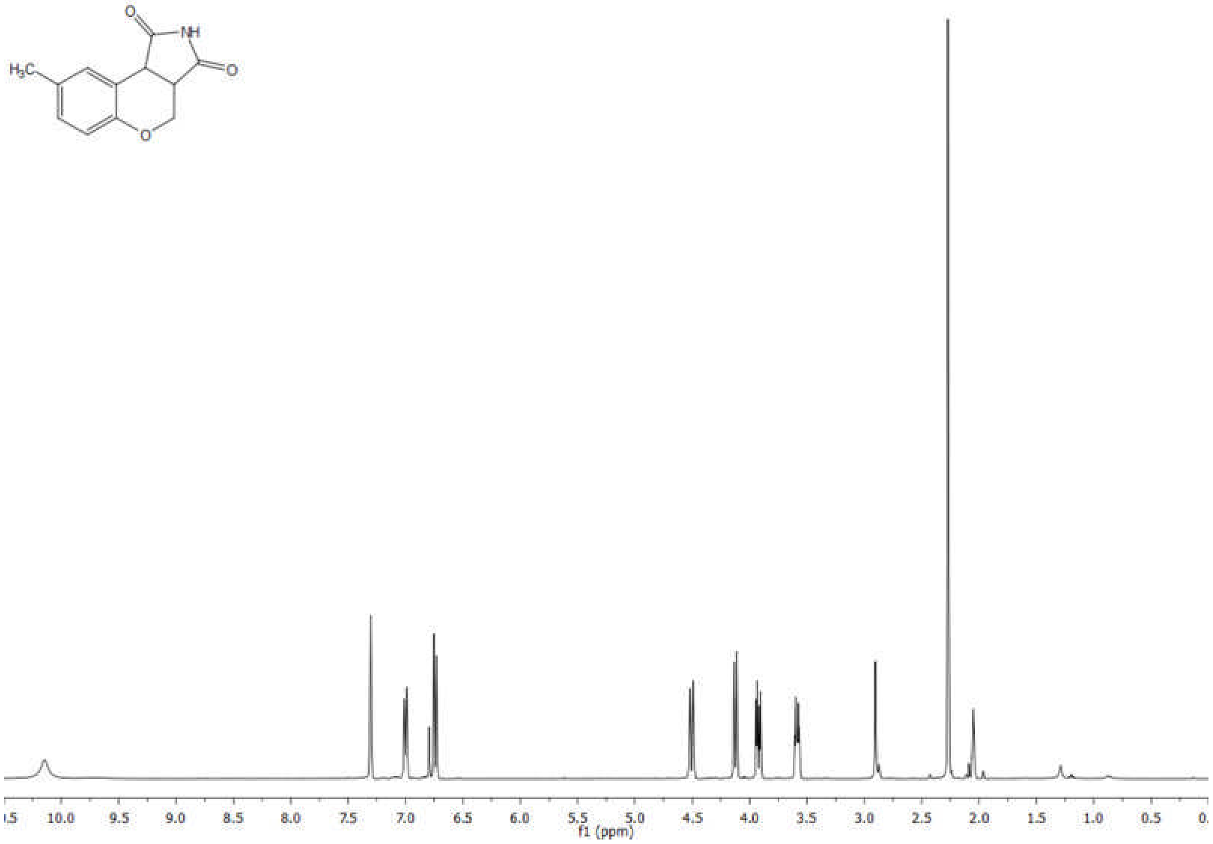

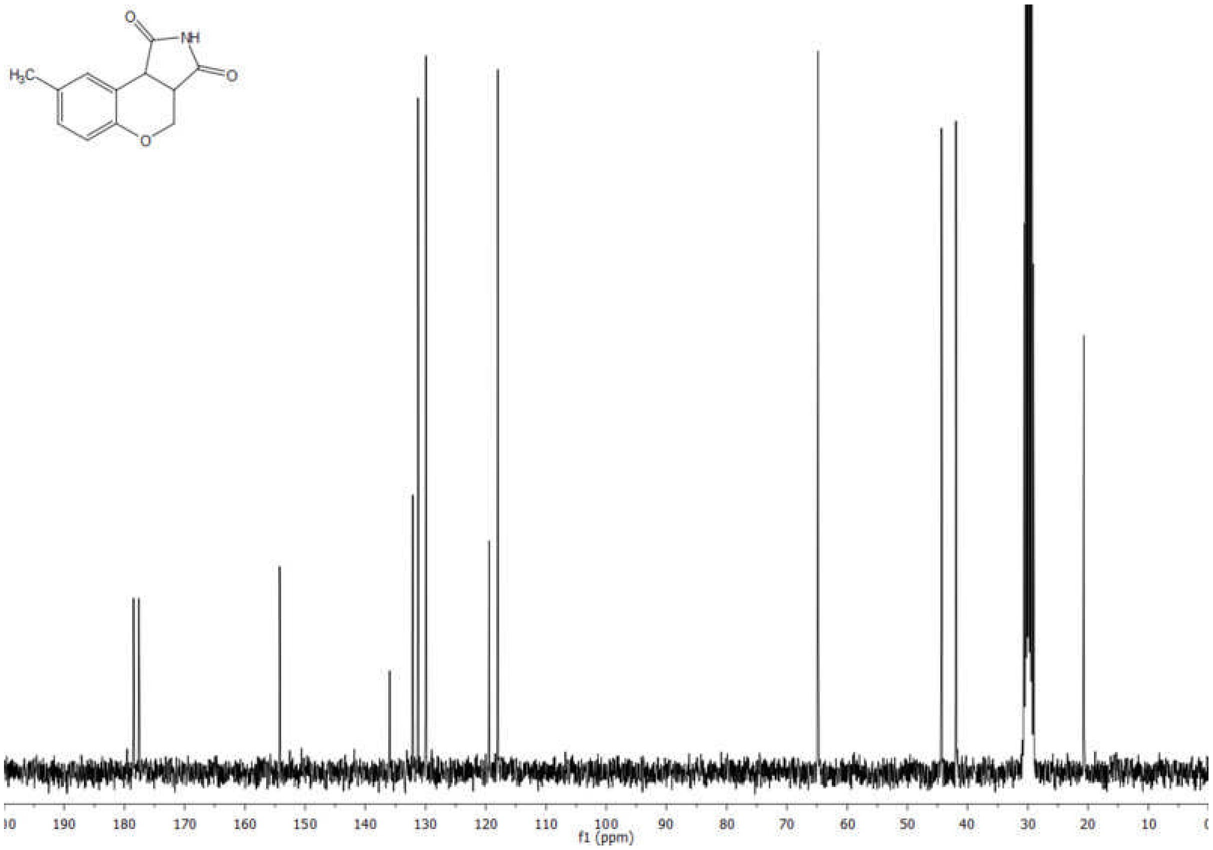

8-(*tert*-butyl)-3a,4-dihydrochromeno[3,4-c]pyrrole-1,3(2*H*,9b*H*)-dione 5c

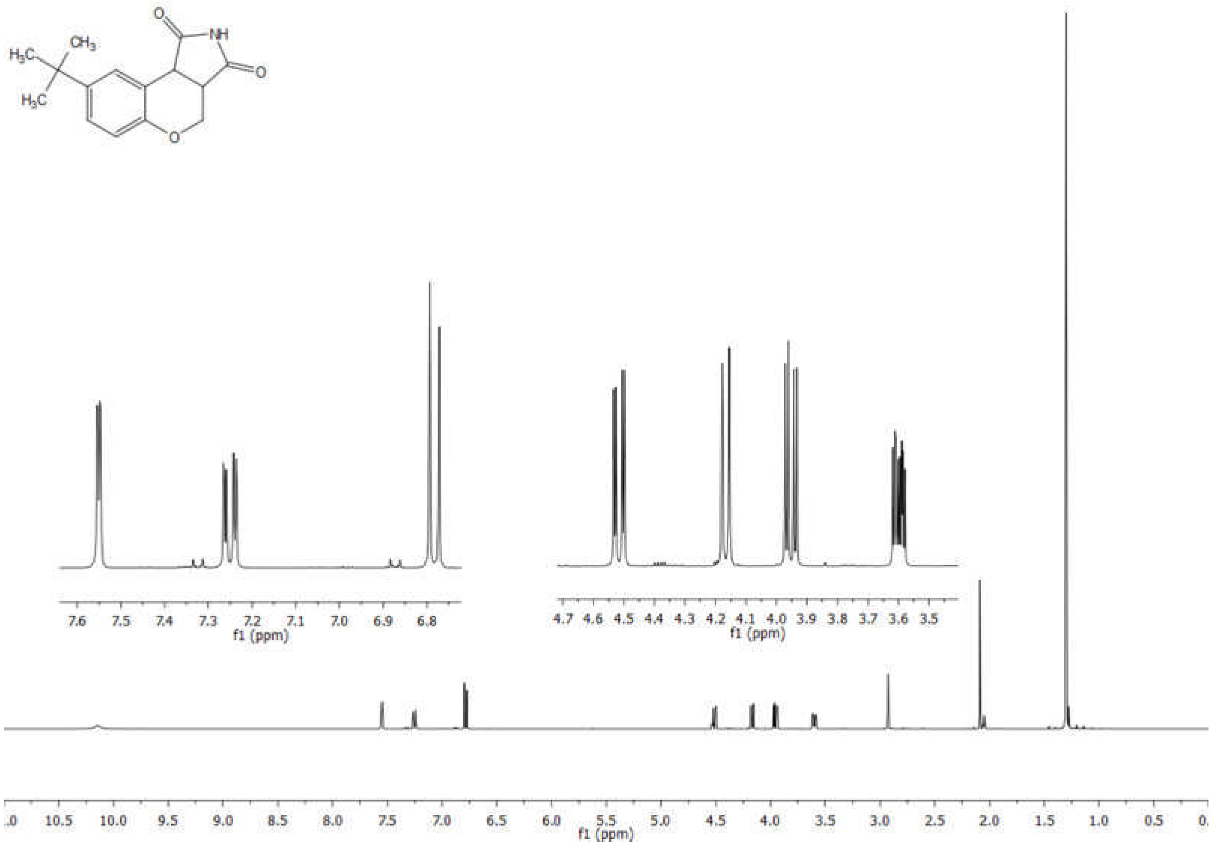

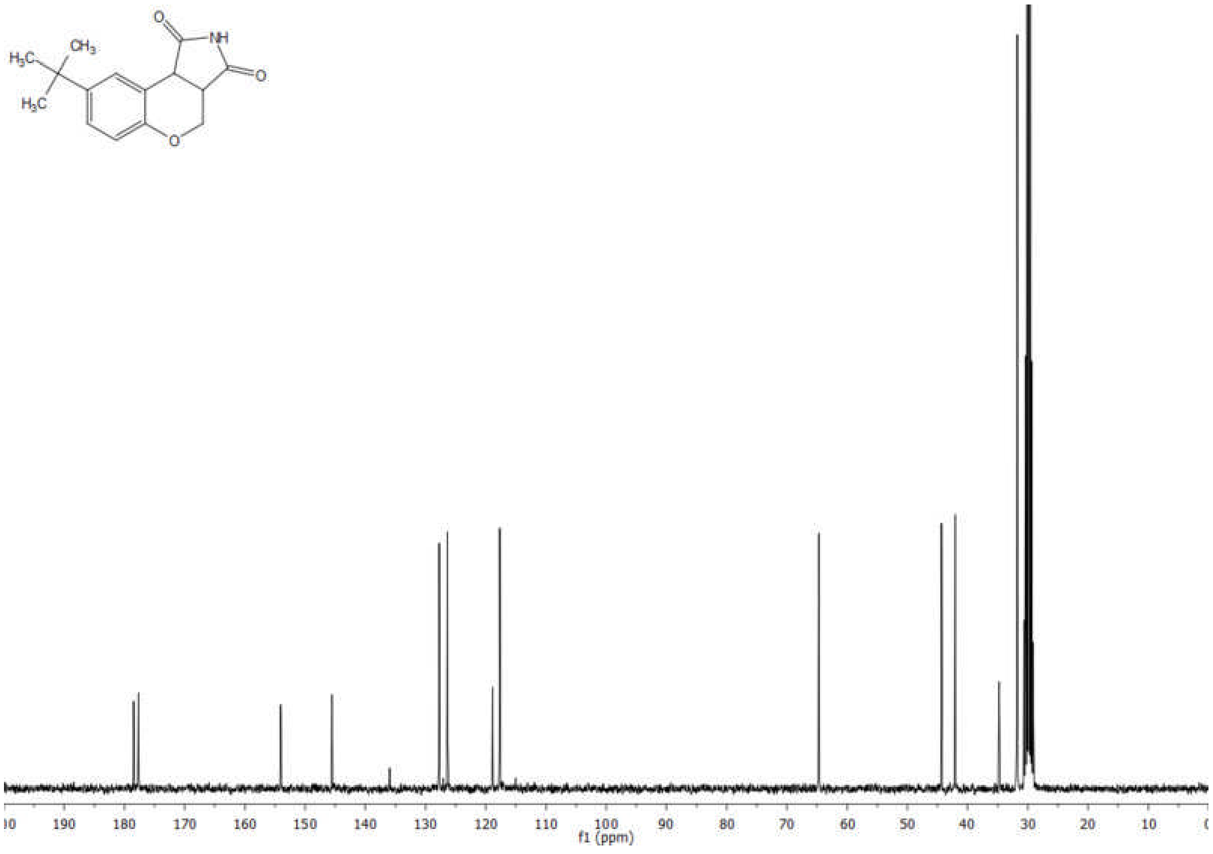

8-methoxy-3a,4-dihydrochromeno[3,4-*c*]pyrrole-1,3(2*H*,9b*H*)-dione 5d

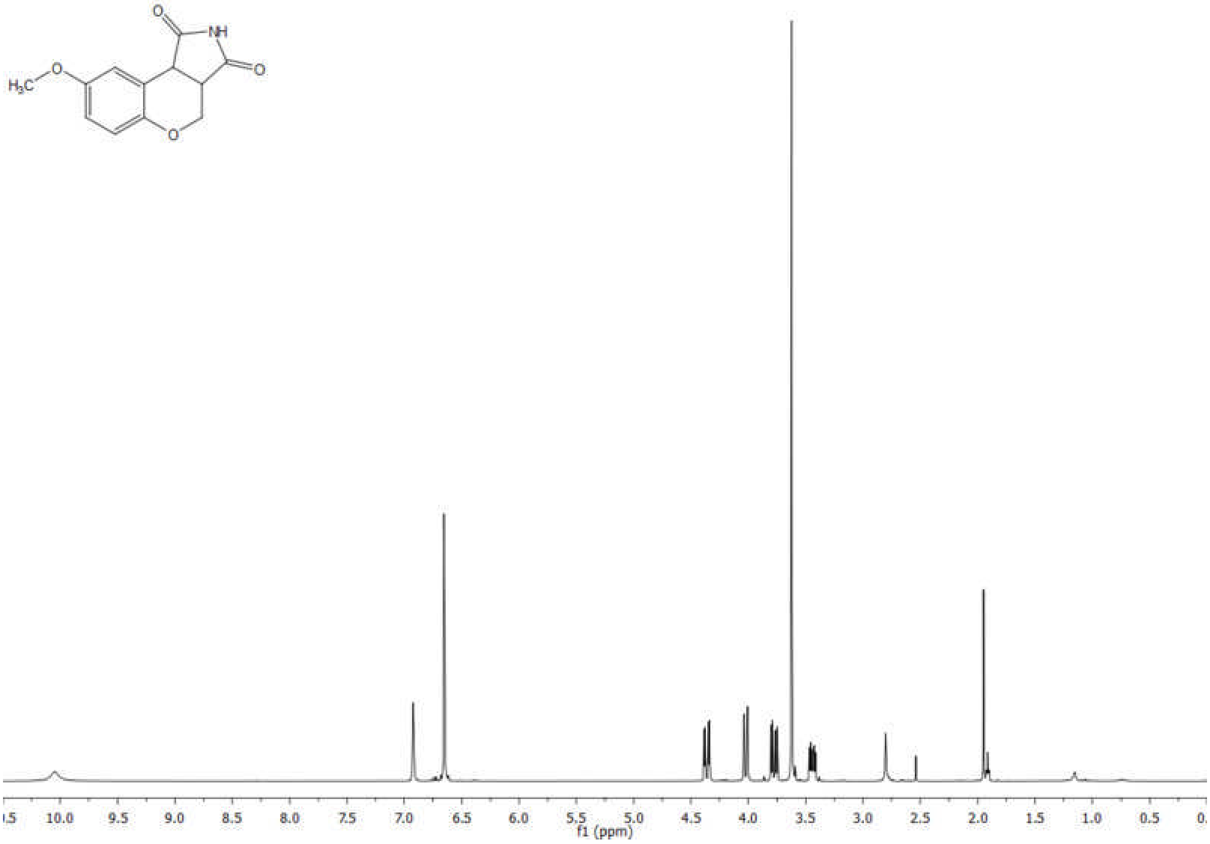

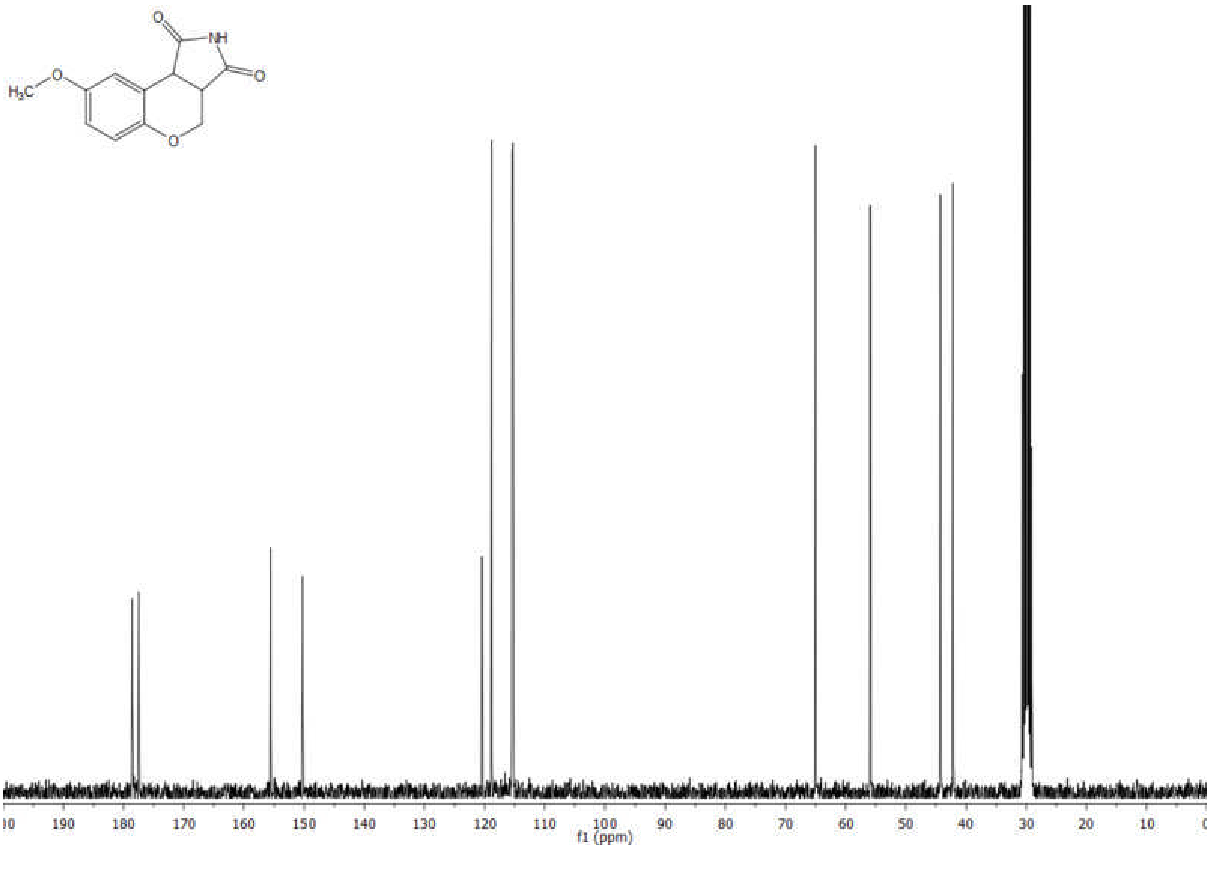

7-methoxy-3a,4-dihydrochromeno[3,4-*c*]pyrrole-1,3(2*H*,9b*H*)-dione 5e

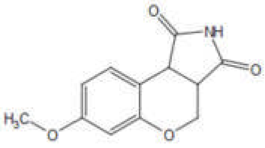

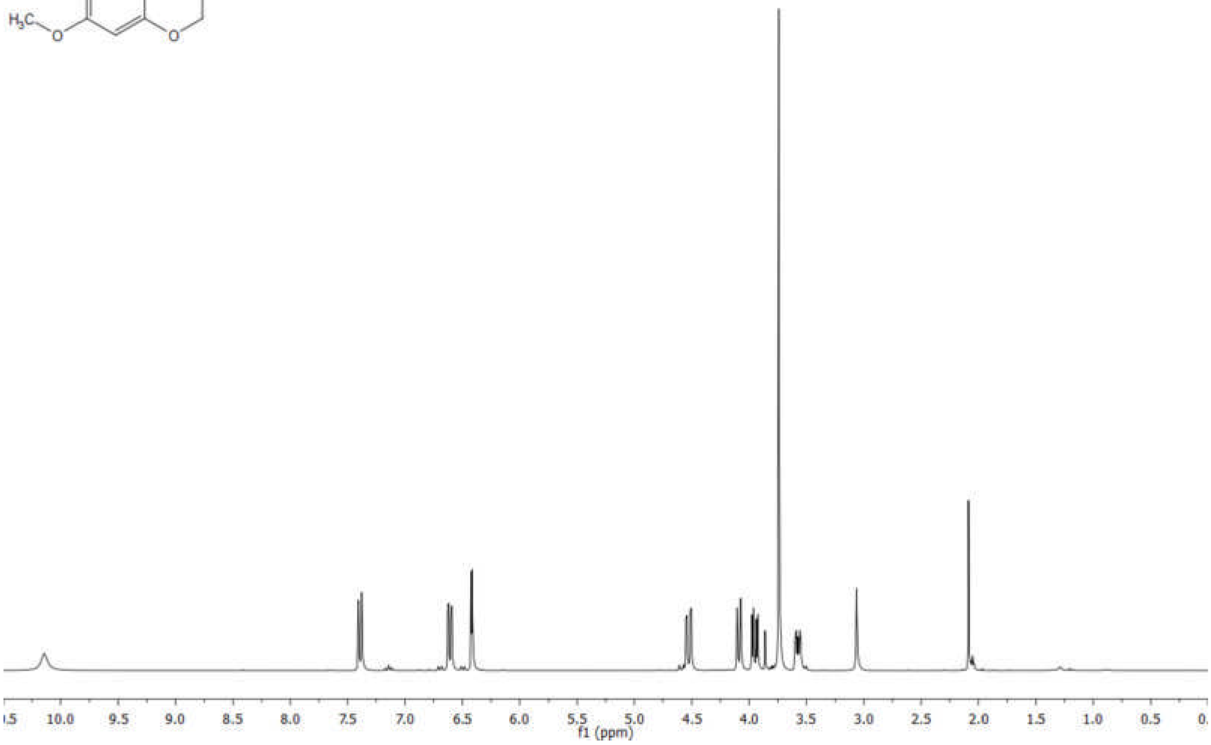

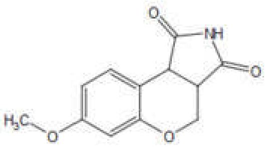

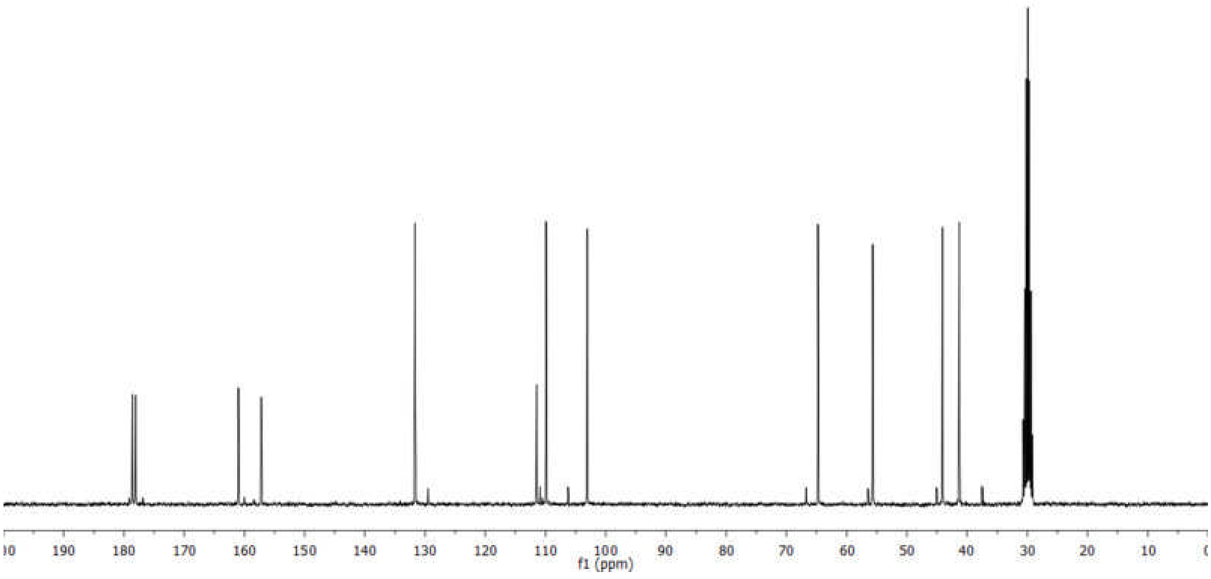

9-methoxy-3a,4-dihydrochromeno[3,4-*c*]pyrrole-1,3(2*H*,9b*H*)-dione 5e'

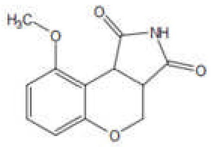

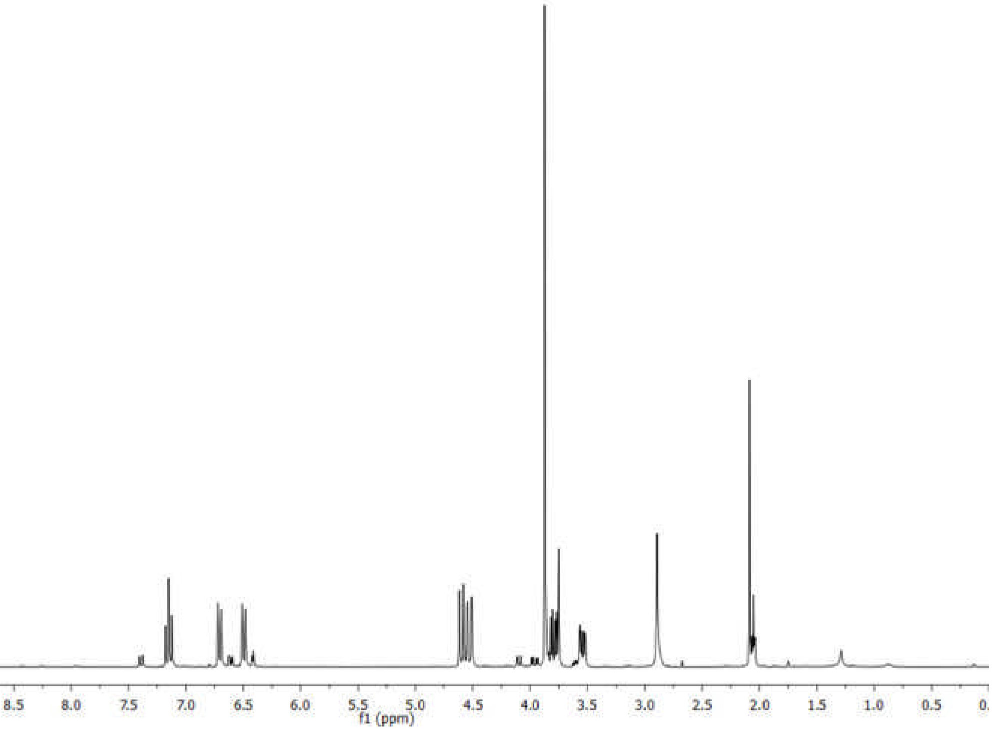

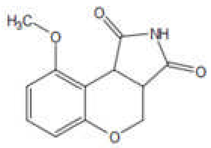

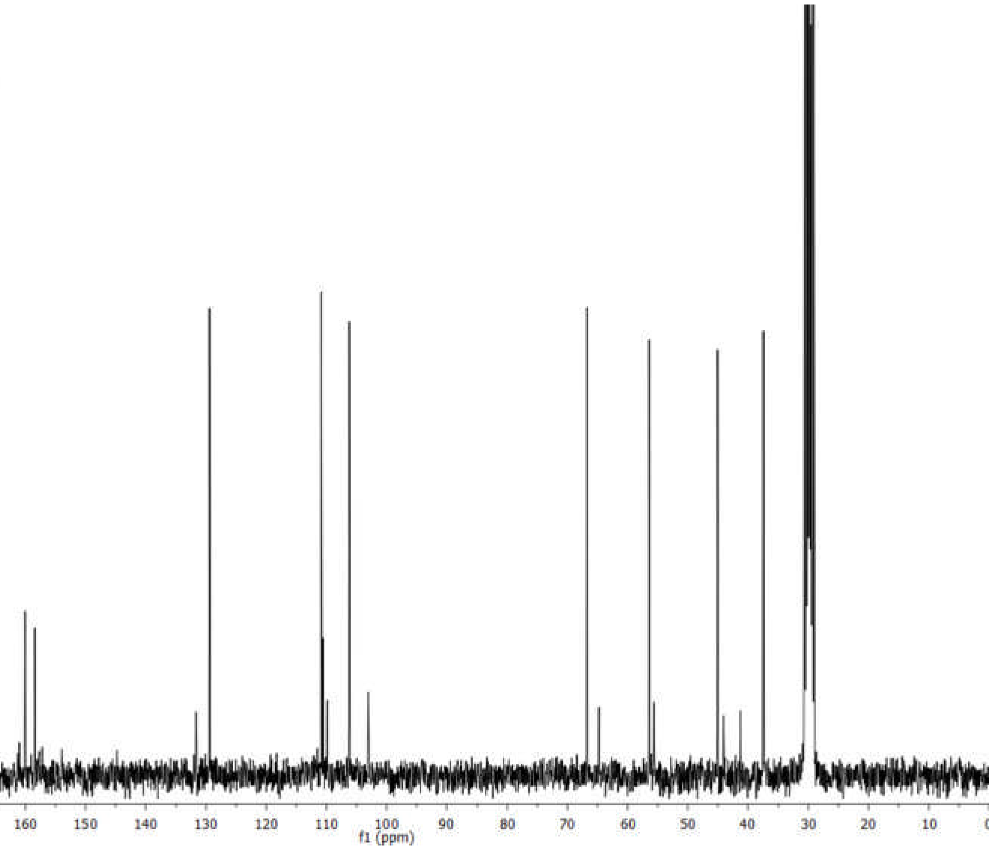

7,9-dimethoxy-3a,4-dihydrochromeno[3,4-*c*]pyrrole-1,3(2*H*,9b*H*)-dione 5g

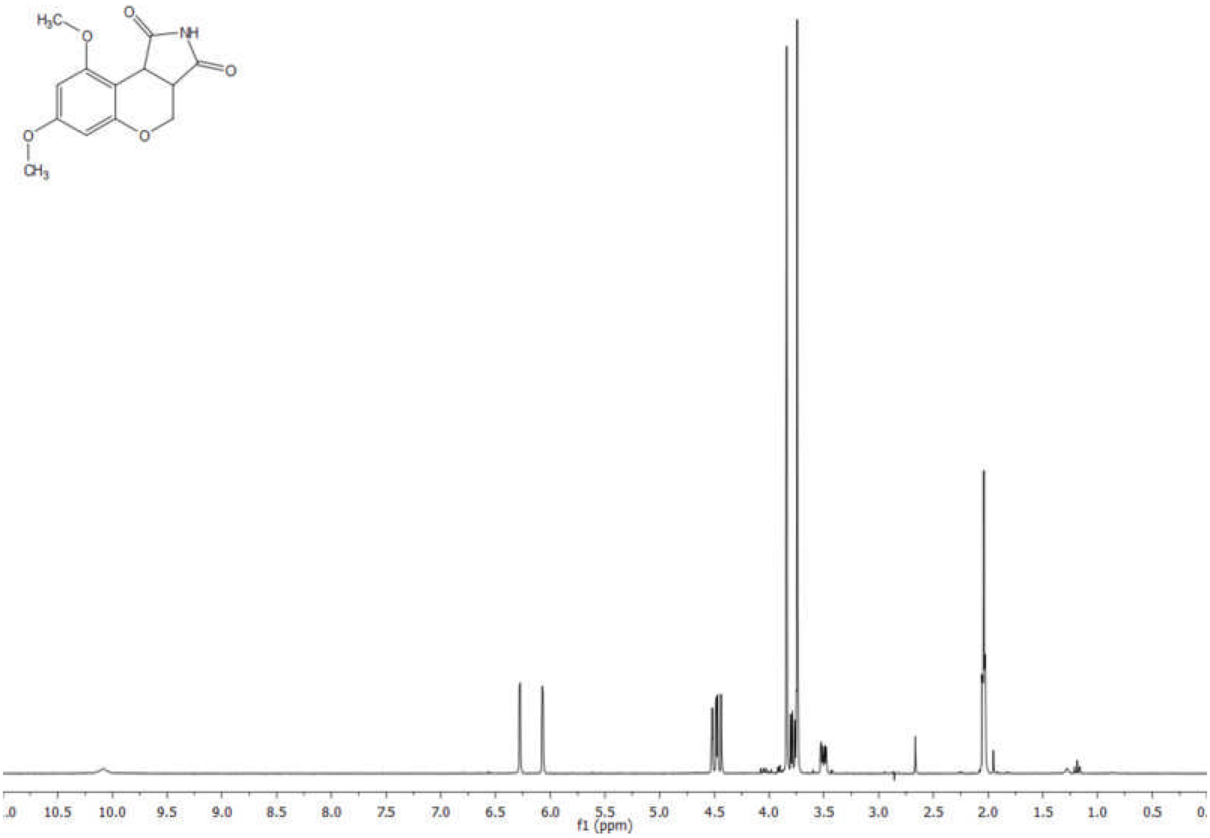

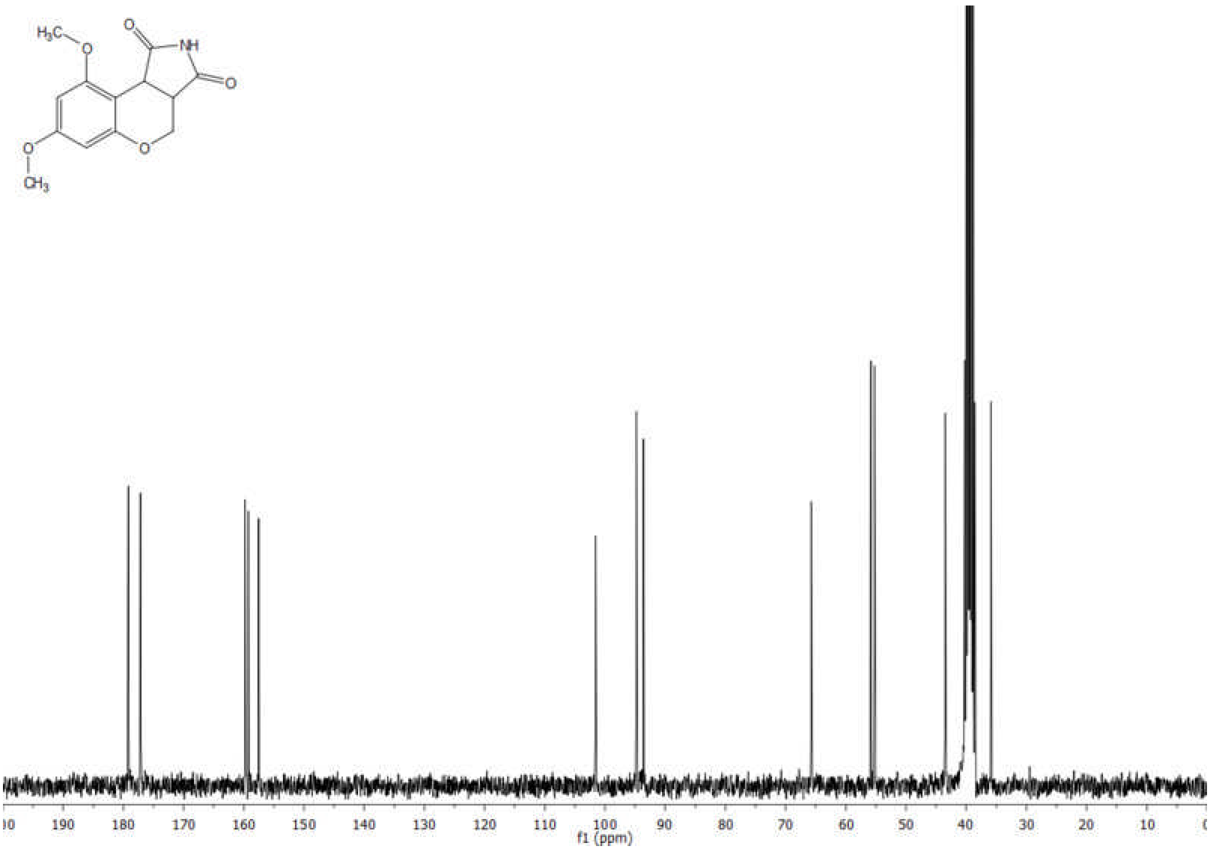

7,8,9-trimethoxy-3a,4-dihydrochromeno[3,4-*c*]pyrrole-1,3(2*H*,9b*H*)-dione 5h

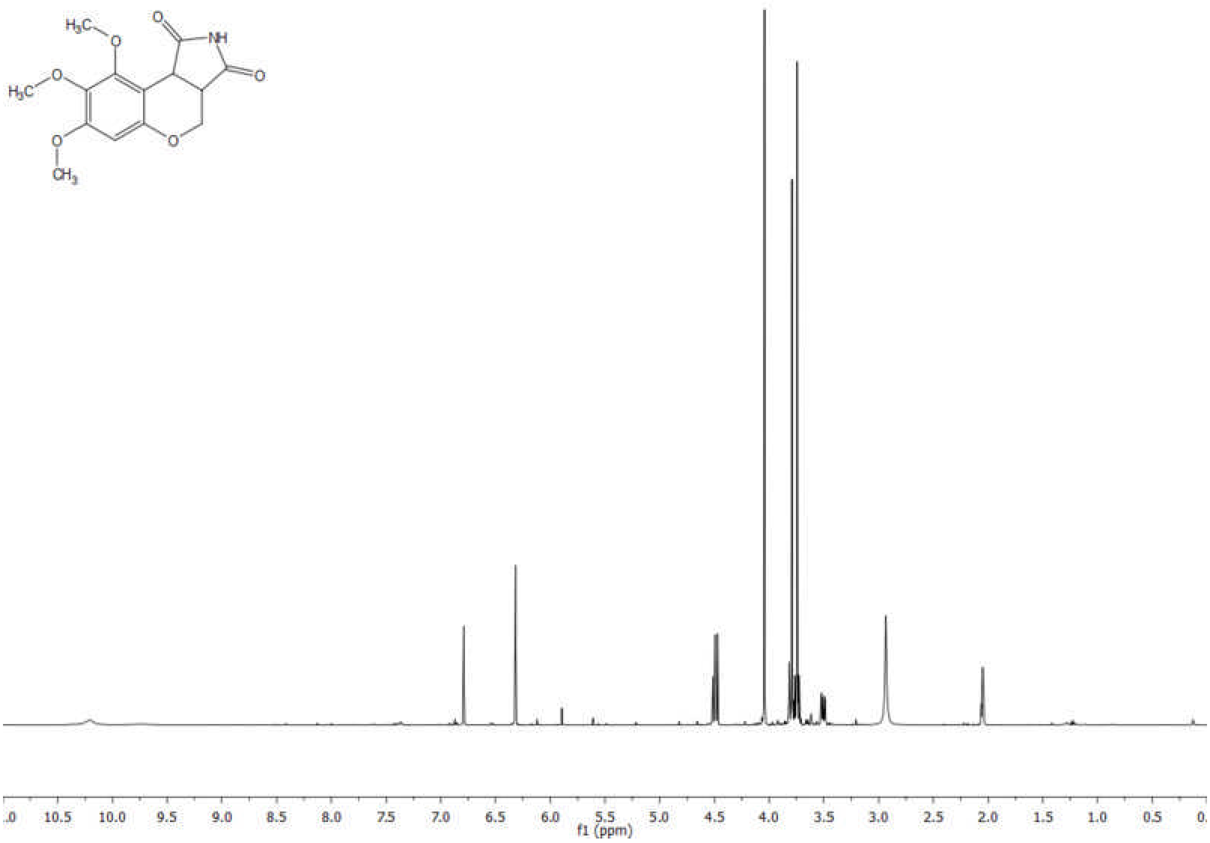

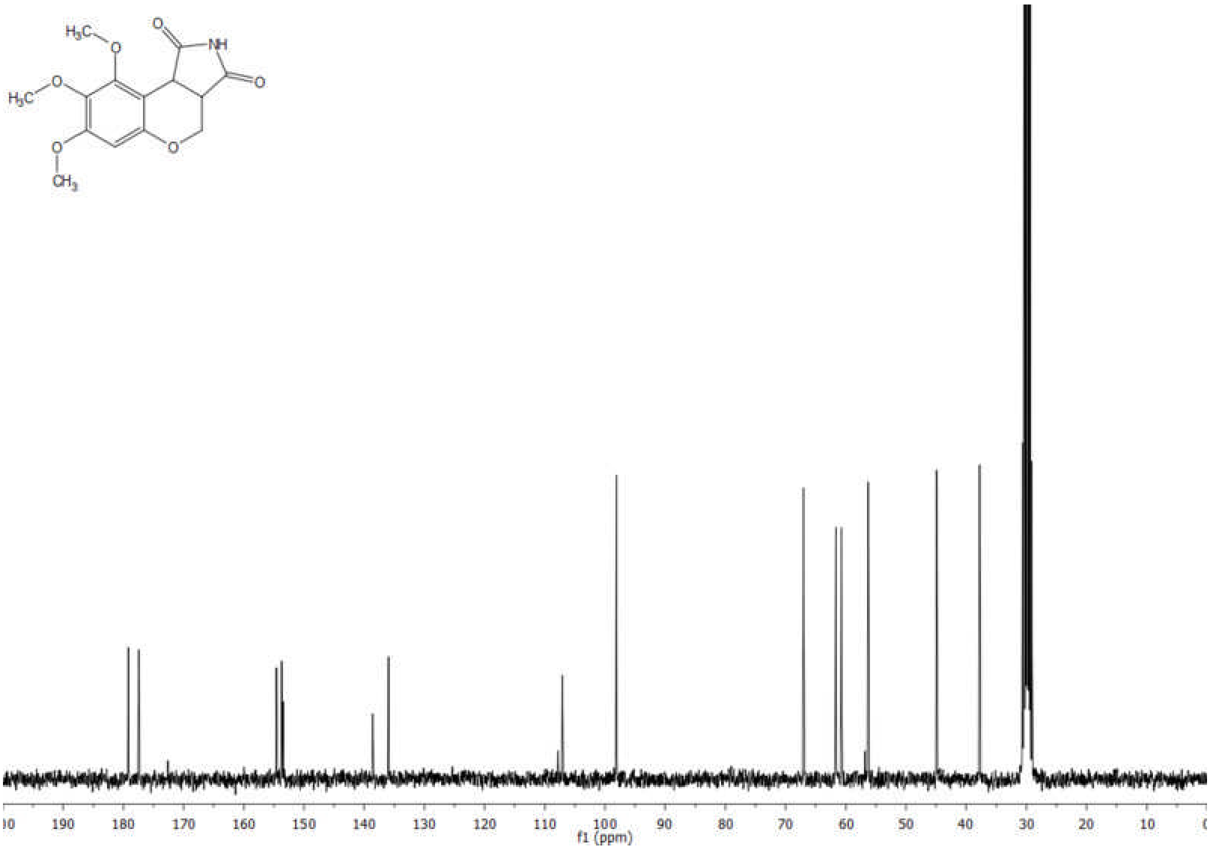

3a,4-dihydrothiochromeno[3,4-*c*]pyrrole-1,3(2*H*,9b*H*)-dione 5i

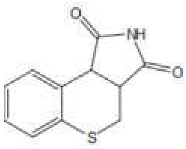

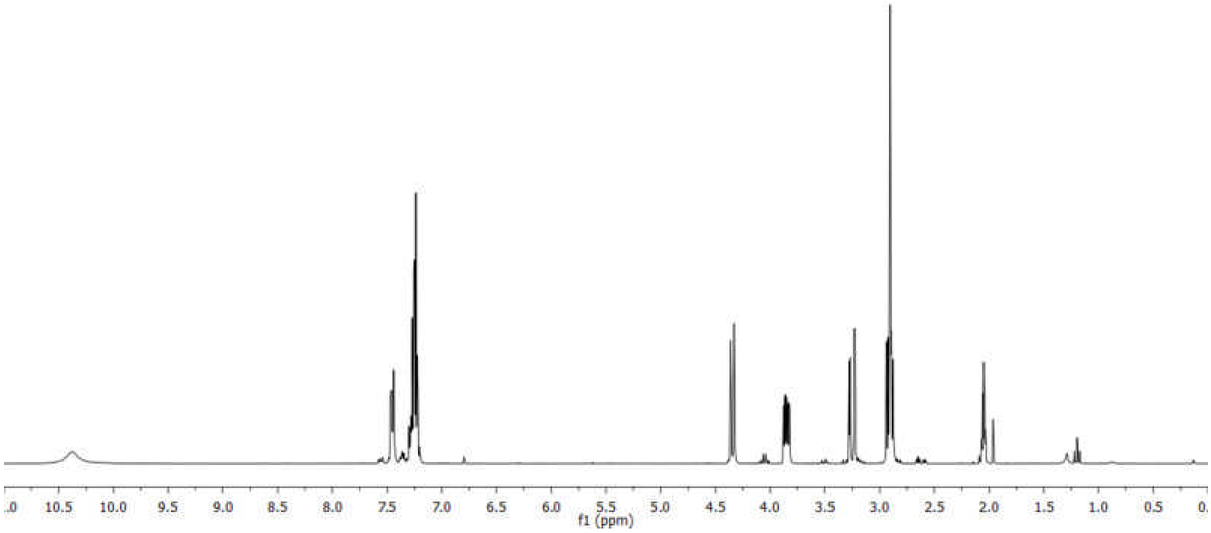

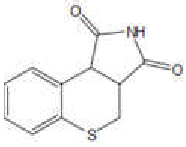

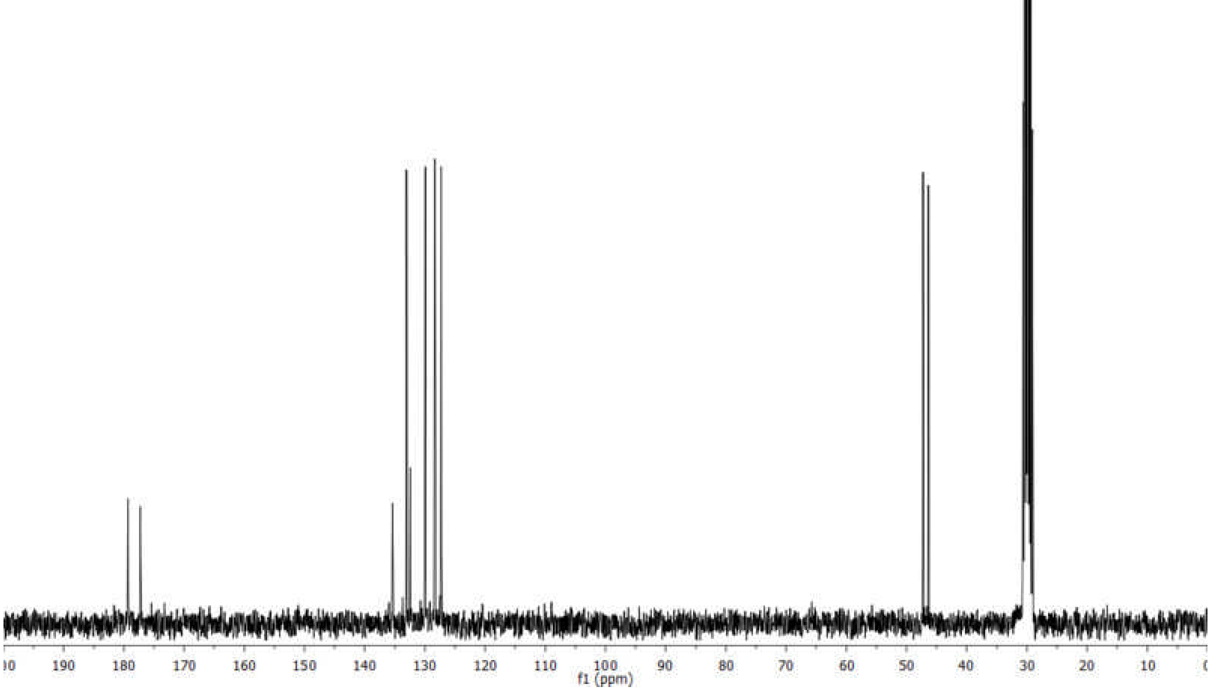

8-methoxy-3a,4-dihydrothiochromeno[3,4-c]pyrrole-1,3(2H,9bH)-dione 5j

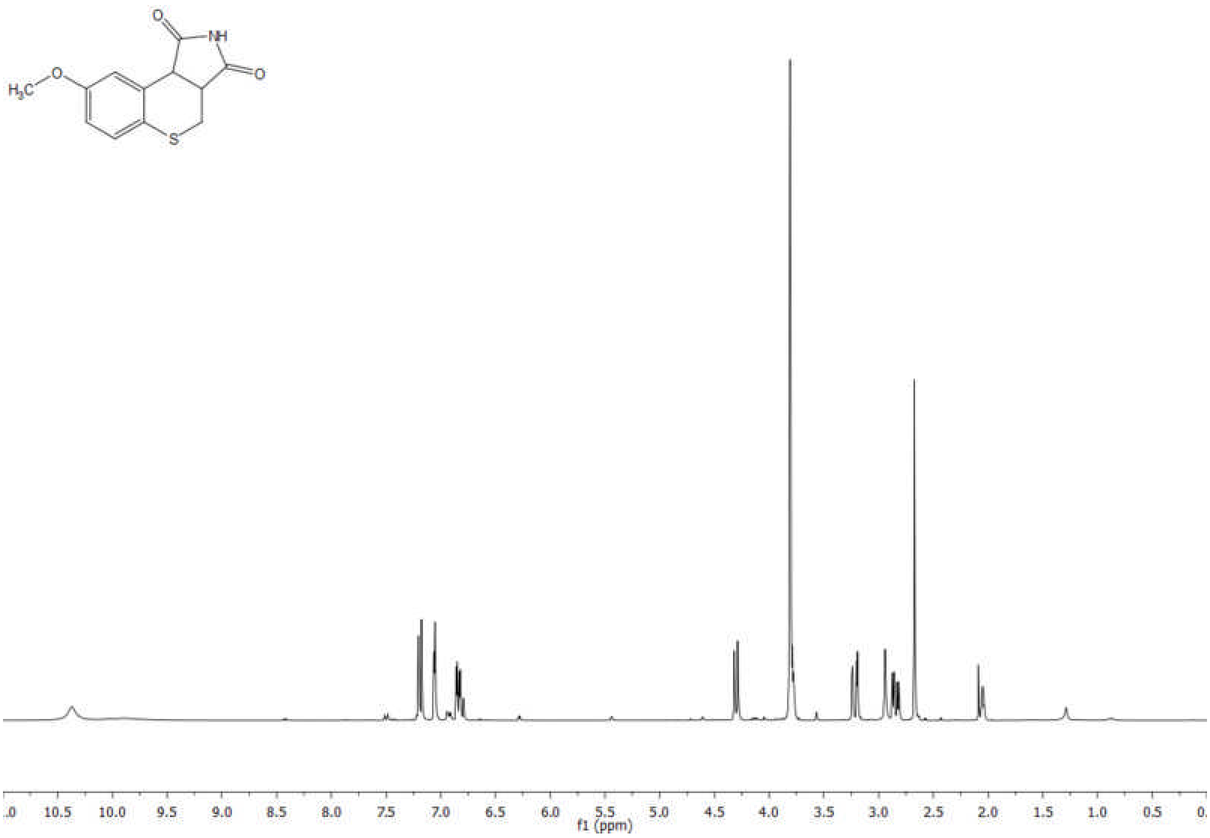

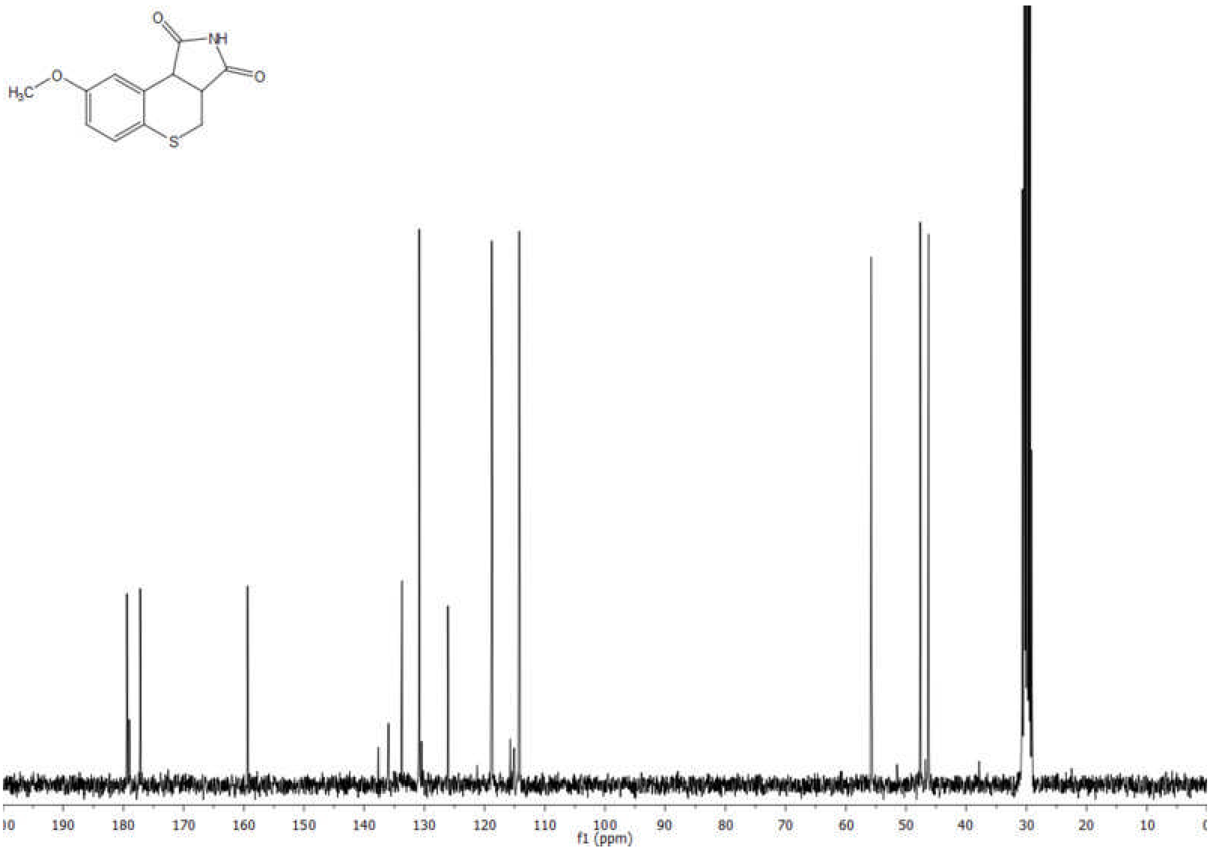

8-(methylthio)-3a,4-dihydrochromeno[3,4-c]pyrrole-1,3(2*H*,9b*H*)-dione 5k

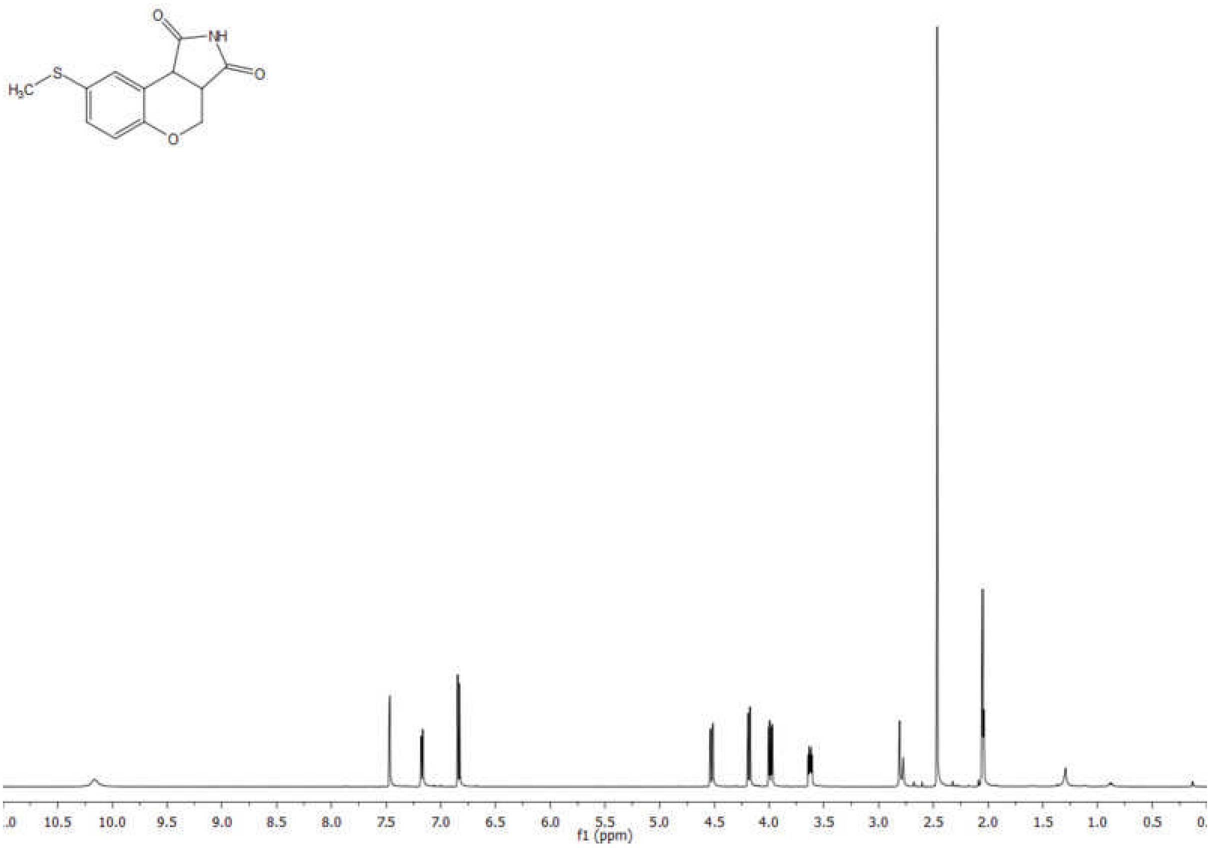

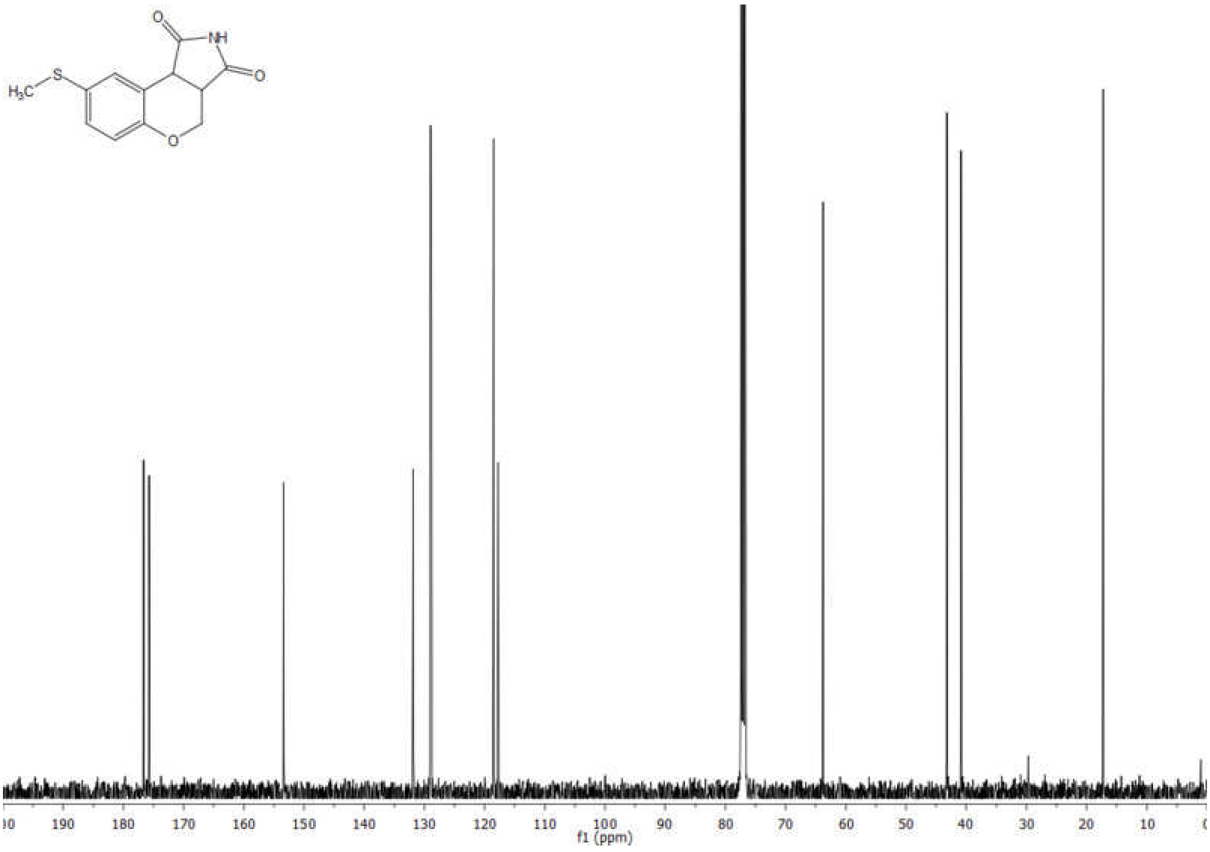

(3a*R*,9b*S*)-3a,4,5,9b-tetrahydro-1*H*-pyrrolo[3,4-*c*]quinoline-1,3(2*H*)-dione 4i

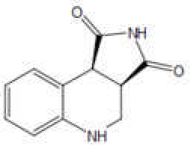

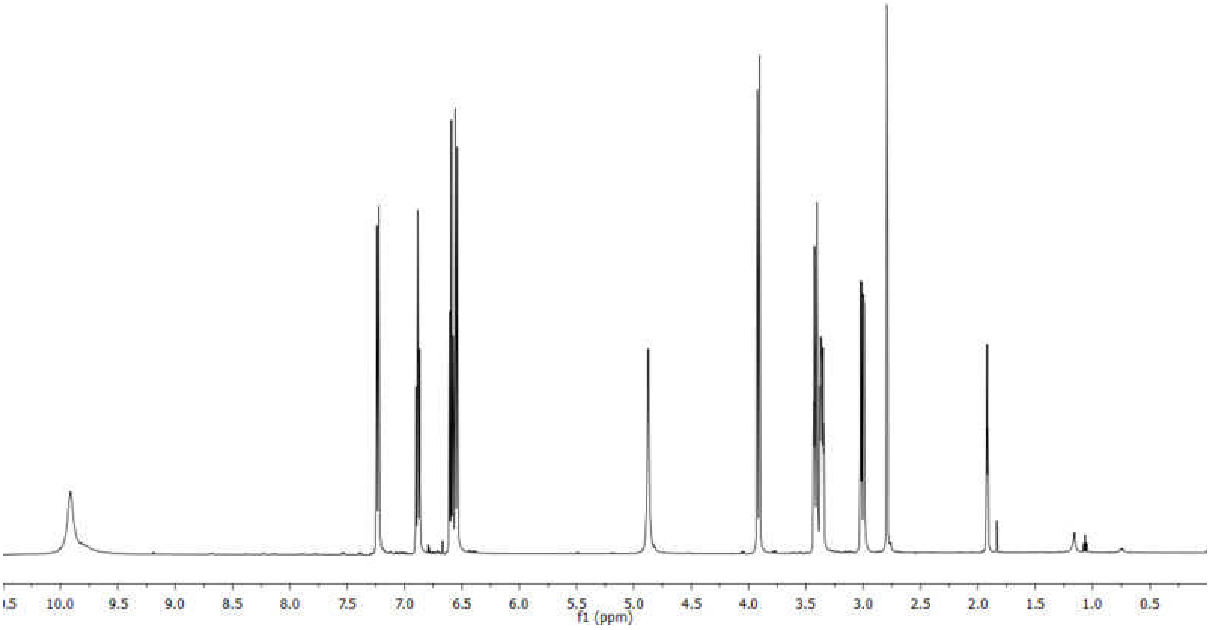

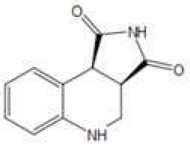

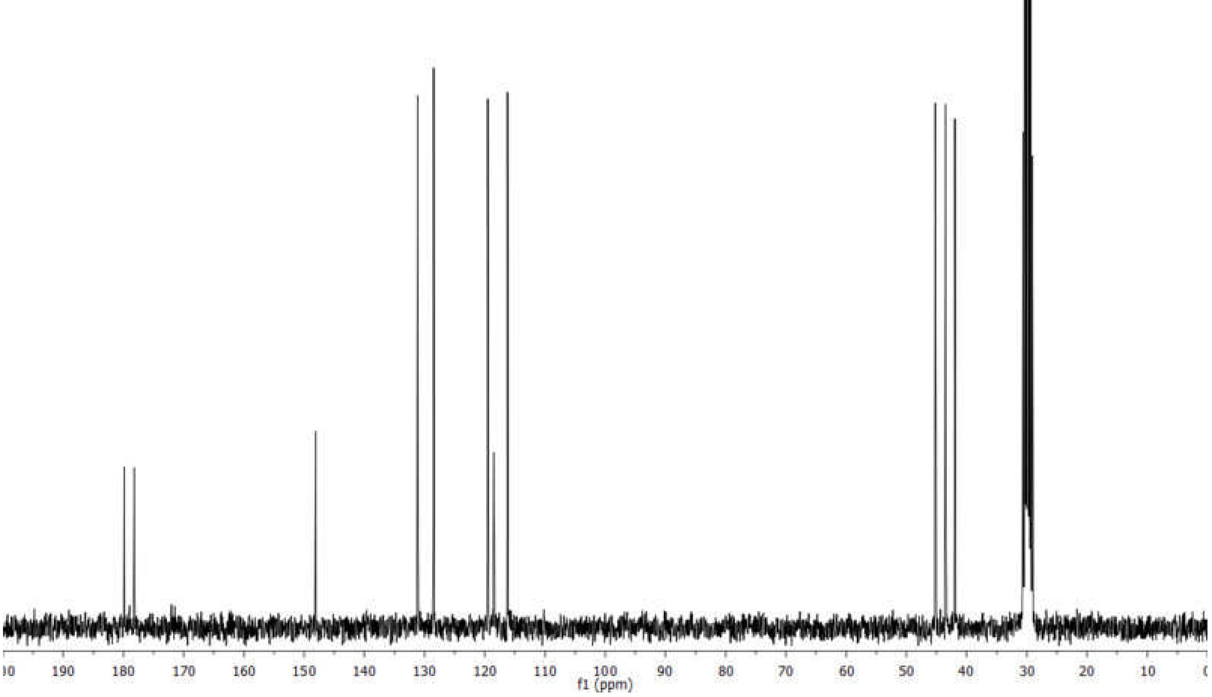

Benzyl (4-methoxyphenoxy)acetate 15

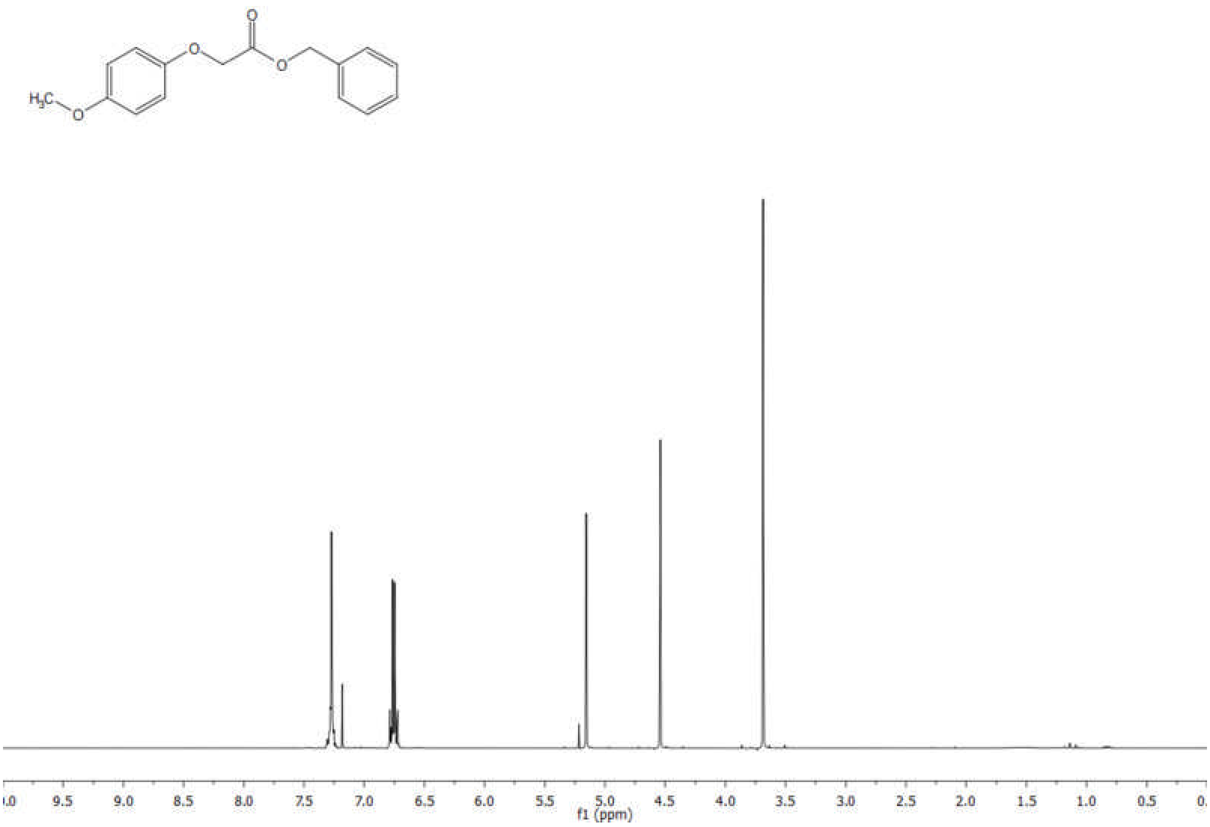

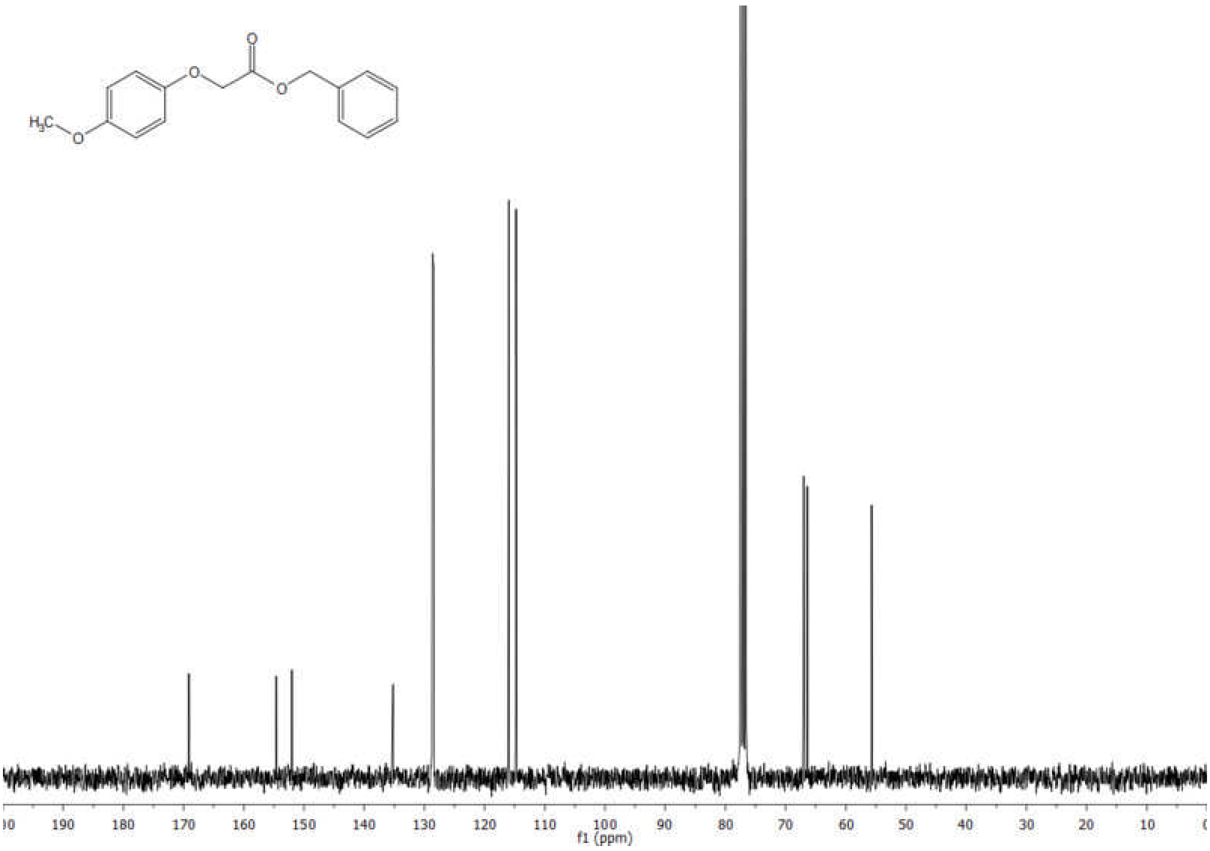

Dibenzyl 2,2'-(1,4-phenylenebis(oxy))diacetate 18

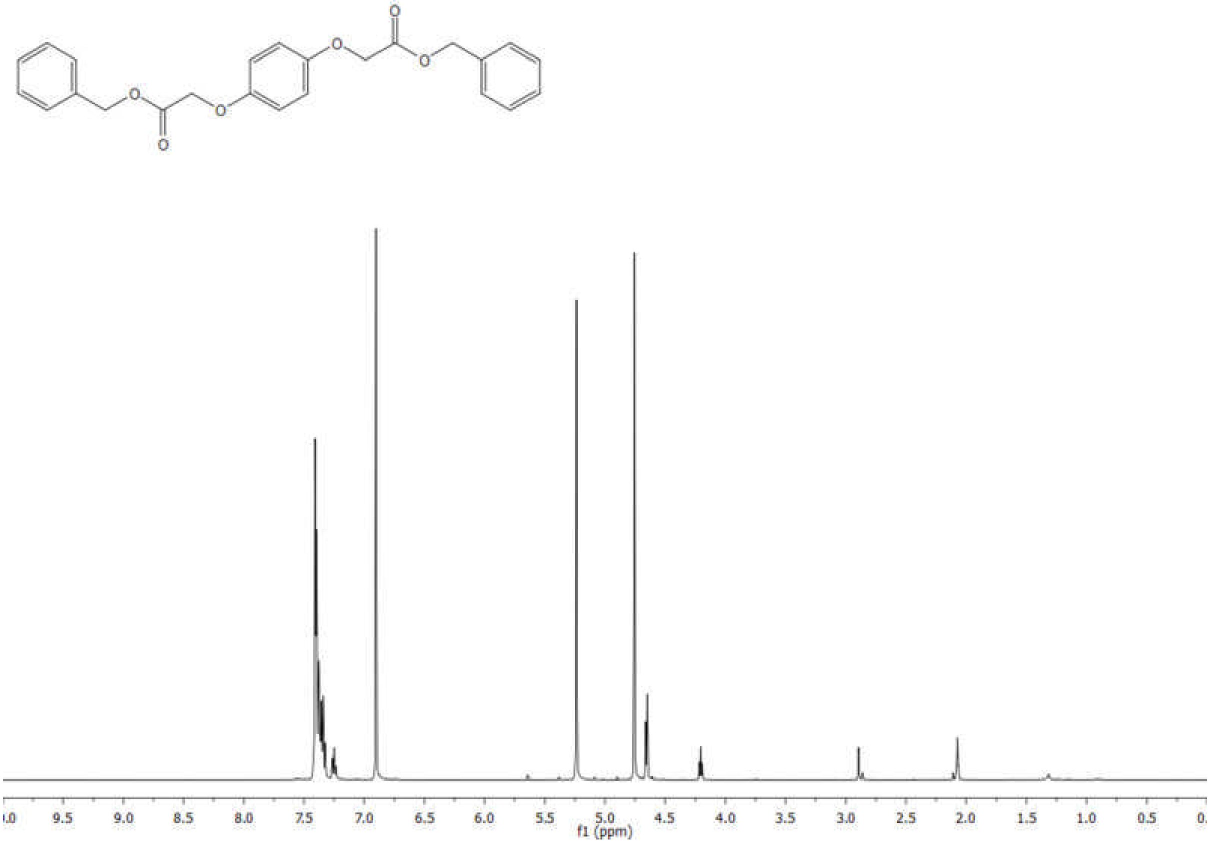

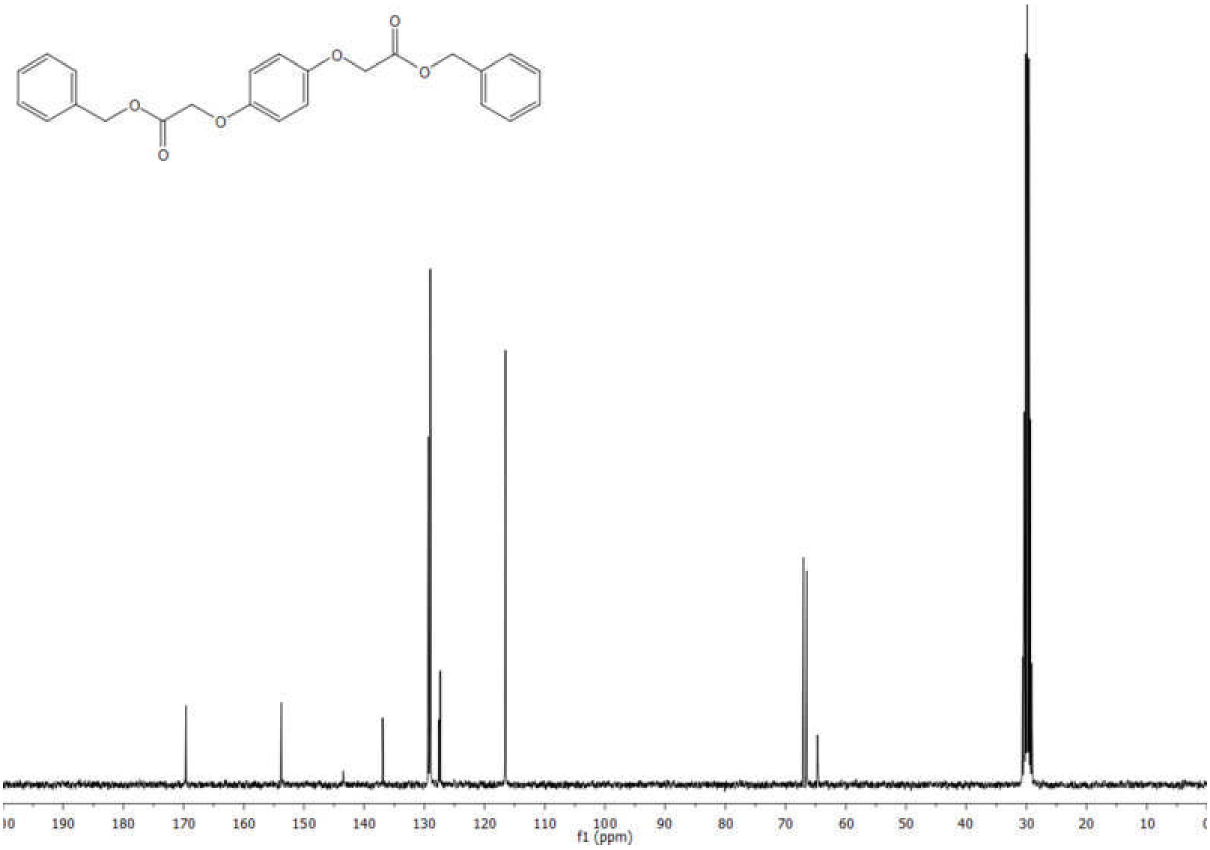

Supplement: Supplementary file 1 — miscellaneous_information [file chem0020-5492-sd1.pdf]
